# Supplementary material for: The COMA complex interacts with Cse4 and positions Sli15/Ipl1 at the budding yeast inner kinetochore
Source: eLife. 2019 May 21;8:e42879. doi: 10.7554/eLife.42879 (PMC6546395; doi:10.7554/eLife.42879)
Supplement: Figure 6—source data 1. [file elife-42879-fig6-data1.docx]

**Source data 1. Quantification of the minichromosome loss assay in a *SLI15*/*CTF19-FRB* strain.**

1. **Minichromosome loss of cells grown on synthetic medium containing 1 µg/ml rapamycin.**

| replicate no. | Anchor-away strain | Rescue plasmid | white colonies | red/red sectored colonies | total white + red colonies | % red/red sectored colonies | average% red/red sectored colonies | standard error |
| --- | --- | --- | --- | --- | --- | --- | --- | --- |
| replicate 1 | Ctf19-FRB | no | 0 | nd* | nd* | 100 | 100 | 0 |
| replicate 2 | Ctf19-FRB | no | 0 | nd* | nd* | 100 |  |  |
| replicate 3 | Ctf19-FRB | no | 0 | nd* | nd* | 100 |  |  |
| replicate 1 | Ctf19-FRB | Ctf19-WT | 513 | 131 | 644 | 20.3 | 18.8 | 0.4 |
| replicate 2 | Ctf19-FRB | Ctf19-WT | 514 | 102 | 616 | 16.6 |  |  |
| replicate 3 | Ctf19-FRB | Ctf19-WT | 500 | 120 | 620 | 19.4 |  |  |
| replicate 1 | Ctf19-FRB | Ctf19-Okp1 | 407 | 189 | 596 | 31.7 | 31.5 | 0.2 |
| replicate 2 | Ctf19-FRB | Ctf19-Okp1 | 548 | 240 | 788 | 30.5 |  |  |
| replicate 3 | Ctf19-FRB | Ctf19-Okp1 | 410 | 196 | 606 | 32.3 |  |  |
| replicate 1 | Ctf19-FRB | Ctf19∆C-Okp1 | 0 | nd* | nd* | 100 | 100 | 0 |
| replicate 2 | Ctf19-FRB | Ctf19∆C-Okp1 | 0 | nd* | nd* | 100 |  |  |
| replicate 3 | Ctf19-FRB | Ctf19∆C-Okp1 | 0 | nd* | nd* | 100 |  |  |
| replicate 1 | Ctf19-FRB | Okp1-Ctf19 | 0 | nd* | nd* | 100 | 100 | 0 |
| replicate 2 | Ctf19-FRB | Okp1-Ctf19 | 0 | nd* | nd* | 100 |  |  |
| replicate 3 | Ctf19-FRB | Okp1-Ctf19 | 0 | nd* | nd* | 100 |  |  |
| replicate 1 | Ctf19-FRB | Okp1-Ctf19∆C | 0 | nd* | nd* | 100 | 100 | 0 |
| replicate 2 | Ctf19-FRB | Okp1-Ctf19∆C | 0 | nd* | nd* | 100 |  |  |
| replicate 3 | Ctf19-FRB | Okp1-Ctf19∆C | 0 | nd* | nd* | 100 |  |  |

nd*: not determined, all colonies were red/red sectored

1. **Minichromosome loss of cells grown on synthetic medium lacking rapamycin.**

| replicate no. | Anchor-away strain | Rescue plasmid | white colonies | red/red sectored colonies | total white + red colonies | % red/red sectored colonies | average% red/red sectored colonies | standard error |
| --- | --- | --- | --- | --- | --- | --- | --- | --- |
| replicate 1 | Ctf19-FRB | no | 505 | 18 | 523 | 3.4 | 3.6 | 0.2 |
| replicate 2 | Ctf19-FRB | no | 866 | 30 | 896 | 3.3 |  |  |
| replicate 3 | Ctf19-FRB | no | 786 | 32 | 818 | 3.9 |  |  |
| replicate 1 | Ctf19-FRB | Ctf19-WT | 896 | 40 | 936 | 4.3 | 6.3 | 1.7 |
| replicate 2 | Ctf19-FRB | Ctf19-WT | 784 | 34 | 818 | 4.2 |  |  |
| replicate 3 | Ctf19-FRB | Ctf19-WT | 822 | 96 | 918 | 10.5 |  |  |
| replicate 1 | Ctf19-FRB | Ctf19-Okp1 | 607 | 45 | 652 | 6.9 | 6.9 | 0.5 |
| replicate 2 | Ctf19-FRB | Ctf19-Okp1 | 866 | 78 | 944 | 8.3 |  |  |
| replicate 3 | Ctf19-FRB | Ctf19-Okp1 | 1048 | 62 | 1110 | 5.6 |  |  |
| replicate 1 | Ctf19-FRB | Ctf19∆C-Okp1 | 644 | 72 | 716 | 10.1 | 7.3 | 0.8 |
| replicate 2 | Ctf19-FRB | Ctf19∆C-Okp1 | 724 | 46 | 770 | 6.0 |  |  |
| replicate 3 | Ctf19-FRB | Ctf19∆C-Okp1 | 974 | 60 | 1034 | 5.8 |  |  |
| replicate 1 | Ctf19-FRB | Okp1-Ctf19 | 239 | 129 | 368 | 35.1 | 25.5 | 2.3 |
| replicate 2 | Ctf19-FRB | Okp1-Ctf19 | 800 | 152 | 952 | 16.0 |  |  |
| replicate 3 | Ctf19-FRB | Okp1-Ctf19 | na* | na* | na* | na* |  |  |
| replicate 1 | Ctf19-FRB | Okp1-Ctf19∆C | 580 | 54 | 634 | 8.5 | 7.2 | 0.6 |
| replicate 2 | Ctf19-FRB | Okp1-Ctf19∆C | 1076 | 68 | 1144 | 5.9 |  |  |
| replicate 3 | Ctf19-FRB | Okp1-Ctf19∆C | na* | na* | na* | na* |  |  |

na*: not analyzed, experiment was discarded
