# Supplementary material for: The COMA complex interacts with Cse4 and positions Sli15/Ipl1 at the budding yeast inner kinetochore
Source: eLife. 2019 May 21;8:e42879. doi: 10.7554/eLife.42879 (PMC6546395; doi:10.7554/eLife.42879)
Supplement: Supplementary file 1. [file elife-42879-supp1.docx]

**Supplementary file 1. Inter- and intra-protein cross-links detected on *in vitro* reconstituted Cse4 containing nucleosomes interacting with the kinetochore complexes Ame1/Okp1, Ctf19/Mcm21, Mif2, Chl4/Iml3 and MTW1c.**

Ctf19/Mcm21, Mif2 and MTW1 complexes were recombinantly expressed in insect cells and affinity-purified via Mcm21-6xFLAG-6xHis, Mif2-6xFLAG-6xHis, or Dsn1-6xFLAG-6xHis, respectively. Ame1/Okp1, Chl4/Iml3 and Cse4 nucleosomes were expressed in *E. coli* and *in vitro* reconstituted as described (see materials and methods). A nearly stoichiometric supramolecular complex of Ame1/Okp1:Ctf19/Mcm21:Chl4/Iml3:Mif2:MTW1c and Cse4-NCPs was generated *in vitro* (see Figure S1) and chemical cross-links introduced by BS3 were identified as described (see materials and methods). In total, 349 inter-protein (marked in dark blue) and 395 intra-protein (marked in light blue) cross-links were detected on the recombinant complex (for a graphical representation of the cross-link network see Figure 1C).

| **No** | **Topology** | **Protein1** | **Protein2** | **AbsPos1** | **AbsPos2** | **Mz** | **z** | **Error** | **mions** | **Nseen** | **ld.Score** |
| --- | --- | --- | --- | --- | --- | --- | --- | --- | --- | --- | --- |
| 1 | DFNKSDDDQFRK-AKQLLATR-a4-b2 | AME1 | CTF19 | 160 | 229 | 638,832 | 4 | -1,9 | 5 | 1 | 25,64 |
| 2 | LNDLTSTLLGKYEGDR-ISIALWKGGER-a11-b7 | AME1 | CTF19 | 266 | 327 | 791,178 | 4 | 0,7 | 12 | 2 | 36,48 |
| 3 | LNDLTSTLLGKYEGDRK-ISIALWKGGER-a11-b7 | AME1 | CTF19 | 266 | 327 | 658,761 | 5 | -1,1 | 15 | 12 | 36,21 |
| 4 | RLNDLTSTLLGKYEGDR-ISIALWKGGER-a12-b7 | AME1 | CTF19 | 266 | 327 | 830,204 | 4 | 2 | 12 | 2 | 35,28 |
| 5 | LNDLTSTLLGKYEGDRK-ISIALWKGGER-a11-b7 | AME1 | CTF19 | 266 | 327 | 658,762 | 5 | 0,1 | 14 | 4 | 34,77 |
| 6 | LNDLTSTLLGKYEGDRK-ISIALWKGGER-a11-b7 | AME1 | CTF19 | 266 | 327 | 658,761 | 5 | -2,2 | 11 | 2 | 31,72 |
| 7 | LNDLTSTLLGKYEGDRK-ISIALWKGGER-a11-b7 | AME1 | CTF19 | 266 | 327 | 658,762 | 5 | -0,7 | 11 | 1 | 29,94 |
| 8 | NEIQELKAGK-KSAKATK-a7-b1 | AME1 | H2A1 | 231 | 121 | 500,792 | 4 | 2,7 | 6 | 1 | 28,58 |
| 9 | DTKLAFR-IYSKK-a3-b4 | AME1 | OKP1 | 6 | 202 | 542,647 | 3 | -0,7 | 5 | 2 | 30,86 |
| 10 | DFNKSDDDQFR-IYSKK-a4-b4 | AME1 | OKP1 | 160 | 202 | 721,351 | 3 | -1,1 | 7 | 4 | 39,31 |
| 11 | DFNKSDDDQFRK-FEVGKESTGK-a4-b5 | AME1 | OKP1 | 160 | 103 | 911,772 | 3 | 0,3 | 15 | 9 | 37,99 |
| 12 | DFNKSDDDQFRK-IYSKK-a4-b4 | AME1 | OKP1 | 160 | 202 | 573,289 | 4 | -1 | 7 | 8 | 37,65 |
| 13 | DFNKSDDDQFRK-FEVGKESTGK-a4-b5 | AME1 | OKP1 | 160 | 103 | 911,773 | 3 | 0,8 | 11 | 7 | 36,89 |
| 14 | DFNKSDDDQFR-IYSKK-a4-b4 | AME1 | OKP1 | 160 | 202 | 721,352 | 3 | -0,6 | 6 | 6 | 36,82 |
| 15 | DFNKSDDDQFRK-IYSKK-a4-b4 | AME1 | OKP1 | 160 | 202 | 764,05 | 3 | -0,8 | 8 | 12 | 36,55 |
| 16 | DFNKSDDDQFR-RIYSKK-a4-b5 | AME1 | OKP1 | 160 | 202 | 773,386 | 3 | 0,7 | 7 | 2 | 35,89 |
| 17 | DFNKSDDDQFRK-TNKEEGQYHHK-a4-b3 | AME1 | OKP1 | 160 | 84 | 605,284 | 5 | -0,7 | 11 | 6 | 33,49 |
| 18 | DFNKSDDDQFRK-GSKQLR-a4-b3 | AME1 | OKP1 | 160 | 95 | 585,795 | 4 | -1,5 | 7 | 4 | 33,2 |
| 19 | DFNKSDDDQFRK-GSKQLR-a4-b3 | AME1 | OKP1 | 160 | 95 | 585,796 | 4 | 0,6 | 8 | 5 | 31,95 |
| 20 | DFNKSDDDQFRK-GSKQLR-a4-b3 | AME1 | OKP1 | 160 | 95 | 468,837 | 5 | -1,8 | 7 | 4 | 30,43 |
| 21 | DFNKSDDDQFRK-KVIQAEYR-a4-b1 | AME1 | OKP1 | 160 | 132 | 886,775 | 3 | -2,9 | 7 | 2 | 29,35 |
| 22 | DFNKSDDDQFRK-IYSKK-a4-b4 | AME1 | OKP1 | 160 | 202 | 573,29 | 4 | 0,9 | 4 | 1 | 28,86 |
| 23 | DFNKSDDDQFRK-FEVGKESTGK-a4-b5 | AME1 | OKP1 | 160 | 103 | 547,467 | 5 | 1,7 | 7 | 3 | 28,57 |
| 24 | DFNKSDDDQFR-RIYSKK-a4-b5 | AME1 | OKP1 | 160 | 202 | 773,386 | 3 | 0,4 | 4 | 1 | 28,22 |
| 25 | LLYKLDLR-GSKQLR-a4-b3 | AME1 | OKP1 | 172 | 95 | 465,533 | 4 | -1 | 6 | 2 | 39,32 |
| 26 | LLYKLDLR-GSKQLR-a4-b3 | AME1 | OKP1 | 172 | 95 | 465,534 | 4 | 0 | 7 | 2 | 37,93 |
| 27 | LLYKLDLR-STQSKK-a4-b5 | AME1 | OKP1 | 172 | 62 | 463,026 | 4 | 0 | 7 | 2 | 34,27 |
| 28 | LLYKLDLR-QAKFPSR-a4-b3 | AME1 | OKP1 | 172 | 222 | 501,797 | 4 | 0,1 | 7 | 2 | 33,75 |
| 29 | LLYKLDLR-IYSKK-a4-b4 | AME1 | OKP1 | 172 | 202 | 603,702 | 3 | 0,6 | 6 | 4 | 33,71 |
| 30 | LLYKLDLR-STQSKK-a4-b5 | AME1 | OKP1 | 172 | 62 | 463,025 | 4 | -2,2 | 6 | 2 | 31,97 |
| 31 | LLYKLDLR-STQSKK-a4-b5 | AME1 | OKP1 | 172 | 62 | 463,025 | 4 | -1,5 | 5 | 2 | 31,02 |
| 32 | DILDINVSNNELCYQLKQVLAR-KVIQAEYR-a17-b1 | AME1 | OKP1 | 207 | 132 | 941,254 | 4 | 0,2 | 7 | 6 | 33,82 |
| 33 | DILDINVSNNELCYQLKQVLAR-TNKEEGQYHHK-a17-b3 | AME1 | OKP1 | 207 | 84 | 1376,031 | 3 | 2,6 | 5 | 2 | 22,85 |
| 34 | KEDLNQQIISVR-KVIQAEYR-a1-b1 | AME1 | OKP1 | 213 | 132 | 647,362 | 4 | 0,2 | 8 | 4 | 30,88 |
| 35 | KEDLNQQIISVR-NYELKHWK-a1-b5 | AME1 | OKP1 | 213 | 149 | 899,816 | 3 | -1,2 | 6 | 1 | 26,03 |
| 36 | KEDLNQQIISVR-LCMNLKTNNK-a1-b6 | AME1 | OKP1 | 213 | 280 | 704,624 | 4 | -2,8 | 5 | 1 | 22,23 |
| 37 | NEIQELKAGK-LTEKLIQK-a7-b4 | AME1 | OKP1 | 231 | 290 | 560,579 | 4 | 0,7 | 11 | 5 | 38,22 |
| 38 | NEIQELKAGK-LTEKLIQK-a7-b4 | AME1 | OKP1 | 231 | 290 | 747,101 | 3 | -2,2 | 10 | 8 | 37,35 |
| 39 | NEIQELKAGK-KLCMNLK-a7-b1 | AME1 | OKP1 | 231 | 274 | 544,048 | 4 | -1,8 | 9 | 19 | 37,07 |
| 40 | NEIQELKAGK-RLTEKLIQK-a7-b5 | AME1 | OKP1 | 231 | 290 | 799,138 | 3 | 2,7 | 10 | 13 | 35,64 |
| 41 | NEIQELKAGK-KLCMNLK-a7-b1 | AME1 | OKP1 | 231 | 274 | 544,048 | 4 | -1,2 | 7 | 5 | 33,02 |
| 42 | NEIQELKAGK-KLCMNLK-a7-b1 | AME1 | OKP1 | 231 | 274 | 544,049 | 4 | 0,6 | 6 | 6 | 32,79 |
| 43 | NEIQELKAGK-SKELK-a7-b2 | AME1 | OKP1 | 231 | 368 | 468,518 | 4 | -0,6 | 4 | 4 | 31,86 |
| 44 | NEIQELKAGK-QAKFPSR-a7-b3 | AME1 | OKP1 | 231 | 222 | 525,792 | 4 | -0,3 | 5 | 2 | 25,86 |
| 45 | AGKDWHDLQNEQAK-LTEKLIQK-a3-b4 | AME1 | OKP1 | 234 | 290 | 917,155 | 3 | -1,3 | 12 | 5 | 37,56 |
| 46 | AGKDWHDLQNEQAK-LCMNLKTNNK-a3-b6 | AME1 | OKP1 | 234 | 280 | 1004,828 | 3 | 0 | 13 | 5 | 35,14 |
| 47 | AGKDWHDLQNEQAK-RLTEKLIQK-a3-b5 | AME1 | OKP1 | 234 | 290 | 581,917 | 5 | 0,1 | 10 | 7 | 34,99 |
| 48 | AGKDWHDLQNEQAK-LTEKLIQK-a3-b4 | AME1 | OKP1 | 234 | 290 | 917,156 | 3 | 0,1 | 11 | 6 | 34,72 |
| 49 | AGKDWHDLQNEQAK-TNNKK-a3-b4 | AME1 | OKP1 | 234 | 284 | 794,4 | 3 | -0,2 | 6 | 8 | 34,31 |
| 50 | AGKDWHDLQNEQAK-TNNKK-a3-b4 | AME1 | OKP1 | 234 | 284 | 596,053 | 4 | 0,8 | 6 | 4 | 33,83 |
| 51 | AGKDWHDLQNEQAK-LCMNLKTNNKK-a3-b6 | AME1 | OKP1 | 234 | 280 | 1047,524 | 3 | -2 | 11 | 1 | 33,03 |
| 52 | AGKDWHDLQNEQAK-LCMNLKTNNK-a3-b6 | AME1 | OKP1 | 234 | 280 | 753,873 | 4 | 0,5 | 10 | 2 | 29,9 |
| 53 | AGKDWHDLQNEQAK-TNNKK-a3-b4 | AME1 | OKP1 | 234 | 284 | 596,052 | 4 | -0,1 | 5 | 1 | 29,71 |
| 54 | AGKDWHDLQNEQAK-IYSKK-a3-b4 | AME1 | OKP1 | 234 | 202 | 604,563 | 4 | -0,3 | 4 | 1 | 27,45 |
| 55 | AGKDWHDLQNEQAK-KLCMNLK-a3-b1 | AME1 | OKP1 | 234 | 274 | 671,589 | 4 | -0,4 | 8 | 2 | 25,48 |
| 56 | AGKDWHDLQNEQAK-LTEKLIQK-a3-b4 | AME1 | OKP1 | 234 | 290 | 550,695 | 5 | -2,8 | 8 | 2 | 22,8 |
| 57 | DWHDLQNEQAKLNDK-LTEKLIQK-a11-b4 | AME1 | OKP1 | 245 | 290 | 741,642 | 4 | -0,8 | 11 | 4 | 36,62 |
| 58 | DWHDLQNEQAKLNDK-LCMNLKTNNK-a11-b6 | AME1 | OKP1 | 245 | 280 | 807,396 | 4 | -0,6 | 16 | 2 | 35,3 |
| 59 | DWHDLQNEQAKLNDK-LTEKLIQK-a11-b4 | AME1 | OKP1 | 245 | 290 | 741,644 | 4 | 2 | 8 | 3 | 32,65 |
| 60 | DWHDLQNEQAKLNDK-RLTEKLIQK-a11-b5 | AME1 | OKP1 | 245 | 290 | 780,668 | 4 | 0,5 | 5 | 5 | 29,32 |
| 61 | DWHDLQNEQAKLNDK-LIQKDLHPVLNK-a11-b4 | AME1 | OKP1 | 245 | 294 | 852,952 | 4 | -2 | 10 | 7 | 28,3 |
| 62 | LNDKVK-TNNKK-a4-b4 | AME1 | OKP1 | 249 | 284 | 486,617 | 3 | 1,2 | 7 | 3 | 34,82 |
| 63 | LNDKVK-IYSKK-a4-b4 | AME1 | OKP1 | 249 | 202 | 373,725 | 4 | -0,8 | 5 | 4 | 31,53 |
| 64 | FIENLQTDLAKIYHCHVYMFK-NEIQELKAGK-a11-b7 | CHL4 | AME1 | 138 | 231 | 788,206 | 5 | -0,8 | 12 | 2 | 29,98 |
| 65 | LKFNGNDIFGGLHELCDK-LPLTSVILAGHTKIMR-a2-b13 | CHL4 | IML3 | 403 | 215 | 793,627 | 5 | -3,4 | 10 | 4 | 23,32 |
| 66 | YNDLKEFNEHVHNIR-TSQFEKPHYVLLK-a5-b6 | CHL4 | MCM21 | 349 | 218 | 609,985 | 6 | -0,4 | 9 | 1 | 24,16 |
| 67 | KNEDSGEPVYISR-TSQFEKPHYVLLK-a1-b6 | CHL4 | MCM21 | 363 | 218 | 805,919 | 4 | -0,4 | 4 | 1 | 22,65 |
| 68 | FQGKPLISR-IYSKK-a4-b4 | CHL4 | OKP1 | 192 | 202 | 456,022 | 4 | 0 | 6 | 2 | 37,99 |
| 69 | KYTPSELALYEIR-ATKASQEL-a1-b3 | CSE4 | H2A1 | 131 | 127 | 856,459 | 3 | 0,5 | 6 | 3 | 25,14 |
| 70 | ITIMKK-SAKATK-a5-b3 | CSE4 | H2A1 | 215 | 124 | 369,727 | 4 | -0,9 | 9 | 2 | 36,12 |
| 71 | RITIMKK-SAKATK-a6-b3 | CSE4 | H2A1 | 215 | 124 | 544,668 | 3 | 0,7 | 9 | 7 | 34,11 |
| 72 | RITIMKK-SAKATK-a6-b3 | CSE4 | H2A1 | 215 | 124 | 544,667 | 3 | -0,9 | 10 | 5 | 33,96 |
| 73 | KDMQLAR-SAKATK-a1-b3 | CSE4 | H2A1 | 216 | 124 | 535,3 | 3 | -0,1 | 7 | 1 | 36,43 |
| 74 | EVTDEFTTKDQDLR-KSTISAR-a9-b1 | CSE4 | H2B1 | 172 | 90 | 866,107 | 3 | -2,2 | 8 | 4 | 34,04 |
| 75 | EVTDEFTTKDQDLR-KSTISAR-a9-b1 | CSE4 | H2B1 | 172 | 111 | 866,107 | 3 | -2 | 8 | 2 | 33,85 |
| 76 | LVKEVTDEFTTK-GLGKGGAK-a3-b4 | CSE4 | H4 | 163 | 13 | 559,313 | 4 | 0,7 | 6 | 4 | 33,73 |
| 77 | LVKEVTDEFTTK-GGKGLGK-a3-b3 | CSE4 | H4 | 163 | 9 | 721,735 | 3 | -0,4 | 5 | 3 | 27,55 |
| 78 | LVKEVTDEFTTK-GLGKGGAK-a3-b4 | CSE4 | H4 | 163 | 13 | 559,312 | 4 | -1,9 | 4 | 4 | 27,51 |
| 79 | EVTDEFTTKDQDLR-GLGKGGAK-a9-b4 | CSE4 | H4 | 172 | 13 | 841,097 | 3 | -0,8 | 6 | 2 | 31,83 |
| 80 | EVTDEFTTKDQDLR-DSVTYTEHAKR-a9-b10 | CSE4 | H4 | 172 | 78 | 1047,506 | 3 | 0,3 | 11 | 7 | 31,15 |
| 81 | EVTDEFTTKDQDLR-GKGGK-a9-b2 | CSE4 | H4 | 172 | 6 | 760,718 | 3 | 0,8 | 4 | 5 | 30,1 |
| 82 | EVTDEFTTKDQDLR-DSVTYTEHAKR-a9-b10 | CSE4 | H4 | 172 | 78 | 1047,503 | 3 | -2,8 | 12 | 4 | 29,15 |
| 83 | EVTDEFTTKDQDLR-DSVTYTEHAKR-a9-b10 | CSE4 | H4 | 172 | 78 | 1047,506 | 3 | 0,2 | 10 | 3 | 27,71 |
| 84 | EVTDEFTTKDQDLR-RKTVTSLDVVYALK-a9-b2 | CSE4 | H4 | 172 | 80 | 686,166 | 5 | -1,1 | 10 | 2 | 25,22 |
| 85 | RITIMKK-GKGGK-a6-b2 | CSE4 | H4 | 215 | 6 | 368,98 | 4 | -0,4 | 8 | 4 | 35,43 |
| 86 | RITIMKK-GKGGK-a6-b2 | CSE4 | H4 | 215 | 6 | 368,98 | 4 | -0,9 | 7 | 4 | 31,84 |
| 87 | ITIMKK-GKGGK-a5-b2 | CSE4 | H4 | 215 | 6 | 329,955 | 4 | -1,9 | 4 | 1 | 30,21 |
| 88 | RITIMKK-GKGGK-a6-b2 | CSE4 | H4 | 215 | 6 | 368,98 | 4 | -2,5 | 6 | 2 | 29,5 |
| 89 | EVTDEFTTKDQDLR-SNKPVLDIDK-a9-b3 | CSE4 | MIF2 | 172 | 311 | 741,379 | 4 | 0,8 | 11 | 4 | 33,44 |
| 90 | EVTDEFTTKDQDLR-KSNKPVLDIDK-a9-b4 | CSE4 | MIF2 | 172 | 311 | 1030,866 | 3 | -1 | 10 | 8 | 31,96 |
| 91 | EVTDEFTTKDQDLR-KSNKPVLDIDK-a9-b1 | CSE4 | MIF2 | 172 | 308 | 1030,865 | 3 | -1,4 | 12 | 4 | 28,56 |
| 92 | EVTDEFTTKDQDLR-RKSNKPVLDIDK-a9-b5 | CSE4 | MIF2 | 172 | 311 | 812,429 | 4 | 1,6 | 7 | 2 | 26,55 |
| 93 | KYTPSELALYEIR-GSKQLR-a1-b3 | CSE4 | OKP1 | 131 | 95 | 602,836 | 4 | 1,5 | 6 | 1 | 31,36 |
| 94 | AKQLLATR-LLYKLDLR-a2-b4 | CTF19 | AME1 | 229 | 172 | 518,572 | 4 | 0,4 | 11 | 1 | 40,48 |
| 95 | TGIFQNLINLLKR-NEIQELKAGK-a12-b7 | CTF19 | AME1 | 266 | 231 | 699,907 | 4 | 1,4 | 10 | 2 | 33,67 |
| 96 | NFQKCLLSLYEFDKIK-LEKNMIKFK-a4-b3 | CTF19 | CHL4 | 239 | 328 | 667,567 | 5 | 1,4 | 7 | 13 | 22,59 |
| 97 | NFQKCLLSLYEFDK-VFLQLVEVQKR-a4-b10 | CTF19 | MCM21 | 239 | 280 | 1100,929 | 3 | -0,2 | 15 | 9 | 34,17 |
| 98 | NFQKCLLSLYEFDK-VFLQLVEVQKRR-a4-b10 | CTF19 | MCM21 | 239 | 280 | 692,18 | 5 | -1,4 | 13 | 3 | 32,9 |
| 99 | NFQKCLLSLYEFDK-VFLQLVEVQKRR-a4-b10 | CTF19 | MCM21 | 239 | 280 | 864,974 | 4 | 0,7 | 10 | 3 | 31,64 |
| 100 | TGLKEICNVCLFPDMYAR-IKSNSWFLFK-a4-b2 | CTF19 | MCM21 | 355 | 229 | 899,204 | 4 | -1,9 | 7 | 1 | 29,18 |
| 101 | TGIFQNLINLLKR-KVIQAEYR-a12-b1 | CTF19 | OKP1 | 266 | 132 | 669,143 | 4 | 0,4 | 9 | 2 | 34,91 |
| 102 | LDNLQDIKK-LLYKLDLR-a8-b4 | DSN1 | AME1 | 99 | 172 | 565,085 | 4 | -1 | 11 | 2 | 39,05 |
| 103 | SLHKLELK-KTGIDVK-a4-b1 | DSN1 | MIF2 | 303 | 12 | 467,033 | 4 | -2 | 10 | 2 | 35,59 |
| 104 | KLLPNSKNVENTK-LGLKSR-a7-b4 | DSN1 | MIF2 | 436 | 9 | 574,591 | 4 | 0,9 | 7 | 2 | 25,17 |
| 105 | ISSETDDDHSQVINPQQLLKGLSLSFSKK-LGLLGDKEDEK-a20-b7 | DSN1 | MTW1 | 564 | 254 | 914,478 | 5 | -0,6 | 6 | 1 | 21,49 |
| 106 | NQEEEGELEHLTKK-KEYDDDAVRR-a13-b1 | DSN1 | NSL1 | 322 | 38 | 515,421 | 6 | -0,4 | 8 | 4 | 24,65 |
| 107 | SKLESTKAETDYVDPK-KEYDDDAVRR-a7-b1 | DSN1 | NSL1 | 330 | 38 | 643,721 | 5 | 0 | 7 | 5 | 29,12 |
| 108 | LESTKAETDYVDPKR-KEYDDDAVRR-a5-b1 | DSN1 | NSL1 | 330 | 38 | 526,764 | 6 | -1,6 | 6 | 2 | 20,23 |
| 109 | KLLPNSKNVENTK-TVKQLIMESQEK-a7-b3 | DSN1 | NSL1 | 436 | 81 | 764,672 | 4 | -2,1 | 5 | 3 | 23,68 |
| 110 | GLSLSFSKK-YNVEKVK-a8-b5 | DSN1 | NSL1 | 572 | 205 | 661,71 | 3 | -1,4 | 7 | 4 | 29,22 |
| 111 | AGSAAKASQSR-RVEKK-a6-b4 | H2A1 | CSE4 | 14 | 130 | 610,679 | 3 | 1,6 | 6 | 1 | 30,99 |
| 112 | LLGNVTIAQGGVLPNIHQNLLPKK-KYTPSELALYEIR-a23-b1 | H2A1 | CSE4 | 120 | 131 | 1065,11 | 4 | 3,6 | 9 | 2 | 26,3 |
| 113 | SAKATK-KQSLK-a3-b1 | H2A1 | CSE4 | 124 | 122 | 449,273 | 3 | -2 | 7 | 3 | 33,35 |
| 114 | ATKASQEL-KQSLK-a3-b1 | H2A1 | CSE4 | 127 | 122 | 529,971 | 3 | 0,1 | 5 | 1 | 26,23 |
| 115 | ATKASQEL-ATKNLFPR-a3-b3 | H2A1 | CSE4 | 127 | 49 | 644,359 | 3 | 0,5 | 7 | 2 | 25,65 |
| 116 | ATKASQEL-ITIMKK-a3-b5 | H2A1 | CSE4 | 127 | 215 | 573,332 | 3 | 1,1 | 5 | 2 | 23,58 |
| 117 | GGKAGSAAK-KPASK-a3-b1 | H2A1 | H2B1 | 8 | 29 | 471,941 | 3 | -0,2 | 7 | 4 | 35,77 |
| 118 | AGSAAKASQSR-APAEKKPAAK-a6-b5 | H2A1 | H2B1 | 14 | 38 | 727,739 | 3 | 1 | 14 | 3 | 41,24 |
| 119 | AGSAAKASQSR-KPAAK-a6-b1 | H2A1 | H2B1 | 14 | 39 | 562,316 | 3 | -0,5 | 8 | 3 | 40,83 |
| 120 | AGSAAKASQSR-KPASKAPAEK-a6-b5 | H2A1 | H2B1 | 14 | 33 | 733,07 | 3 | -0,3 | 14 | 4 | 39,71 |
| 121 | AGSAAKASQSR-KPASK-a6-b1 | H2A1 | H2B1 | 14 | 29 | 567,648 | 3 | 0,1 | 8 | 2 | 37,31 |
| 122 | AGSAAKASQSR-APAEKKPAAK-a6-b5 | H2A1 | H2B1 | 14 | 17 | 727,74 | 3 | 3 | 11 | 3 | 37,06 |
| 123 | AGSAAKASQSR-KPASKAPAEK-a6-b5 | H2A1 | H2B1 | 14 | 12 | 733,07 | 3 | -0,3 | 11 | 5 | 36,84 |
| 124 | AGSAAKASQSR-KPASK-a6-b1 | H2A1 | H2B1 | 14 | 8 | 567,649 | 3 | 0,3 | 7 | 2 | 36,37 |
| 125 | AGSAAKASQSR-KPASKAPAEK-a6-b5 | H2A1 | H2B1 | 14 | 12 | 733,07 | 3 | -0,1 | 12 | 2 | 34,08 |
| 126 | AGSAAKASQSR-AVTKYSSSTQA-a6-b4 | H2A1 | H2B1 | 14 | 145 | 771,729 | 3 | 0,5 | 11 | 8 | 33,99 |
| 127 | AGSAAKASQSR-APAEKKPAAK-a6-b5 | H2A1 | H2B1 | 14 | 17 | 727,738 | 3 | -0,2 | 10 | 2 | 33,57 |
| 128 | AGSAAKASQSR-AVTKYSSSTQA-a6-b4 | H2A1 | H2B1 | 14 | 124 | 771,728 | 3 | -0,2 | 9 | 3 | 32,81 |
| 129 | AGSAAKASQSR-AVTKYSSSTQA-a6-b4 | H2A1 | H2B1 | 14 | 124 | 771,727 | 3 | -0,9 | 11 | 2 | 29,63 |
| 130 | SAKAGLTFPVGR-AVTKYSSSTQA-a3-b4 | H2A1 | H2B1 | 22 | 145 | 828,443 | 3 | 0 | 10 | 4 | 31,14 |
| 131 | SAKAGLTFPVGR-AVTKYSSSTQA-a3-b4 | H2A1 | H2B1 | 22 | 124 | 828,443 | 3 | 0 | 8 | 2 | 29,08 |
| 132 | LLGNVTIAQGGVLPNIHQNLLPKK-LILPGELAKHAVSEGTR-a23-b9 | H2A1 | H2B1 | 120 | 133 | 745,1 | 6 | -0,7 | 9 | 2 | 30,42 |
| 133 | SAKATK-IVYKR-a3-b4 | H2A1 | MIF2 | 124 | 306 | 355,969 | 4 | -1,1 | 7 | 2 | 38,92 |
| 134 | ATKASQEL-IVYKR-a3-b4 | H2A1 | MIF2 | 127 | 306 | 554,986 | 3 | 0,2 | 5 | 2 | 32,97 |
| 135 | ATKASQEL-IVYKR-a3-b4 | H2A1 | MIF2 | 127 | 306 | 554,986 | 3 | -1,1 | 4 | 2 | 22,66 |
| 136 | GGKAGSAAK-IYSKK-a3-b4 | H2A1 | OKP1 | 8 | 202 | 381,222 | 4 | 0,3 | 5 | 2 | 32,83 |
| 137 | AGSAAKASQSR-RIYSKK-a6-b5 | H2A1 | OKP1 | 14 | 202 | 655,703 | 3 | 2,6 | 5 | 1 | 30,96 |
| 138 | AGSAAKASQSRSAK-NYELKHWK-a6-b5 | H2A1 | OKP1 | 14 | 149 | 515,674 | 5 | -2,5 | 6 | 4 | 22,34 |
| 139 | QTHPDTGISQKSMSILNSFVNDIFER-STDLLISKIPFAR-a11-b8 | H2B1 | CSE4 | 61 | 155 | 913,276 | 5 | -2,3 | 10 | 4 | 24,17 |
| 140 | LILPGELAKHAVSEGTR-EVTDEFTTKDQDLR-a9-b9 | H2B1 | CSE4 | 112 | 172 | 906,973 | 4 | -2,6 | 13 | 3 | 33,78 |
| 141 | LILPGELAKHAVSEGTR-EVTDEFTTKDQDLR-a9-b9 | H2B1 | CSE4 | 112 | 172 | 906,974 | 4 | -1,1 | 9 | 7 | 26,77 |
| 142 | AVTKYSSSTQA-ATKNLFPR-a4-b3 | H2B1 | CSE4 | 124 | 49 | 742,729 | 3 | -2,5 | 7 | 2 | 26,13 |
| 143 | LILPGELAKHAVSEGTR-EVTDEFTTKDQDLR-a9-b9 | H2B1 | CSE4 | 133 | 172 | 906,974 | 4 | -0,6 | 14 | 10 | 34,33 |
| 144 | LILPGELAKHAVSEGTR-ATKNLFPR-a9-b3 | H2B1 | CSE4 | 133 | 49 | 575,73 | 5 | 0,1 | 7 | 8 | 34,09 |
| 145 | AVTKYSSSTQA-ATKNLFPR-a4-b3 | H2B1 | CSE4 | 145 | 49 | 742,731 | 3 | 0,5 | 6 | 4 | 29,94 |
| 146 | KPASKAPAEK-GGKAGSAAK-a5-b3 | H2B1 | H2A1 | 12 | 8 | 637,363 | 3 | 1,2 | 12 | 3 | 40,5 |
| 147 | KPASKAPAEK-GGKAGSAAK-a5-b3 | H2B1 | H2A1 | 33 | 8 | 637,363 | 3 | 0,7 | 12 | 6 | 40,22 |
| 148 | APAEKKPAAK-GGKAGSAAK-a6-b3 | H2B1 | H2A1 | 39 | 8 | 474,275 | 4 | -0,4 | 9 | 3 | 36,36 |
| 149 | ETYSSYIYKVLK-AGSAAKASQSR-a9-b6 | H2B1 | H2A1 | 47 | 14 | 888,801 | 3 | -0,2 | 7 | 2 | 25,57 |
| 150 | VLKQTHPDTGISQK-AGSAAKASQSR-a3-b6 | H2B1 | H2A1 | 50 | 14 | 681,368 | 4 | 0 | 12 | 3 | 34,86 |
| 151 | VLKQTHPDTGISQK-GGKAGSAAK-a3-b3 | H2B1 | H2A1 | 50 | 8 | 609,587 | 4 | 0,1 | 10 | 6 | 33,92 |
| 152 | ETYSSYIYKVLK-GGKAGSAAK-a9-b3 | H2B1 | H2A1 | 68 | 8 | 595,072 | 4 | -0,7 | 8 | 3 | 28,61 |
| 153 | ETYSSYIYKVLK-AGSAAKASQSR-a9-b6 | H2B1 | H2A1 | 68 | 14 | 888,801 | 3 | -0,4 | 9 | 2 | 26,88 |
| 154 | VLKQTHPDTGISQK-GGKAGSAAK-a3-b3 | H2B1 | H2A1 | 71 | 8 | 609,588 | 4 | 1,2 | 8 | 4 | 34,27 |
| 155 | VLKQTHPDTGISQK-AGSAAKASQSR-a3-b6 | H2B1 | H2A1 | 71 | 14 | 681,369 | 4 | 0,7 | 9 | 2 | 31,6 |
| 156 | LILPGELAKHAVSEGTR-ATKASQEL-a9-b3 | H2B1 | H2A1 | 112 | 127 | 694,636 | 4 | -1,5 | 5 | 2 | 27,34 |
| 157 | AVTKYSSSTQA-GGKAGSAAK-a4-b3 | H2B1 | H2A1 | 124 | 8 | 676,02 | 3 | -0,1 | 8 | 3 | 34,82 |
| 158 | AVTKYSSSTQA-GGKAGSAAK-a4-b3 | H2B1 | H2A1 | 124 | 8 | 676,021 | 3 | 0,4 | 9 | 4 | 32,95 |
| 159 | LILPGELAKHAVSEGTR-SAKATK-a9-b3 | H2B1 | H2A1 | 133 | 124 | 507,494 | 5 | 1,8 | 5 | 6 | 37,86 |
| 160 | LILPGELAKHAVSEGTR-ATKASQEL-a9-b3 | H2B1 | H2A1 | 133 | 127 | 694,638 | 4 | 1 | 9 | 6 | 35,76 |
| 161 | LILPGELAKHAVSEGTR-AGSAAKASQSR-a9-b6 | H2B1 | H2A1 | 133 | 14 | 741,16 | 4 | 1,9 | 9 | 2 | 26,27 |
| 162 | AVTKYSSSTQA-GGKAGSAAK-a4-b3 | H2B1 | H2A1 | 145 | 8 | 676,021 | 3 | 0,3 | 8 | 5 | 32,92 |
| 163 | KPASKAPAEK-GGKGLGK-a5-b3 | H2B1 | H4 | 12 | 9 | 445,764 | 4 | 0,6 | 10 | 2 | 35,93 |
| 164 | KPASKAPAEK-GGKGLGK-a5-b3 | H2B1 | H4 | 33 | 9 | 594,017 | 3 | 1,7 | 9 | 4 | 36 |
| 165 | KPASKAPAEK-GKGGK-a5-b2 | H2B1 | H4 | 33 | 6 | 403,238 | 4 | -0,1 | 4 | 2 | 33,97 |
| 166 | IATEASKLAAYNK-TVTSLDVVYALKR-a7-b12 | H2B1 | H4 | 104 | 92 | 746,17 | 4 | 0,6 | 7 | 4 | 25,38 |
| 167 | LILPGELAKHAVSEGTR-GLGKGGAK-a9-b4 | H2B1 | H4 | 112 | 13 | 654,629 | 4 | 1,9 | 4 | 2 | 27,38 |
| 168 | LILPGELAKHAVSEGTR-GLGKGGAK-a9-b4 | H2B1 | H4 | 133 | 13 | 523,905 | 5 | 1,8 | 8 | 5 | 33,5 |
| 169 | AVTKYSSSTQA-GKGGK-a4-b2 | H2B1 | H4 | 145 | 6 | 575,974 | 3 | 2 | 6 | 2 | 30,24 |
| 170 | LILPGELAKHAVSEGTR-SNKPVLDIDK-a9-b3 | H2B1 | MIF2 | 112 | 311 | 612,146 | 5 | -0,9 | 8 | 14 | 32,15 |
| 171 | LILPGELAKHAVSEGTR-KSNKPVLDIDK-a9-b4 | H2B1 | MIF2 | 112 | 311 | 637,764 | 5 | -1,7 | 6 | 5 | 30,78 |
| 172 | LILPGELAKHAVSEGTR-SNKPVLDIDK-a9-b3 | H2B1 | MIF2 | 133 | 311 | 612,147 | 5 | 1 | 12 | 11 | 36,92 |
| 173 | LILPGELAKHAVSEGTR-RKSNKPVLDIDK-a9-b5 | H2B1 | MIF2 | 133 | 311 | 668,985 | 5 | -0,5 | 9 | 4 | 35,8 |
| 174 | LILPGELAKHAVSEGTR-VKVAPLQYWR-a9-b2 | H2B1 | MIF2 | 133 | 291 | 638,366 | 5 | -0,8 | 12 | 8 | 33,89 |
| 175 | LILPGELAKHAVSEGTR-KSNKPVLDIDK-a9-b4 | H2B1 | MIF2 | 133 | 311 | 637,766 | 5 | 1,4 | 5 | 4 | 31,66 |
| 176 | LILPGELAKHAVSEGTR-KSNKPVLDIDK-a9-b1 | H2B1 | MIF2 | 133 | 308 | 1062,273 | 3 | 2,9 | 9 | 1 | 27,85 |
| 177 | AVTKYSSSTQA-KTGIDVK-a4-b1 | H2B1 | MIF2 | 145 | 12 | 680,701 | 3 | 0,1 | 6 | 2 | 27,32 |
| 178 | AVTKYSSSTQA-RKYSTR-a4-b2 | H2B1 | MIF2 | 145 | 148 | 697,368 | 3 | 0,2 | 4 | 1 | 25,6 |
| 179 | AVTKYSSSTQA-SNKPVLDIDK-a4-b3 | H2B1 | MIF2 | 145 | 311 | 803,425 | 3 | 1,9 | 7 | 3 | 25,52 |
| 180 | APAEKKPAAK-RIYSKK-a6-b5 | H2B1 | OKP1 | 18 | 202 | 486,293 | 4 | -0,4 | 4 | 1 | 25,98 |
| 181 | KPASKAPAEK-NYELKHWK-a5-b5 | H2B1 | OKP1 | 33 | 149 | 571,066 | 4 | 3 | 8 | 2 | 29,17 |
| 182 | APAEKKPAAK-NYELKHWK-a5-b5 | H2B1 | OKP1 | 38 | 149 | 567,067 | 4 | 1,5 | 8 | 1 | 29 |
| 183 | DSVTYTEHAKR-RVEKK-a10-b4 | H4 | CSE4 | 78 | 130 | 526,536 | 4 | 1,2 | 4 | 1 | 31,37 |
| 184 | DSVTYTEHAKR-KSTISAR-a10-b1 | H4 | H2B1 | 78 | 111 | 552,293 | 4 | 1,4 | 9 | 8 | 34,53 |
| 185 | SFLESVIRDSVTYTEHAKR-KSTISAR-a18-b1 | H4 | H2B1 | 78 | 90 | 628,337 | 5 | -1,1 | 7 | 5 | 31,89 |
| 186 | DSVTYTEHAKR-KSTISAR-a10-b1 | H4 | H2B1 | 78 | 90 | 552,292 | 4 | -1,4 | 9 | 6 | 31,62 |
| 187 | DSVTYTEHAKRK-KSTISAR-a10-b1 | H4 | H2B1 | 78 | 90 | 584,316 | 4 | -0,8 | 8 | 12 | 31,36 |
| 188 | DSVTYTEHAKRK-KPAAKK-a10-b5 | H4 | H2B1 | 78 | 43 | 554,312 | 4 | -0,9 | 6 | 3 | 30,93 |
| 189 | DSVTYTEHAKR-KPAAKK-a10-b5 | H4 | H2B1 | 78 | 22 | 522,287 | 4 | -2 | 5 | 3 | 30,6 |
| 190 | DSVTYTEHAKRK-KSTISAR-a10-b1 | H4 | H2B1 | 78 | 111 | 584,316 | 4 | -0,7 | 9 | 4 | 29,74 |
| 191 | DSVTYTEHAKR-KSTISAR-a10-b1 | H4 | H2B1 | 78 | 90 | 552,292 | 4 | -0,2 | 6 | 5 | 28,79 |
| 192 | DSVTYTEHAKR-LAAYNKK-a10-b6 | H4 | H2B1 | 78 | 110 | 751,064 | 3 | 2,3 | 7 | 4 | 26,29 |
| 193 | DSVTYTEHAKR-KPAAKK-a10-b5 | H4 | H2B1 | 78 | 22 | 522,288 | 4 | -0,7 | 4 | 3 | 25,58 |
| 194 | DSVTYTEHAKRK-KSTISAR-a10-b1 | H4 | H2B1 | 78 | 90 | 584,316 | 4 | -0,8 | 6 | 4 | 24,39 |
| 195 | TVTSLDVVYALKR-KETYSSYIYK-a12-b1 | H4 | H2B1 | 92 | 59 | 721,641 | 4 | 0,2 | 6 | 4 | 27,4 |
| 196 | TVTSLDVVYALKR-KETYSSYIYK-a12-b1 | H4 | H2B1 | 92 | 38 | 721,642 | 4 | 1,2 | 6 | 1 | 23,42 |
| 197 | AVLKSFLESVIR-IVYKR-a4-b4 | H4 | MIF2 | 60 | 306 | 545,082 | 4 | -0,4 | 7 | 3 | 36,15 |
| 198 | AVLKSFLESVIR-IVYKR-a4-b4 | H4 | MIF2 | 60 | 306 | 545,083 | 4 | 0,2 | 5 | 4 | 33,39 |
| 199 | DSVTYTEHAKRK-SNKPVLDIDK-a10-b3 | H4 | MIF2 | 78 | 311 | 540,89 | 5 | -0,4 | 12 | 5 | 36,24 |
| 200 | DSVTYTEHAKR-SNKPVLDIDK-a10-b3 | H4 | MIF2 | 78 | 311 | 515,272 | 5 | 1,3 | 10 | 6 | 33,94 |
| 201 | DSVTYTEHAKR-SNKPVLDIDK-a10-b3 | H4 | MIF2 | 78 | 311 | 515,271 | 5 | -1,2 | 9 | 6 | 30,84 |
| 202 | DSVTYTEHAKRK-SNKPVLDIDK-a10-b3 | H4 | MIF2 | 78 | 311 | 540,89 | 5 | -0,9 | 9 | 4 | 29,75 |
| 203 | LPLTSVILAGHTKIMR-EQVAKGGLL-a13-b5 | IML3 | CHL4 | 215 | 454 | 934,548 | 3 | -2 | 14 | 2 | 27,68 |
| 204 | TSQFEKPHYVLLK-LLYKLDLR-a6-b4 | MCM21 | AME1 | 218 | 172 | 552,92 | 5 | -1,1 | 11 | 4 | 37,37 |
| 205 | IKSNSWFLFK-NEIQELKAGK-a2-b7 | MCM21 | AME1 | 229 | 231 | 846,132 | 3 | -0,7 | 9 | 3 | 30,42 |
| 206 | AQADIPATPIPYEPKKR-AKQLLATR-a15-b2 | MCM21 | CTF19 | 95 | 229 | 587,337 | 5 | -2,4 | 11 | 2 | 33,2 |
| 207 | AQADIPATPIPYEPKKR-TGIFQNLINLLKR-a15-b12 | MCM21 | CTF19 | 95 | 266 | 713,209 | 5 | -0,9 | 11 | 4 | 30,58 |
| 208 | AQADIPATPIPYEPKK-LQEEKDALLTR-a15-b5 | MCM21 | CTF19 | 95 | 61 | 798,683 | 4 | -3,5 | 8 | 1 | 28,24 |
| 209 | AQADIPATPIPYEPKKR-TGIFQNLINLLKR-a15-b12 | MCM21 | CTF19 | 95 | 266 | 713,21 | 5 | 0,4 | 7 | 3 | 24,88 |
| 210 | AQADIPATPIPYEPKK-TGIFQNLINLLKR-a15-b12 | MCM21 | CTF19 | 95 | 266 | 852,234 | 4 | -1 | 7 | 1 | 23,37 |
| 211 | AKLENEEILPEQEWVLK-TGIFQNLINLLKR-a2-b12 | MCM21 | CTF19 | 99 | 266 | 934,523 | 4 | -1 | 9 | 3 | 27,53 |
| 212 | IKSNSWFLFK-AKQLLATR-a2-b2 | MCM21 | CTF19 | 229 | 229 | 577,585 | 4 | -3,5 | 5 | 1 | 25,16 |
| 213 | QIFKDLEAK-ENNSKTK-a4-b5 | MCM21 | CTF19 | 286 | 89 | 513,027 | 4 | -0,8 | 6 | 1 | 28,47 |
| 214 | GRPKKDPNAK-WLPSKALR-a5-b5 | MIF2 | CHL4 | 364 | 110 | 555,327 | 4 | 0,4 | 4 | 1 | 24,66 |
| 215 | GRPKKDPNAK-SPLHDYKK-a5-b7 | MIF2 | CHL4 | 364 | 287 | 447,851 | 5 | -1,1 | 6 | 1 | 20,78 |
| 216 | IESGGIENGEWLKHGILEANVK-KNEDSGEPVYISR-a13-b1 | MIF2 | CHL4 | 397 | 363 | 805,611 | 5 | -1,2 | 9 | 4 | 23,06 |
| 217 | NEKIVYK-ITIMKK-a3-b5 | MIF2 | CSE4 | 302 | 215 | 588,683 | 3 | -1 | 8 | 4 | 34,74 |
| 218 | NEKIVYKR-RITIMKK-a3-b6 | MIF2 | CSE4 | 302 | 215 | 416,053 | 5 | -2 | 9 | 1 | 34,34 |
| 219 | NEKIVYK-ITIMKK-a3-b5 | MIF2 | CSE4 | 302 | 215 | 441,764 | 4 | -1,8 | 7 | 4 | 33,93 |
| 220 | NEKIVYKR-ITIMKK-a3-b5 | MIF2 | CSE4 | 302 | 215 | 384,833 | 5 | -1,9 | 7 | 4 | 28,26 |
| 221 | NEKIVYK-RITIMKK-a3-b6 | MIF2 | CSE4 | 302 | 215 | 640,719 | 3 | 3,1 | 5 | 4 | 25,83 |
| 222 | NEKIVYKR-ITIMKK-a3-b5 | MIF2 | CSE4 | 302 | 215 | 480,79 | 4 | 0,7 | 6 | 1 | 25,58 |
| 223 | NEKIVYKR-ITIMKK-a7-b5 | MIF2 | CSE4 | 306 | 215 | 480,789 | 4 | -0,9 | 6 | 1 | 26,28 |
| 224 | NEKIVYKR-ITIMKK-a7-b5 | MIF2 | CSE4 | 306 | 215 | 480,79 | 4 | 0,4 | 7 | 1 | 26,2 |
| 225 | RKSNKPVLDIDK-ITIMKK-a2-b5 | MIF2 | CSE4 | 308 | 215 | 457,475 | 5 | -0,9 | 4 | 1 | 26,15 |
| 226 | KSNKPVLDIDK-ITIMKK-a4-b5 | MIF2 | CSE4 | 311 | 215 | 426,256 | 5 | 0,5 | 9 | 4 | 36,82 |
| 227 | RKSNKPVLDIDK-ITIMKK-a5-b5 | MIF2 | CSE4 | 311 | 215 | 457,475 | 5 | -1,8 | 5 | 1 | 23,56 |
| 228 | GRPKKDPNAK-KQSLK-a4-b1 | MIF2 | CSE4 | 363 | 122 | 617,699 | 3 | -0,6 | 7 | 2 | 34 |
| 229 | RKSNKPVLDIDK-KSAKATK-a2-b1 | MIF2 | H2A1 | 308 | 121 | 571,591 | 4 | 0,9 | 4 | 1 | 21,84 |
| 230 | RKSNKPVLDIDK-SAKATK-a5-b3 | MIF2 | H2A1 | 311 | 124 | 431,854 | 5 | -1,8 | 10 | 2 | 31,86 |
| 231 | RKSNKPVLDIDK-ATKASQEL-a5-b3 | MIF2 | H2A1 | 311 | 127 | 600,089 | 4 | -1,2 | 8 | 1 | 25,05 |
| 232 | KKPTPTRPYNYVPTGRPR-ATKASQEL-a1-b3 | MIF2 | H2A1 | 342 | 127 | 623,344 | 5 | -0,7 | 7 | 2 | 21,93 |
| 233 | KKPTPTRPYNYVPTGRPR-SAKATK-a2-b3 | MIF2 | H2A1 | 343 | 124 | 479,272 | 6 | -2 | 7 | 1 | 26,41 |
| 234 | RKSNKPVLDIDK-GKGGK-a2-b2 | MIF2 | H4 | 308 | 6 | 400,037 | 5 | -0,9 | 6 | 2 | 32,93 |
| 235 | SNKPVLDIDK-GLGKGGAK-a3-b4 | MIF2 | H4 | 311 | 13 | 489,03 | 4 | -2 | 8 | 2 | 32,63 |
| 236 | RKSNKPVLDIDK-GKGGK-a5-b2 | MIF2 | H4 | 311 | 6 | 400,037 | 5 | -1,9 | 5 | 1 | 32 |
| 237 | GRPKKDPNAK-GLGKGGAK-a5-b4 | MIF2 | H4 | 364 | 13 | 484,533 | 4 | -2 | 9 | 2 | 33,08 |
| 238 | RKYSTR-IYSKK-a2-b4 | MIF2 | OKP1 | 148 | 202 | 397,231 | 4 | -2,8 | 4 | 2 | 29,18 |
| 239 | GRPKKDPNAK-IYSKK-a5-b4 | MIF2 | OKP1 | 364 | 202 | 472,277 | 4 | -1,9 | 5 | 3 | 24,83 |
| 240 | LESLLENSVDKNFDKLELYVLR-SLHKLELK-a11-b4 | MTW1 | DSN1 | 78 | 303 | 749,221 | 5 | 0,8 | 4 | 1 | 23,24 |
| 241 | SGVAKLESLLENSVDK-ALDQSISKLQSWDK-a5-b8 | MTW1 | NNF1 | 67 | 26 | 861,957 | 4 | -1,5 | 10 | 2 | 26,39 |
| 242 | KLGLLGDKEDEK-VRYNVEKVK-a1-b7 | MTW1 | NSL1 | 247 | 205 | 436,916 | 6 | -1,2 | 15 | 13 | 39,14 |
| 243 | KLGLLGDKEDEK-YNVEKVK-a1-b5 | MTW1 | NSL1 | 247 | 205 | 473,065 | 5 | -0,1 | 10 | 14 | 38,3 |
| 244 | LGLLGDKEDEK-YNVEKVK-a7-b5 | MTW1 | NSL1 | 254 | 205 | 559,054 | 4 | -2 | 6 | 1 | 31,81 |
| 245 | KLGLLGDKEDEK-YNVEKVK-a8-b5 | MTW1 | NSL1 | 254 | 205 | 591,078 | 4 | -1,9 | 8 | 9 | 30,08 |
| 246 | LGLLGDKEDEK-VRYNVEKVK-a7-b7 | MTW1 | NSL1 | 254 | 205 | 498,479 | 5 | -1,1 | 6 | 1 | 27,55 |
| 247 | LGLLGDKEDEKQSAKPDAR-YNVEKVK-a7-b5 | MTW1 | NSL1 | 254 | 205 | 618,133 | 5 | -1,2 | 7 | 1 | 22,38 |
| 248 | LGLLGDKEDEKQSAKPDAR-YNVEKVK-a11-b5 | MTW1 | NSL1 | 258 | 205 | 515,279 | 6 | -0,9 | 8 | 3 | 32,53 |
| 249 | LGLLGDKEDEKQSAKPDAR-VRYNVEKVK-a11-b7 | MTW1 | NSL1 | 258 | 205 | 557,806 | 6 | -2,2 | 4 | 2 | 22,5 |
| 250 | LGLLGDKEDEKQSAKPDAR-YNVEKVK-a15-b5 | MTW1 | NSL1 | 262 | 205 | 515,279 | 6 | -0,8 | 10 | 2 | 29,74 |
| 251 | LNELDELILEAKER-LDNLQDIKK-a12-b8 | NNF1 | DSN1 | 93 | 99 | 727,903 | 4 | -0,2 | 10 | 2 | 33,22 |
| 252 | ALDQSISKLQSWDK-NFDKLELYVLR-a8-b4 | NNF1 | MTW1 | 26 | 82 | 792,177 | 4 | -0,1 | 14 | 1 | 39,63 |
| 253 | LQSWDKVSSCFPQYVNSK-SGVAKLESLLENSVDK-a6-b5 | NNF1 | MTW1 | 32 | 67 | 1333,671 | 3 | -2,2 | 9 | 3 | 29,27 |
| 254 | VNEMNDQLAQELKDLETQVEVEK-KNEMLLK-a13-b1 | NNF1 | MTW1 | 153 | 140 | 929,476 | 4 | -0,8 | 4 | 2 | 27,1 |
| 255 | DLETQVEVEKNEIGK-RVTKVK-a10-b4 | NNF1 | MTW1 | 163 | 150 | 650,365 | 4 | -0,2 | 5 | 5 | 33,18 |
| 256 | ALDQSISKLQSWDK-TVKQLIMESQEK-a8-b3 | NNF1 | NSL1 | 26 | 81 | 1063,896 | 3 | -0,1 | 14 | 3 | 33,27 |
| 257 | LQSWDKVSSCFPQYVNSK-TVKQLIMESQEK-a6-b3 | NNF1 | NSL1 | 32 | 81 | 936,72 | 4 | -2,7 | 8 | 2 | 22,06 |
| 258 | TDLHLPKK-KLLPNSK-a7-b1 | NSL1 | DSN1 | 37 | 430 | 472,786 | 4 | -3,4 | 7 | 2 | 25,7 |
| 259 | KEYDDDAVRR-SKLESTK-a1-b2 | NSL1 | DSN1 | 38 | 325 | 440,028 | 5 | -1,6 | 9 | 1 | 30,09 |
| 260 | TVKQLIMESQEK-KALPK-a3-b1 | NSL1 | DSN1 | 81 | 422 | 709,741 | 3 | -1,2 | 5 | 3 | 31,89 |
| 261 | TVKQLIMESQEK-ALPKEPK-a3-b4 | NSL1 | DSN1 | 81 | 426 | 589,082 | 4 | -1 | 5 | 2 | 29,74 |
| 262 | TVKQLIMESQEK-KTGIDVK-a3-b1 | NSL1 | MIF2 | 81 | 12 | 583,576 | 4 | -1,5 | 4 | 1 | 22,12 |
| 263 | SSSESDSSILMNVNDIKTLR-LLYKLDLR-a17-b4 | OKP1 | AME1 | 46 | 172 | 842,451 | 4 | -0,3 | 5 | 2 | 28,11 |
| 264 | LDVAPEAKSTQSK-LLYKLDLR-a8-b4 | OKP1 | AME1 | 57 | 172 | 636,863 | 4 | -0,4 | 10 | 5 | 40,59 |
| 265 | LDVAPEAKSTQSK-LLYKLDLR-a8-b4 | OKP1 | AME1 | 57 | 172 | 636,862 | 4 | -1,8 | 10 | 4 | 39,85 |
| 266 | LDVAPEAKSTQSK-DFNKSDDDQFRK-a8-b4 | OKP1 | AME1 | 57 | 160 | 757,125 | 4 | -0,3 | 7 | 4 | 33,93 |
| 267 | TNKEEGQYHHK-LLYKLDLR-a3-b4 | OKP1 | AME1 | 84 | 172 | 636,092 | 4 | -0,9 | 9 | 6 | 33,54 |
| 268 | TNKEEGQYHHK-KLLYK-a3-b1 | OKP1 | AME1 | 84 | 168 | 724,721 | 3 | 0,4 | 6 | 6 | 32,42 |
| 269 | TNKEEGQYHHK-LLYKLDLR-a3-b4 | OKP1 | AME1 | 84 | 172 | 847,788 | 3 | 1 | 9 | 4 | 32,19 |
| 270 | TNKEEGQYHHK-LNDKVK-a3-b4 | OKP1 | AME1 | 84 | 249 | 556,789 | 4 | -1,2 | 7 | 2 | 30,9 |
| 271 | TNKEEGQYHHK-LLYKLDLR-a3-b4 | OKP1 | AME1 | 84 | 172 | 509,075 | 5 | -0,6 | 6 | 3 | 26,76 |
| 272 | TNKEEGQYHHKGSK-LLYKLDLR-a11-b4 | OKP1 | AME1 | 92 | 172 | 704,13 | 4 | 1,2 | 12 | 14 | 34 |
| 273 | TNKEEGQYHHKGSK-DFNKSDDDQFRK-a11-b4 | OKP1 | AME1 | 92 | 160 | 824,391 | 4 | -0,5 | 12 | 10 | 33,81 |
| 274 | TNKEEGQYHHKGSK-LLYKLDLR-a11-b4 | OKP1 | AME1 | 92 | 172 | 704,13 | 4 | 1 | 11 | 5 | 32,67 |
| 275 | TNKEEGQYHHKGSK-KLLYK-a11-b1 | OKP1 | AME1 | 92 | 168 | 489,665 | 5 | -0,1 | 8 | 2 | 31,05 |
| 276 | TNKEEGQYHHKGSK-LNDKVK-a11-b4 | OKP1 | AME1 | 92 | 249 | 500,064 | 5 | 1,1 | 7 | 3 | 30,95 |
| 277 | TNKEEGQYHHKGSK-LLYKLDLR-a11-b4 | OKP1 | AME1 | 92 | 172 | 469,755 | 6 | -2,4 | 8 | 5 | 30,28 |
| 278 | TNKEEGQYHHKGSK-DFNKSDDDQFRK-a11-b4 | OKP1 | AME1 | 92 | 160 | 659,713 | 5 | -2,1 | 9 | 3 | 25,76 |
| 279 | TNKEEGQYHHKGSK-NEIQELKAGK-a11-b7 | OKP1 | AME1 | 92 | 231 | 728,127 | 4 | 3,1 | 7 | 3 | 25,23 |
| 280 | GSKQLR-LNDKVK-a3-b4 | OKP1 | AME1 | 95 | 249 | 514,639 | 3 | -1 | 7 | 1 | 30,58 |
| 281 | FEVGKESTGK-LLYKLDLR-a5-b4 | OKP1 | AME1 | 103 | 172 | 563,82 | 4 | 0,8 | 13 | 6 | 40,72 |
| 282 | FEVGKESTGK-LLYKLDLR-a5-b4 | OKP1 | AME1 | 103 | 172 | 563,819 | 4 | -0,5 | 12 | 6 | 40,02 |
| 283 | FEVGKESTGK-LLYKLDLR-a5-b4 | OKP1 | AME1 | 103 | 172 | 751,423 | 3 | -0,9 | 11 | 2 | 35,72 |
| 284 | ESTGKLQSHLSDGSATSGEGNVRPWEFR-LLYKLDLR-a5-b4 | OKP1 | AME1 | 108 | 172 | 841,436 | 5 | 0,8 | 10 | 1 | 22,2 |
| 285 | KVIQAEYR-LLYKLDLR-a1-b4 | OKP1 | AME1 | 132 | 172 | 545,073 | 4 | -0,1 | 12 | 3 | 36,74 |
| 286 | KVIQAEYR-LLYKLDLR-a1-b4 | OKP1 | AME1 | 132 | 172 | 545,073 | 4 | -0,3 | 11 | 1 | 33,45 |
| 287 | KVIQAEYR-LNDKVK-a1-b4 | OKP1 | AME1 | 132 | 249 | 620,692 | 3 | -0,1 | 6 | 1 | 26,61 |
| 288 | NYELKHWK-LNDKVK-a5-b4 | OKP1 | AME1 | 149 | 249 | 657,695 | 3 | -1 | 8 | 3 | 33,69 |
| 289 | NYELKHWK-KLLYK-a5-b1 | OKP1 | AME1 | 149 | 168 | 480,526 | 4 | -0,1 | 9 | 2 | 33,39 |
| 290 | NYELKHWK-DTKLAFR-a5-b3 | OKP1 | AME1 | 149 | 6 | 702,378 | 3 | -0,2 | 6 | 1 | 27,69 |
| 291 | LLETNTVSALDSVFEKYEK-LLYKLDLR-a16-b4 | OKP1 | AME1 | 180 | 172 | 839,961 | 4 | -1,5 | 9 | 21 | 33,7 |
| 292 | LLETNTVSALDSVFEKYEK-KLLYKLDLR-a16-b5 | OKP1 | AME1 | 180 | 172 | 697,789 | 5 | -1,8 | 5 | 12 | 32,94 |
| 293 | LLETNTVSALDSVFEKYEK-KLLYKLDLR-a16-b5 | OKP1 | AME1 | 180 | 172 | 697,791 | 5 | 0,7 | 5 | 4 | 32,44 |
| 294 | LLETNTVSALDSVFEKYEK-KLLYKLDLR-a16-b1 | OKP1 | AME1 | 180 | 168 | 697,79 | 5 | -0,2 | 6 | 2 | 31,47 |
| 295 | YEKEMNQMTHGDNNEVKR-LLYKLDLR-a3-b4 | OKP1 | AME1 | 183 | 172 | 566,455 | 6 | -1 | 12 | 4 | 37,31 |
| 296 | YEKEMNQMTHGDNNEVK-LLYKLDLR-a3-b4 | OKP1 | AME1 | 183 | 172 | 810,154 | 4 | 0,6 | 11 | 12 | 35,14 |
| 297 | YEKEMNQMTHGDNNEVKR-LLYKLDLR-a3-b4 | OKP1 | AME1 | 183 | 172 | 566,456 | 6 | 0,4 | 12 | 6 | 34,4 |
| 298 | YEKEMNQMTHGDNNEVK-LLYKLDLR-a3-b4 | OKP1 | AME1 | 183 | 172 | 648,326 | 5 | 1,8 | 12 | 11 | 32,75 |
| 299 | YEKEMNQMTHGDNNEVKR-LLYKLDLR-a3-b4 | OKP1 | AME1 | 183 | 172 | 566,455 | 6 | -0,2 | 11 | 14 | 31,47 |
| 300 | YEKEMNQMTHGDNNEVKR-KLLYKLDLR-a3-b5 | OKP1 | AME1 | 183 | 172 | 587,804 | 6 | -1,1 | 7 | 3 | 29,06 |
| 301 | EMNQMTHGDNNEVKR-LLYKLDLR-a14-b4 | OKP1 | AME1 | 197 | 172 | 744,13 | 4 | 0,7 | 9 | 1 | 28,08 |
| 302 | IYSKK-KLLYK-a4-b1 | OKP1 | AME1 | 202 | 168 | 360,727 | 4 | -1,2 | 5 | 2 | 33,53 |
| 303 | LCMNLKTNNK-NEIQELKAGK-a6-b7 | OKP1 | AME1 | 280 | 231 | 626,332 | 4 | 0,1 | 13 | 3 | 39,82 |
| 304 | LCMNLKTNNKK-LNDKVK-a6-b4 | OKP1 | AME1 | 280 | 249 | 444,249 | 5 | 1,3 | 9 | 5 | 33,31 |
| 305 | LCMNLKTNNK-NEIQELKAGK-a6-b7 | OKP1 | AME1 | 280 | 231 | 626,333 | 4 | 0,3 | 8 | 2 | 31,86 |
| 306 | LCMNLKTNNKK-NEIQELKAGK-a6-b7 | OKP1 | AME1 | 280 | 231 | 877,472 | 3 | -0,3 | 11 | 8 | 30,83 |
| 307 | LCMNLKTNNK-NEIQELKAGK-a6-b7 | OKP1 | AME1 | 280 | 231 | 626,331 | 4 | -1,4 | 7 | 2 | 26,24 |
| 308 | LTEKLIQK-LNDKVK-a4-b4 | OKP1 | AME1 | 290 | 249 | 457,281 | 4 | 0,2 | 9 | 6 | 40,04 |
| 309 | LTEKLIQK-LNDKVK-a4-b4 | OKP1 | AME1 | 290 | 249 | 457,28 | 4 | -1,2 | 9 | 6 | 39,08 |
| 310 | RLTEKLIQK-VKLNK-a5-b2 | OKP1 | AME1 | 290 | 251 | 467,549 | 4 | -0,9 | 9 | 16 | 38,77 |
| 311 | LTEKLIQK-VKLNK-a4-b2 | OKP1 | AME1 | 290 | 251 | 428,523 | 4 | -1,6 | 7 | 2 | 37,21 |
| 312 | LTEKLIQK-LNDKVK-a4-b4 | OKP1 | AME1 | 290 | 249 | 457,28 | 4 | -1 | 9 | 3 | 37,18 |
| 313 | RLTEKLIQK-VKLNKR-a5-b2 | OKP1 | AME1 | 290 | 251 | 506,574 | 4 | -1,2 | 9 | 3 | 35,9 |
| 314 | RLTEKLIQK-LNDKVK-a5-b4 | OKP1 | AME1 | 290 | 249 | 496,307 | 4 | 1,1 | 8 | 6 | 35 |
| 315 | RLTEKLIQK-LNDKVK-a5-b4 | OKP1 | AME1 | 290 | 249 | 661,404 | 3 | -2,4 | 8 | 13 | 34,24 |
| 316 | RLTEKLIQK-VKLNK-a5-b2 | OKP1 | AME1 | 290 | 251 | 467,549 | 4 | -1,8 | 8 | 2 | 33,82 |
| 317 | RLTEKLIQK-VKLNK-a5-b2 | OKP1 | AME1 | 290 | 251 | 467,55 | 4 | 0,8 | 9 | 2 | 33 |
| 318 | RLTEKLIQK-LNDKVK-a5-b4 | OKP1 | AME1 | 290 | 249 | 496,307 | 4 | 1 | 7 | 3 | 29,64 |
| 319 | LTEKLIQK-VKLNKR-a4-b2 | OKP1 | AME1 | 290 | 251 | 623,063 | 3 | -0,4 | 4 | 3 | 28,34 |
| 320 | LIQKDLHPVLNK-NEIQELKAGK-a4-b7 | OKP1 | AME1 | 294 | 231 | 537,713 | 5 | 0 | 14 | 8 | 39,82 |
| 321 | LIQKDLHPVLNK-LNDKVK-a4-b4 | OKP1 | AME1 | 294 | 249 | 455,075 | 5 | -1 | 11 | 5 | 39,74 |
| 322 | LIQKDLHPVLNK-NEIQELKAGK-a4-b7 | OKP1 | AME1 | 294 | 231 | 537,713 | 5 | -0,9 | 12 | 8 | 39,1 |
| 323 | LIQKDLHPVLNK-LNDKVK-a4-b4 | OKP1 | AME1 | 294 | 249 | 455,075 | 5 | 0,6 | 9 | 2 | 37,67 |
| 324 | LIQKDLHPVLNK-VKLNKR-a4-b5 | OKP1 | AME1 | 294 | 254 | 463,29 | 5 | -0,2 | 6 | 2 | 33,2 |
| 325 | NDSHELNLMLNDPIKSTADVR-NEIQELKAGK-a15-b7 | OKP1 | AME1 | 341 | 231 | 730,577 | 5 | 0 | 5 | 1 | 27,07 |
| 326 | LDKEEVLSLLPSLK-LLYKLDLR-a3-b4 | OKP1 | AME1 | 350 | 172 | 689,412 | 4 | -0,5 | 9 | 2 | 34,51 |
| 327 | LDKEEVLSLLPSLK-NEIQELKAGK-a3-b7 | OKP1 | AME1 | 350 | 231 | 713,408 | 4 | 0,7 | 9 | 2 | 34,41 |
| 328 | LDKEEVLSLLPSLK-NEIQELKAGK-a3-b7 | OKP1 | AME1 | 350 | 231 | 713,408 | 4 | 0,1 | 8 | 4 | 33,08 |
| 329 | ELKETMGQMISDSHEEEIK-NEIQELKAGK-a3-b7 | OKP1 | AME1 | 371 | 231 | 875,933 | 4 | -1,4 | 10 | 2 | 25,19 |
| 330 | EVFVPHHESHQDKTEEDIH-LLYKLDLR-a13-b4 | OKP1 | AME1 | 400 | 172 | 697,558 | 5 | 0,5 | 9 | 1 | 24,32 |
| 331 | KVIQAEYR-WLPSKALR-a1-b5 | OKP1 | CHL4 | 132 | 110 | 705,41 | 3 | 1,3 | 7 | 1 | 25,32 |
| 332 | KVIQAEYR-SPLHDYKK-a1-b7 | OKP1 | CHL4 | 132 | 287 | 711,057 | 3 | 0,6 | 6 | 2 | 24,47 |
| 333 | DLDIEYIYSKR-FQGKPLISR-a10-b4 | OKP1 | CHL4 | 240 | 192 | 866,47 | 3 | -1,1 | 10 | 6 | 36,14 |
| 334 | ISERDLDIEYIYSKR-FQGKPLISR-a14-b4 | OKP1 | CHL4 | 240 | 192 | 771,419 | 4 | -1,1 | 11 | 2 | 33,36 |
| 335 | DLDIEYIYSKR-FQGKPLISR-a10-b4 | OKP1 | CHL4 | 240 | 192 | 650,103 | 4 | -3,9 | 5 | 3 | 30,69 |
| 336 | ISERDLDIEYIYSKR-FQGKPLISR-a14-b4 | OKP1 | CHL4 | 240 | 192 | 617,338 | 5 | -0,4 | 5 | 1 | 26,62 |
| 337 | KPSKIMIGSILR-ATKNLFPR-a1-b3 | OKP1 | CSE4 | 153 | 49 | 607,364 | 4 | -0,4 | 9 | 1 | 30,99 |
| 338 | KPSKIMIGSILR-ATKNLFPR-a4-b3 | OKP1 | CSE4 | 156 | 49 | 486,092 | 5 | -1,1 | 10 | 4 | 38,23 |
| 339 | KPSKIMIGSILR-ATKNLFPR-a4-b3 | OKP1 | CSE4 | 156 | 49 | 486,092 | 5 | -1,8 | 9 | 4 | 36,18 |
| 340 | KPSKIMIGSILR-ATKNLFPR-a4-b3 | OKP1 | CSE4 | 156 | 49 | 486,093 | 5 | 0,1 | 9 | 5 | 36,14 |
| 341 | EMNQMTHGDNNEVKR-ATKNLFPR-a14-b3 | OKP1 | CSE4 | 197 | 49 | 722,354 | 4 | -2,3 | 8 | 11 | 25,05 |
| 342 | EMNQMTHGDNNEVKR-ATKNLFPR-a14-b3 | OKP1 | CSE4 | 197 | 49 | 722,355 | 4 | -1,2 | 8 | 7 | 22,61 |
| 343 | IYSKK-REKQR-a4-b3 | OKP1 | CSE4 | 202 | 119 | 497,96 | 3 | -0,3 | 5 | 2 | 30,93 |
| 344 | LLETNTVSALDSVFEKYEK-AKQLLATR-a16-b2 | OKP1 | CTF19 | 180 | 229 | 806,694 | 4 | 1,8 | 5 | 5 | 29,41 |
| 345 | RLTEKLIQK-KRVER-a5-b1 | OKP1 | CTF19 | 290 | 123 | 489,055 | 4 | 0,6 | 4 | 2 | 25,72 |
| 346 | EVFVPHHESHQDKTEEDIH-AKQLLATR-a13-b2 | OKP1 | CTF19 | 400 | 229 | 670,941 | 5 | -0,8 | 10 | 5 | 29,66 |
| 347 | EVFVPHHESHQDKTEEDIH-AKQLLATR-a13-b2 | OKP1 | CTF19 | 400 | 229 | 559,285 | 6 | -1 | 9 | 1 | 25,89 |
| 348 | TNKEEGQYHHK-GGKAGSAAK-a3-b3 | OKP1 | H2A1 | 84 | 8 | 564,286 | 4 | -0,3 | 8 | 2 | 28,31 |
| 349 | RIYSKK-GKGGK-a5-b2 | OKP1 | H4 | 202 | 6 | 345,211 | 4 | -1,5 | 8 | 3 | 34,86 |
| 350 | DFNKSDDDQFR-LLYKLDLR-a4-b4 | AME1 | AME1 | 160 | 172 | 640,079 | 4 | -0,8 | 11 | 2 | 39,31 |
| 351 | DFNKSDDDQFR-LLYKLDLR-a4-b4 | AME1 | AME1 | 160 | 172 | 640,078 | 4 | -1,6 | 11 | 3 | 39,17 |
| 352 | DFNKSDDDQFRK-LLYKLDLR-a4-b4 | AME1 | AME1 | 160 | 172 | 537,884 | 5 | -0,2 | 11 | 10 | 38,84 |
| 353 | DFNKSDDDQFR-NEIQELKAGK-a4-b7 | AME1 | AME1 | 160 | 231 | 885,097 | 3 | 0,5 | 14 | 3 | 37,51 |
| 354 | DFNKSDDDQFRK-LLYKLDLR-a4-b4 | AME1 | AME1 | 160 | 172 | 537,885 | 5 | 1,2 | 11 | 10 | 37,31 |
| 355 | DFNKSDDDQFRK-VKLNK-a4-b2 | AME1 | AME1 | 160 | 251 | 564,043 | 4 | -1,2 | 7 | 8 | 35,81 |
| 356 | DFNKSDDDQFR-VKLNK-a4-b2 | AME1 | AME1 | 160 | 251 | 709,023 | 3 | -1,3 | 6 | 2 | 35,55 |
| 357 | DFNKSDDDQFRK-LLYKLDLR-a4-b4 | AME1 | AME1 | 160 | 172 | 537,883 | 5 | -1,1 | 10 | 5 | 35,02 |
| 358 | DFNKSDDDQFRK-NEIQELKAGK-a4-b7 | AME1 | AME1 | 160 | 231 | 557,08 | 5 | 0,4 | 11 | 6 | 34,71 |
| 359 | DFNKSDDDQFRK-LNDKVK-a4-b4 | AME1 | AME1 | 160 | 249 | 592,801 | 4 | 0,8 | 7 | 4 | 33,86 |
| 360 | DFNKSDDDQFR-KLLYK-a4-b1 | AME1 | AME1 | 160 | 168 | 730,036 | 3 | -0,2 | 5 | 4 | 33,67 |
| 361 | DFNKSDDDQFRK-KLLYK-a4-b1 | AME1 | AME1 | 160 | 168 | 579,802 | 4 | -1,4 | 6 | 5 | 33,32 |
| 362 | DFNKSDDDQFR-KLLYK-a4-b1 | AME1 | AME1 | 160 | 168 | 730,036 | 3 | 0,1 | 5 | 6 | 32,13 |
| 363 | KLLYK-VKLNK-a1-b2 | AME1 | AME1 | 168 | 251 | 351,482 | 4 | -0,5 | 6 | 2 | 36,16 |
| 364 | LLYKLDLR-LNDKVK-a4-b4 | AME1 | AME1 | 172 | 249 | 472,539 | 4 | 0,7 | 9 | 6 | 42,49 |
| 365 | LLYKLDLR-DTKLAFR-a4-b3 | AME1 | AME1 | 172 | 6 | 506,051 | 4 | 0,1 | 10 | 4 | 41,14 |
| 366 | LLYKLDLR-KLLYK-a4-b1 | AME1 | AME1 | 172 | 168 | 459,541 | 4 | -0,6 | 4 | 3 | 38,84 |
| 367 | DLKDILDINVSNNELCYQLK-KEDLNQQIISVR-a3-b1 | AME1 | AME1 | 190 | 213 | 1329,697 | 3 | 0,3 | 11 | 4 | 31,96 |
| 368 | DILDINVSNNELCYQLKQVLAR-LNDLTSTLLGKYEGDRK-a17-b11 | AME1 | AME1 | 207 | 266 | 936,494 | 5 | -0,8 | 8 | 1 | 28,82 |
| 369 | KEDLNQQIISVR-NEIQELKAGK-a1-b7 | AME1 | AME1 | 213 | 231 | 678,125 | 4 | -1,4 | 11 | 5 | 37,54 |
| 370 | KEDLNQQIISVR-NEIQELKAGK-a1-b7 | AME1 | AME1 | 213 | 231 | 903,83 | 3 | -1,2 | 11 | 4 | 36,28 |
| 371 | KEDLNQQIISVR-VKLNK-a1-b2 | AME1 | AME1 | 213 | 251 | 727,759 | 3 | 0,8 | 6 | 4 | 33,57 |
| 372 | KEDLNQQIISVR-NEIQELKAGK-a1-b7 | AME1 | AME1 | 213 | 231 | 678,126 | 4 | 0,8 | 8 | 2 | 32,59 |
| 373 | KEDLNQQIISVR-LLYKLDLR-a1-b4 | AME1 | AME1 | 213 | 172 | 871,838 | 3 | -0,4 | 8 | 2 | 31,9 |
| 374 | KEDLNQQIISVR-LLYKLDLR-a1-b4 | AME1 | AME1 | 213 | 172 | 871,837 | 3 | -1,2 | 9 | 2 | 30 |
| 375 | NEIQELKAGK-VKLNK-a7-b2 | AME1 | AME1 | 231 | 251 | 467,778 | 4 | 1,4 | 7 | 6 | 40,17 |
| 376 | NEIQELKAGK-LLYKLDLR-a7-b4 | AME1 | AME1 | 231 | 172 | 575,838 | 4 | 2 | 12 | 4 | 39,94 |
| 377 | NEIQELKAGK-VKLNK-a7-b2 | AME1 | AME1 | 231 | 251 | 467,777 | 4 | -1,1 | 7 | 5 | 38,36 |
| 378 | NEIQELKAGK-LLYKLDLR-a7-b4 | AME1 | AME1 | 231 | 172 | 767,445 | 3 | -1 | 10 | 4 | 38,1 |
| 379 | NEIQELKAGK-LNDKVK-a7-b4 | AME1 | AME1 | 231 | 249 | 496,534 | 4 | 0,4 | 7 | 5 | 37,83 |
| 380 | NEIQELKAGK-LLYKLDLR-a7-b4 | AME1 | AME1 | 231 | 172 | 767,444 | 3 | -3,1 | 9 | 1 | 35,61 |
| 381 | NEIQELKAGK-KLLYK-a7-b1 | AME1 | AME1 | 231 | 168 | 483,536 | 4 | -0,4 | 6 | 7 | 35,56 |
| 382 | NEIQELKAGK-DTKLAFR-a7-b3 | AME1 | AME1 | 231 | 6 | 530,045 | 4 | -1,2 | 10 | 2 | 34,66 |
| 383 | NEIQELKAGK-VKLNKR-a7-b2 | AME1 | AME1 | 231 | 251 | 506,803 | 4 | 0,6 | 4 | 2 | 22,99 |
| 384 | AGKDWHDLQNEQAK-VKLNK-a3-b2 | AME1 | AME1 | 234 | 251 | 595,318 | 4 | 0,2 | 7 | 5 | 39,43 |
| 385 | AGKDWHDLQNEQAK-LNDKVK-a3-b4 | AME1 | AME1 | 234 | 249 | 499,461 | 5 | -0,6 | 8 | 3 | 38,14 |
| 386 | AGKDWHDLQNEQAK-VKLNK-a3-b2 | AME1 | AME1 | 234 | 251 | 595,318 | 4 | -0,1 | 8 | 4 | 38,13 |
| 387 | AGKDWHDLQNEQAK-LLYKLDLR-a3-b4 | AME1 | AME1 | 234 | 172 | 703,377 | 4 | 0 | 13 | 4 | 36,04 |
| 388 | AGKDWHDLQNEQAK-LNDKVK-a3-b4 | AME1 | AME1 | 234 | 249 | 624,075 | 4 | 1,5 | 7 | 3 | 32,29 |
| 389 | AGKDWHDLQNEQAK-DFNKSDDDQFRK-a3-b4 | AME1 | AME1 | 234 | 160 | 659,111 | 5 | -1,8 | 12 | 1 | 31,24 |
| 390 | AGKDWHDLQNEQAK-KLLYK-a3-b1 | AME1 | AME1 | 234 | 168 | 611,076 | 4 | -0,5 | 5 | 6 | 30,49 |
| 391 | AGKDWHDLQNEQAK-NEIQELKAGK-a3-b7 | AME1 | AME1 | 234 | 231 | 969,495 | 3 | 1,1 | 9 | 4 | 30,03 |
| 392 | AGKDWHDLQNEQAK-KEDLNQQIISVR-a3-b1 | AME1 | AME1 | 234 | 213 | 805,666 | 4 | 0,4 | 8 | 2 | 27,36 |
| 393 | AGKDWHDLQNEQAK-KEDLNQQIISVR-a3-b1 | AME1 | AME1 | 234 | 213 | 805,668 | 4 | 3,1 | 6 | 3 | 26,02 |
| 394 | DWHDLQNEQAKLNDK-VKLNK-a11-b2 | AME1 | AME1 | 245 | 251 | 864,784 | 3 | -2 | 7 | 9 | 38,29 |
| 395 | DWHDLQNEQAKLNDK-VKLNK-a11-b2 | AME1 | AME1 | 245 | 251 | 864,786 | 3 | -0,5 | 7 | 15 | 37,23 |
| 396 | DWHDLQNEQAKLNDK-NEIQELKAGK-a11-b7 | AME1 | AME1 | 245 | 231 | 624,918 | 5 | -0,4 | 10 | 6 | 36,58 |
| 397 | DWHDLQNEQAKLNDK-NEIQELKAGK-a11-b7 | AME1 | AME1 | 245 | 231 | 780,896 | 4 | -0,1 | 15 | 5 | 32,64 |
| 398 | DWHDLQNEQAKLNDK-KEDLNQQIISVR-a11-b1 | AME1 | AME1 | 245 | 213 | 859,192 | 4 | 2,9 | 9 | 1 | 28,97 |
| 399 | DWHDLQNEQAKLNDK-VKLNKR-a11-b2 | AME1 | AME1 | 245 | 251 | 687,866 | 4 | -0,3 | 5 | 2 | 27,89 |
| 400 | DWHDLQNEQAKLNDK-LNDKVK-a11-b4 | AME1 | AME1 | 245 | 249 | 677,598 | 4 | 0 | 6 | 1 | 26,39 |
| 401 | DWHDLQNEQAKLNDK-NEIQELKAGK-a11-b7 | AME1 | AME1 | 245 | 231 | 780,896 | 4 | -0,4 | 8 | 2 | 24,89 |
| 402 | LNDKVK-KLLYK-a4-b1 | AME1 | AME1 | 249 | 168 | 380,238 | 4 | -0,9 | 9 | 2 | 36,53 |
| 403 | VKLNKR-KLLYK-a2-b1 | AME1 | AME1 | 251 | 168 | 390,507 | 4 | -0,6 | 7 | 1 | 32,9 |
| 404 | LNDLTSTLLGKYEGDRK-LNDKVK-a11-b4 | AME1 | AME1 | 266 | 249 | 556,109 | 5 | 2,1 | 10 | 2 | 38,87 |
| 405 | LNDLTSTLLGKYEGDRK-LLYKLDLR-a11-b4 | AME1 | AME1 | 266 | 172 | 619,55 | 5 | 0,4 | 11 | 9 | 37,36 |
| 406 | LNDLTSTLLGKYEGDR-LNDKVK-a11-b4 | AME1 | AME1 | 266 | 249 | 662,858 | 4 | -1,6 | 8 | 1 | 37,21 |
| 407 | LNDLTSTLLGKYEGDR-NEIQELKAGK-a11-b7 | AME1 | AME1 | 266 | 231 | 766,156 | 4 | -2,2 | 10 | 3 | 34,24 |
| 408 | LNDLTSTLLGKYEGDRK-KEDLNQQIISVR-a11-b1 | AME1 | AME1 | 266 | 213 | 701,382 | 5 | 0,9 | 12 | 6 | 33,42 |
| 409 | LNDLTSTLLGKYEGDRK-LLYKLDLR-a11-b4 | AME1 | AME1 | 266 | 172 | 774,184 | 4 | -2,5 | 11 | 3 | 33,25 |
| 410 | LNDLTSTLLGKYEGDR-KLLYKLDLR-a11-b5 | AME1 | AME1 | 266 | 172 | 619,551 | 5 | 1 | 9 | 1 | 31,62 |
| 411 | LNDLTSTLLGKYEGDR-NEIQELKAGK-a11-b7 | AME1 | AME1 | 266 | 231 | 766,156 | 4 | -1,5 | 10 | 3 | 31,49 |
| 412 | LNDLTSTLLGKYEGDR-KEDLNQQIISVR-a11-b1 | AME1 | AME1 | 266 | 213 | 844,448 | 4 | -3,7 | 10 | 2 | 27,42 |
| 413 | LNDLTSTLLGKYEGDRK-KEDLNQQIISVR-a11-b1 | AME1 | AME1 | 266 | 213 | 701,382 | 5 | 1 | 8 | 2 | 27,22 |
| 414 | GDGKPYVVK-WLPSKALR-a4-b5 | CHL4 | CHL4 | 117 | 110 | 518,3 | 4 | 0,2 | 9 | 4 | 39,66 |
| 415 | GDGKPYVVK-KHQGLTGK-a4-b1 | CHL4 | CHL4 | 117 | 288 | 656,703 | 3 | 0,4 | 9 | 3 | 30,03 |
| 416 | GDGKPYVVKLQPAK-KVMVR-a9-b1 | CHL4 | CHL4 | 122 | 296 | 568,083 | 4 | -1,2 | 5 | 4 | 32,95 |
| 417 | GDGKPYVVKLQPAK-KNEDSGEPVYISR-a9-b1 | CHL4 | CHL4 | 122 | 363 | 783,417 | 4 | 0,7 | 8 | 1 | 29,63 |
| 418 | GDGKPYVVKLQPAK-SPLHDYKK-a9-b7 | CHL4 | CHL4 | 122 | 287 | 875,486 | 3 | -0,7 | 7 | 4 | 29,09 |
| 419 | GDGKPYVVKLQPAK-WLPSKALR-a9-b5 | CHL4 | CHL4 | 122 | 110 | 522,306 | 5 | -0,9 | 5 | 1 | 28,55 |
| 420 | IYHCHVYMFKHPSLPVLITR-GDGKPYVVKLQPAK-a10-b9 | CHL4 | CHL4 | 148 | 122 | 692,211 | 6 | -1,1 | 9 | 8 | 32,48 |
| 421 | IYHCHVYMFKHPSLPVLITR-WLPSKALR-a10-b5 | CHL4 | CHL4 | 148 | 110 | 603,999 | 6 | -1,3 | 7 | 3 | 31,99 |
| 422 | IYHCHVYMFKHPSLPVLITR-KVMVR-a10-b1 | CHL4 | CHL4 | 148 | 296 | 547,635 | 6 | 0,1 | 5 | 3 | 31,93 |
| 423 | IYHCHVYMFKHPSLPVLITR-KHQGLTGK-a10-b1 | CHL4 | CHL4 | 148 | 288 | 586,986 | 6 | -0,7 | 5 | 5 | 31,35 |
| 424 | IYHCHVYMFKHPSLPVLITR-SPLHDYKK-a10-b7 | CHL4 | CHL4 | 148 | 287 | 606,823 | 6 | -0,6 | 6 | 2 | 28,86 |
| 425 | IYHCHVYMFKHPSLPVLITR-GDGKPYVVKLQPAK-a10-b9 | CHL4 | CHL4 | 148 | 122 | 692,211 | 6 | -1,1 | 6 | 3 | 27,01 |
| 426 | ESLYNKLDK-FQGKPLISR-a6-b4 | CHL4 | CHL4 | 185 | 192 | 764,758 | 3 | -0,5 | 11 | 9 | 33,38 |
| 427 | ESLYNKLDK-FQGKPLISR-a6-b4 | CHL4 | CHL4 | 185 | 192 | 764,758 | 3 | -0,6 | 11 | 6 | 29,36 |
| 428 | FQGKPLISR-WLPSKALR-a4-b5 | CHL4 | CHL4 | 192 | 110 | 539,071 | 4 | -0,2 | 6 | 1 | 28,59 |
| 429 | RPYYVAFPLNSPIIFHSVDKDIYAR-ETIIFKPVQKIPVK-a20-b10 | CHL4 | CHL4 | 217 | 245 | 952,535 | 5 | 0,5 | 9 | 2 | 27,27 |
| 430 | RPYYVAFPLNSPIIFHSVDKDIYAR-ETIIFKPVQKIPVK-a20-b6 | CHL4 | CHL4 | 217 | 241 | 952,535 | 5 | 0,1 | 7 | 2 | 24,88 |
| 431 | RPYYVAFPLNSPIIFHSVDKDIYAR-ETIIFKPVQK-a20-b6 | CHL4 | CHL4 | 217 | 241 | 1081,094 | 4 | 2,2 | 5 | 3 | 23,44 |
| 432 | ETIIFKPVQK-WLPSKALR-a6-b5 | CHL4 | CHL4 | 241 | 110 | 578,345 | 4 | -1,1 | 9 | 14 | 36,03 |
| 433 | ETIIFKPVQK-GDGKPYVVK-a6-b4 | CHL4 | CHL4 | 241 | 117 | 576,333 | 4 | 0,2 | 10 | 8 | 35,89 |
| 434 | TISERETIIFKPVQK-WLPSKALR-a11-b5 | CHL4 | CHL4 | 241 | 110 | 580,138 | 5 | -2,2 | 5 | 4 | 33,97 |
| 435 | ETIIFKPVQK-WLPSKALR-a6-b5 | CHL4 | CHL4 | 241 | 110 | 578,345 | 4 | -1,5 | 6 | 14 | 33,53 |
| 436 | TISERETIIFKPVQK-WLPSKALR-a11-b5 | CHL4 | CHL4 | 241 | 110 | 580,14 | 5 | 0,1 | 6 | 4 | 30,52 |
| 437 | ETIIFKPVQK-KHQGLTGK-a6-b1 | CHL4 | CHL4 | 241 | 288 | 552,823 | 4 | -2,6 | 6 | 6 | 30,48 |
| 438 | ETIIFKPVQK-GDGKPYVVK-a6-b4 | CHL4 | CHL4 | 241 | 117 | 576,332 | 4 | -1,2 | 9 | 4 | 29,36 |
| 439 | ETIIFKPVQKIPVK-WLPSKALR-a6-b5 | CHL4 | CHL4 | 241 | 110 | 550,338 | 5 | -0,6 | 6 | 1 | 24,67 |
| 440 | ETIIFKPVQKIPVK-WLPSKALR-a10-b5 | CHL4 | CHL4 | 245 | 110 | 687,671 | 4 | -0,1 | 8 | 7 | 29,76 |
| 441 | IPVKSIHNIMTLLGPSR-FKWLPSK-a4-b2 | CHL4 | CHL4 | 249 | 105 | 730,423 | 4 | 0,1 | 4 | 2 | 26,23 |
| 442 | SPLHDYKK-KVMVR-a7-b1 | CHL4 | CHL4 | 287 | 296 | 440 | 4 | -0,7 | 8 | 5 | 33,47 |
| 443 | SPLHDYKK-WLPSKALR-a7-b5 | CHL4 | CHL4 | 287 | 110 | 524,548 | 4 | -1,1 | 5 | 2 | 27,77 |
| 444 | KHQGLTGKK-KVMVR-a1-b1 | CHL4 | CHL4 | 288 | 296 | 354,015 | 5 | -1,3 | 9 | 4 | 36,66 |
| 445 | KHQGLTGK-WLPSKALR-a1-b5 | CHL4 | CHL4 | 288 | 110 | 396,035 | 5 | -0,7 | 7 | 5 | 36,51 |
| 446 | KHQGLTGK-WLPSKALR-a1-b5 | CHL4 | CHL4 | 288 | 110 | 396,035 | 5 | -0,8 | 7 | 5 | 36,06 |
| 447 | KHQGLTGK-SPLHDYKK-a1-b7 | CHL4 | CHL4 | 288 | 287 | 399,423 | 5 | -1,1 | 10 | 4 | 33,25 |
| 448 | KHQGLTGK-KVMVR-a1-b1 | CHL4 | CHL4 | 288 | 296 | 328,396 | 5 | -1,2 | 9 | 16 | 32,53 |
| 449 | KHQGLTGK-KVMVR-a1-b1 | CHL4 | CHL4 | 288 | 296 | 410,244 | 4 | -1,1 | 6 | 6 | 29,71 |
| 450 | KHQGLTGKK-HQGLTGKK-a1-b7 | CHL4 | CHL4 | 288 | 295 | 401,237 | 5 | -0,6 | 7 | 1 | 29,08 |
| 451 | KHQGLTGK-SPLHDYKK-a1-b7 | CHL4 | CHL4 | 288 | 287 | 399,423 | 5 | -2,3 | 8 | 1 | 27 |
| 452 | HQGLTGKK-SPLHDYKK-a7-b7 | CHL4 | CHL4 | 295 | 287 | 499,028 | 4 | 0 | 7 | 14 | 33,59 |
| 453 | HQGLTGKK-SPLHDYKK-a7-b7 | CHL4 | CHL4 | 295 | 287 | 499,027 | 4 | -1,3 | 8 | 5 | 27,79 |
| 454 | EFDDSFLNDDENFYGKEEPEIR-WLPSKALR-a16-b5 | CHL4 | CHL4 | 316 | 110 | 1272,606 | 3 | -1,7 | 5 | 1 | 24,53 |
| 455 | EFDDSFLNDDENFYGKEEPEIR-GDGKPYVVKLQPAK-a16-b9 | CHL4 | CHL4 | 316 | 122 | 869,824 | 5 | 1,9 | 5 | 1 | 24,5 |
| 456 | GSANGVMDQKYNDLK-KNEDSGEPVYISR-a10-b1 | CHL4 | CHL4 | 344 | 363 | 818,395 | 4 | -0,7 | 15 | 4 | 35,32 |
| 457 | YNDLKEFNEHVHNIR-KVMVR-a5-b1 | CHL4 | CHL4 | 349 | 296 | 450,405 | 6 | -1 | 10 | 7 | 38,87 |
| 458 | YNDLKEFNEHVHNIR-KVMVR-a5-b1 | CHL4 | CHL4 | 349 | 296 | 450,405 | 6 | -1,4 | 6 | 6 | 35,11 |
| 459 | YNDLKEFNEHVHNIR-KHQGLTGK-a5-b1 | CHL4 | CHL4 | 349 | 288 | 489,756 | 6 | -1,9 | 7 | 13 | 33,31 |
| 460 | YNDLKEFNEHVHNIR-KNEDSGEPVYISR-a5-b1 | CHL4 | CHL4 | 349 | 363 | 712,549 | 5 | -2,8 | 11 | 6 | 32,68 |
| 461 | YNDLKEFNEHVHNIR-KNEDSGEPVYISR-a5-b1 | CHL4 | CHL4 | 349 | 363 | 712,551 | 5 | -0,1 | 9 | 9 | 31,38 |
| 462 | YNDLKEFNEHVHNIR-GDGKPYVVKLQPAK-a5-b4 | CHL4 | CHL4 | 349 | 117 | 713,778 | 5 | 0 | 9 | 1 | 28,59 |
| 463 | YNDLKEFNEHVHNIR-HQGLTGKK-a5-b7 | CHL4 | CHL4 | 349 | 295 | 734,13 | 4 | -2,2 | 5 | 3 | 27,99 |
| 464 | YNDLKEFNEHVHNIR-GDGKPYVVK-a5-b4 | CHL4 | CHL4 | 349 | 117 | 606,314 | 5 | 2,3 | 9 | 1 | 24,54 |
| 465 | KNEDSGEPVYISR-SPLHDYKK-a1-b7 | CHL4 | CHL4 | 363 | 287 | 873,442 | 3 | -0,5 | 11 | 2 | 33,58 |
| 466 | KNEDSGEPVYISR-KHQGLTGK-a1-b1 | CHL4 | CHL4 | 363 | 288 | 833,766 | 3 | -0,3 | 10 | 3 | 33,56 |
| 467 | KNEDSGEPVYISR-KHQGLTGK-a1-b1 | CHL4 | CHL4 | 363 | 288 | 833,765 | 3 | -2,6 | 8 | 1 | 33,37 |
| 468 | KNEDSGEPVYISR-SPLHDYKK-a1-b7 | CHL4 | CHL4 | 363 | 287 | 873,442 | 3 | -0,1 | 7 | 2 | 28,68 |
| 469 | YSSLVPIEKVGFTLK-IITIKLK-a9-b5 | CHL4 | CHL4 | 384 | 401 | 662,408 | 4 | -0,9 | 6 | 2 | 32,84 |
| 470 | YSSLVPIEKVGFTLK-EQVAKGGLL-a9-b5 | CHL4 | CHL4 | 384 | 454 | 911,521 | 3 | -0,2 | 7 | 2 | 29,56 |
| 471 | YSSLVPIEKVGFTLK-EQVAKGGLL-a9-b5 | CHL4 | CHL4 | 384 | 454 | 911,523 | 3 | 1,9 | 8 | 2 | 28,07 |
| 472 | YSSLVPIEKVGFTLK-ETIIFKPVQKIPVK-a9-b6 | CHL4 | CHL4 | 384 | 241 | 692,413 | 5 | 0,2 | 9 | 3 | 27,58 |
| 473 | VGFTLKNEINSR-IITIKLK-a6-b5 | CHL4 | CHL4 | 390 | 401 | 586,606 | 4 | -0,3 | 10 | 1290 | 42,33 |
| 474 | VGFTLKNEINSR-IITIKLK-a6-b5 | CHL4 | CHL4 | 390 | 401 | 586,605 | 4 | -2,2 | 7 | 117 | 35,31 |
| 475 | LKFNGNDIFGGLHELCDK-YSSLVPIEKVGFTLK-a2-b9 | CHL4 | CHL4 | 403 | 384 | 974,515 | 4 | 0,1 | 10 | 4 | 32 |
| 476 | LKFNGNDIFGGLHELCDK-EQVAKGGLL-a2-b5 | CHL4 | CHL4 | 403 | 454 | 782,907 | 4 | -1,7 | 10 | 4 | 30,78 |
| 477 | EQVAKGGLL-IITIKLK-a5-b5 | CHL4 | CHL4 | 454 | 401 | 627,399 | 3 | -0,4 | 10 | 4 | 36,21 |
| 478 | EQVAKGGLL-IITIKLK-a5-b5 | CHL4 | CHL4 | 454 | 401 | 627,399 | 3 | -1,8 | 9 | 4 | 31,74 |
| 479 | EQVAKGGLL-WLPSKALR-a5-b5 | CHL4 | CHL4 | 454 | 110 | 674,73 | 3 | -0,2 | 5 | 2 | 29,03 |
| 480 | EQVAKGGLL-WLPSKALR-a5-b5 | CHL4 | CHL4 | 454 | 110 | 674,729 | 3 | -1,8 | 7 | 2 | 26,91 |
| 481 | KQSLK-RVEKK-a1-b4 | CSE4 | CSE4 | 122 | 130 | 350,722 | 4 | -0,2 | 6 | 4 | 30,9 |
| 482 | STDLLISKIPFAR-LVKEVTDEFTTK-a8-b3 | CSE4 | CSE4 | 155 | 163 | 1003,226 | 3 | 0,4 | 11 | 8 | 34,33 |
| 483 | STDLLISKIPFAR-LVKEVTDEFTTK-a8-b3 | CSE4 | CSE4 | 155 | 163 | 1003,224 | 3 | -1,5 | 10 | 9 | 31,27 |
| 484 | STDLLISKIPFAR-LVKEVTDEFTTK-a8-b3 | CSE4 | CSE4 | 155 | 163 | 752,671 | 4 | -0,5 | 6 | 3 | 26,52 |
| 485 | LVKEVTDEFTTKDQDLR-STDLLISKIPFAR-a3-b8 | CSE4 | CSE4 | 163 | 155 | 909,494 | 4 | -1,7 | 6 | 1 | 22,2 |
| 486 | RITIMKK-KQSLK-a6-b1 | CSE4 | CSE4 | 215 | 122 | 408,258 | 4 | -0,5 | 8 | 4 | 32,59 |
| 487 | ALKLQEEK-KRVER-a3-b1 | CTF19 | CTF19 | 56 | 123 | 595,02 | 3 | -0,3 | 5 | 3 | 24,98 |
| 488 | ALKLQEEK-ENNSKTK-a3-b5 | CTF19 | CTF19 | 56 | 89 | 639,35 | 3 | 0,9 | 7 | 1 | 23,26 |
| 489 | LQEEKDALLTR-ALKLQEEK-a5-b3 | CTF19 | CTF19 | 61 | 56 | 804,452 | 3 | 0,5 | 8 | 3 | 33,64 |
| 490 | FEDSTLLKWEILR-AKQLLATR-a8-b2 | CTF19 | CTF19 | 214 | 229 | 672,633 | 4 | -1,2 | 15 | 2 | 39,92 |
| 491 | FEDSTLLKWEILR-AKQLLATR-a8-b2 | CTF19 | CTF19 | 214 | 229 | 672,634 | 4 | -0,4 | 13 | 3 | 39,59 |
| 492 | FEDSTLLKWEILR-AKQLLATR-a8-b2 | CTF19 | CTF19 | 214 | 229 | 672,634 | 4 | -0,7 | 11 | 5 | 38,17 |
| 493 | NFQKCLLSLYEFDK-AKQLLATR-a4-b2 | CTF19 | CTF19 | 239 | 229 | 711,385 | 4 | -0,2 | 9 | 1 | 34,04 |
| 494 | TGIFQNLINLLKR-AKQLLATR-a12-b2 | CTF19 | CTF19 | 266 | 229 | 642,64 | 4 | -1,6 | 13 | 2 | 40,96 |
| 495 | TGIFQNLINLLKR-EYGVKTGLK-a12-b5 | CTF19 | CTF19 | 266 | 351 | 666,139 | 4 | -0,9 | 7 | 1 | 29,27 |
| 496 | NCFLPMSKISIALWK-AKQLLATR-a8-b2 | CTF19 | CTF19 | 320 | 229 | 712,151 | 4 | -1 | 8 | 1 | 26,64 |
| 497 | ISIALWKGGER-AKQLLATR-a7-b2 | CTF19 | CTF19 | 327 | 229 | 567,587 | 4 | 0,4 | 15 | 1 | 39,85 |
| 498 | ISIALWKGGER-EYGVKTGLK-a7-b5 | CTF19 | CTF19 | 327 | 351 | 591,086 | 4 | 0,3 | 7 | 3 | 31,9 |
| 499 | EYGVKTGLK-AKQLLATR-a5-b2 | CTF19 | CTF19 | 351 | 229 | 508,802 | 4 | 1,4 | 6 | 2 | 28,33 |
| 500 | LDNLQDIKK-THKQVYPLR-a8-b3 | DSN1 | DSN1 | 99 | 22 | 473,87 | 5 | -1,7 | 10 | 5 | 34,28 |
| 501 | AKNQEEEGELEHLTK-SLHKLELK-a2-b4 | DSN1 | DSN1 | 309 | 303 | 572,707 | 5 | -1,6 | 8 | 7 | 28,82 |
| 502 | NQEEEGELEHLTKKSK-LESTKAETDYVDPK-a13-b5 | DSN1 | DSN1 | 322 | 330 | 908,702 | 4 | -1 | 12 | 4 | 30,25 |
| 503 | AKNQEEEGELEHLTKK-SKLESTK-a15-b2 | DSN1 | DSN1 | 322 | 325 | 563,297 | 5 | -2,1 | 6 | 4 | 29,05 |
| 504 | NQEEEGELEHLTKK-NLSILTSKVNAIK-a13-b8 | DSN1 | DSN1 | 322 | 450 | 1074,58 | 3 | -0,6 | 11 | 3 | 26,45 |
| 505 | NQEEEGELEHLTKK-SKLESTK-a13-b2 | DSN1 | DSN1 | 322 | 325 | 654,087 | 4 | -0,4 | 5 | 5 | 25,51 |
| 506 | AETDYVDPKR-LELKAK-a9-b4 | DSN1 | DSN1 | 339 | 307 | 508,78 | 4 | -0,3 | 5 | 3 | 30,23 |
| 507 | KALPK-KFSER-a1-b1 | DSN1 | DSN1 | 422 | 416 | 340,705 | 4 | -2 | 5 | 3 | 31,85 |
| 508 | RKALPK-KFSER-a2-b1 | DSN1 | DSN1 | 422 | 416 | 379,73 | 4 | -1,9 | 6 | 7 | 31,73 |
| 509 | KLLPNSK-KALPK-a1-b1 | DSN1 | DSN1 | 430 | 422 | 373,991 | 4 | -2,8 | 6 | 5 | 32,19 |
| 510 | SAKAGLTFPVGR-AGSAAKASQSR-a3-b6 | H2A1 | H2A1 | 22 | 14 | 594,327 | 4 | -0,4 | 12 | 3 | 38,03 |
| 511 | SAKAGLTFPVGR-AGSAAKASQSR-a3-b6 | H2A1 | H2A1 | 22 | 14 | 792,1 | 3 | 0,2 | 13 | 7 | 37,41 |
| 512 | SAKAGLTFPVGR-AGSAAKASQSR-a3-b6 | H2A1 | H2A1 | 22 | 14 | 792,099 | 3 | -0,8 | 11 | 4 | 34,02 |
| 513 | SAKAGLTFPVGR-GGKAGSAAK-a3-b3 | H2A1 | H2A1 | 22 | 8 | 696,393 | 3 | 1,1 | 6 | 4 | 31,79 |
| 514 | LLGNVTIAQGGVLPNIHQNLLPKK-ATKASQEL-a23-b3 | H2A1 | H2A1 | 120 | 127 | 881,256 | 4 | -1,7 | 8 | 1 | 28,58 |
| 515 | LLGNVTIAQGGVLPNIHQNLLPKK-ATKASQEL-a23-b3 | H2A1 | H2A1 | 120 | 127 | 881,258 | 4 | 0,8 | 6 | 2 | 28,29 |
| 516 | LLGNVTIAQGGVLPNIHQNLLPKK-KSAKATK-a23-b1 | H2A1 | H2A1 | 120 | 121 | 682,41 | 5 | 2,2 | 4 | 1 | 26,91 |
| 517 | KPASKAPAEK-KPAAK-a5-b1 | H2B1 | H2B1 | 12 | 18 | 560,002 | 3 | -0,2 | 6 | 5 | 37,48 |
| 518 | KPASKAPAEK-KPASKAPAEK-a5-b5 | H2B1 | H2B1 | 33 | 33 | 548,318 | 4 | 0,3 | 13 | 4 | 37,98 |
| 519 | APAEKKPAAK-KPASK-a5-b1 | H2B1 | H2B1 | 38 | 29 | 560,002 | 3 | 0,8 | 10 | 25 | 40,95 |
| 520 | KETYSSYIYK-LAAYNKK-a1-b6 | H2B1 | H2B1 | 38 | 89 | 742,73 | 3 | 1,7 | 12 | 8 | 32,66 |
| 521 | APAEKKPAAK-KPASK-a6-b1 | H2B1 | H2B1 | 39 | 29 | 560,002 | 3 | 0,6 | 7 | 3 | 32,8 |
| 522 | KPAAK-KPASK-a1-b1 | H2B1 | H2B1 | 39 | 29 | 394,581 | 3 | 0,1 | 6 | 2 | 32,03 |
| 523 | VLKQTHPDTGISQK-KPASKAPAEK-a3-b5 | H2B1 | H2B1 | 50 | 12 | 679,633 | 4 | 1,9 | 15 | 4 | 38,79 |
| 524 | VLKQTHPDTGISQK-KETYSSYIYK-a3-b1 | H2B1 | H2B1 | 50 | 38 | 990,852 | 3 | -1,8 | 16 | 4 | 37,46 |
| 525 | VLKQTHPDTGISQK-APAEKKPAAK-a3-b5 | H2B1 | H2B1 | 50 | 17 | 675,634 | 4 | 0,6 | 11 | 2 | 35,24 |
| 526 | VLKQTHPDTGISQK-APAEKKPAAK-a3-b6 | H2B1 | H2B1 | 50 | 18 | 540,708 | 5 | -0,2 | 7 | 2 | 31,53 |
| 527 | VLKQTHPDTGISQK-KPAAK-a3-b1 | H2B1 | H2B1 | 50 | 18 | 735,088 | 3 | 1 | 7 | 3 | 30,4 |
| 528 | KETYSSYIYK-LAAYNKK-a1-b6 | H2B1 | H2B1 | 59 | 110 | 742,728 | 3 | 0 | 11 | 7 | 33,5 |
| 529 | QTHPDTGISQKSMSILNSFVNDIFER-KETYSSYIYK-a11-b1 | H2B1 | H2B1 | 82 | 59 | 1096,543 | 4 | 0 | 8 | 4 | 27,64 |
| 530 | GLGKGGAK-GKGGK-a4-b2 | H4 | H4 | 13 | 6 | 318,443 | 4 | -0,2 | 5 | 5 | 39,81 |
| 531 | GLGKGGAK-GKGGK-a4-b2 | H4 | H4 | 13 | 6 | 318,443 | 4 | -1,1 | 6 | 5 | 39,1 |
| 532 | GLGKGGAK-GKGGK-a4-b2 | H4 | H4 | 13 | 6 | 318,442 | 4 | -1,8 | 7 | 4 | 39,05 |
| 533 | AVLKSFLESVIR-GGKGLGK-a4-b3 | H4 | H4 | 60 | 9 | 705,757 | 3 | 0,9 | 5 | 1 | 33,32 |
| 534 | DSVTYTEHAKR-GKGGK-a10-b2 | H4 | H4 | 78 | 6 | 473,249 | 4 | -0,2 | 6 | 2 | 30,06 |
| 535 | KTVTSLDVVYALK-DSVTYTEHAKR-a1-b10 | H4 | H4 | 80 | 78 | 960,85 | 3 | -0,1 | 12 | 3 | 33,01 |
| 536 | KTVTSLDVVYALKR-DSVTYTEHAKR-a1-b10 | H4 | H4 | 80 | 78 | 608,135 | 5 | 2,7 | 10 | 2 | 26,47 |
| 537 | KTVTSLDVVYALKR-DSVTYTEHAKR-a1-b10 | H4 | H4 | 80 | 78 | 608,133 | 5 | -1,7 | 9 | 3 | 24,05 |
| 538 | ENIEQITWFSSKLYFEPETQDK-LPLTSVILAGHTKIMR-a12-b13 | IML3 | IML3 | 146 | 215 | 1155,608 | 4 | -0,7 | 6 | 316 | 24,87 |
| 539 | AKLENEEILPEQEWVLK-IKSNSWFLFK-a2-b2 | MCM21 | MCM21 | 99 | 229 | 869,472 | 4 | 2,5 | 8 | 1 | 23,04 |
| 540 | IKDASGEIFVDR-IKSNSWFLFK-a2-b2 | MCM21 | MCM21 | 189 | 229 | 689,872 | 4 | 0,1 | 12 | 4 | 36,72 |
| 541 | IKDASGEIFVDR-IKSNSWFLFK-a2-b2 | MCM21 | MCM21 | 189 | 229 | 689,873 | 4 | 1 | 11 | 3 | 35,7 |
| 542 | IKDASGEIFVDR-IKSNSWFLFK-a2-b2 | MCM21 | MCM21 | 189 | 229 | 689,87 | 4 | -3 | 10 | 4 | 34,98 |
| 543 | IKDASGEIFVDR-RIKSNSWFLFK-a2-b3 | MCM21 | MCM21 | 189 | 229 | 728,899 | 4 | 1,5 | 7 | 1 | 29,81 |
| 544 | IKDASGEIFVDREMLGIR-IKSNSWFLFK-a2-b2 | MCM21 | MCM21 | 189 | 229 | 691,973 | 5 | -1,6 | 9 | 4 | 26,74 |
| 545 | IKDASGEIFVDREMLGIR-IKSNSWFLFK-a2-b2 | MCM21 | MCM21 | 189 | 229 | 691,974 | 5 | -0,2 | 12 | 2 | 26,71 |
| 546 | TSQFEKPHYVLLK-QIFKDLEAK-a6-b4 | MCM21 | MCM21 | 218 | 286 | 564,514 | 5 | 0,5 | 12 | 10 | 36,6 |
| 547 | TSQFEKPHYVLLK-QIFKDLEAK-a6-b4 | MCM21 | MCM21 | 218 | 286 | 564,514 | 5 | 0 | 13 | 11 | 34,81 |
| 548 | TSQFEKPHYVLLK-QIFKDLEAKK-a6-b4 | MCM21 | MCM21 | 218 | 286 | 590,133 | 5 | -0,2 | 8 | 7 | 31,06 |
| 549 | TSQFEKPHYVLLK-RQIFKDLEAK-a6-b5 | MCM21 | MCM21 | 218 | 286 | 595,734 | 5 | 0,1 | 5 | 4 | 24,04 |
| 550 | TSQFEKPHYVLLK-RQIFKDLEAK-a6-b5 | MCM21 | MCM21 | 218 | 286 | 595,734 | 5 | -0,1 | 7 | 5 | 22,79 |
| 551 | VFLQLVEVQKR-DLEAKK-a10-b5 | MCM21 | MCM21 | 280 | 291 | 550,576 | 4 | 2,1 | 5 | 2 | 35,58 |
| 552 | KIIHDLDLDLESSMVSFFVK-QIFKDLEAK-a1-b4 | MCM21 | MCM21 | 292 | 286 | 713,784 | 5 | -0,2 | 5 | 3 | 28,6 |
| 553 | KIIHDLDLDLESSMVSFFVK-RQIFKDLEAK-a1-b5 | MCM21 | MCM21 | 292 | 286 | 931,004 | 4 | 0,3 | 5 | 2 | 27,49 |
| 554 | LGLKSR-KYSTR-a4-b1 | MIF2 | MIF2 | 9 | 148 | 366,969 | 4 | -1,5 | 5 | 2 | 31,07 |
| 555 | KTGIDVK-LGLKSR-a1-b4 | MIF2 | MIF2 | 12 | 9 | 393,494 | 4 | -1,6 | 8 | 6 | 41,6 |
| 556 | KTGIDVK-LGLKSR-a1-b4 | MIF2 | MIF2 | 12 | 9 | 393,494 | 4 | -0,4 | 8 | 9 | 39,53 |
| 557 | KTGIDVK-KYSTR-a1-b1 | MIF2 | MIF2 | 12 | 148 | 517,963 | 3 | -0,3 | 6 | 3 | 27,4 |
| 558 | KTGIDVK-LSKPTYK-a1-b3 | MIF2 | MIF2 | 12 | 142 | 578,674 | 3 | 0,4 | 6 | 1 | 24,69 |
| 559 | SQDDEVVQSPSGKGDGSRR-KTGIDVK-a13-b1 | MIF2 | MIF2 | 90 | 12 | 726,12 | 4 | 0,7 | 8 | 3 | 33,91 |
| 560 | SQDDEVVQSPSGKGDGSRR-LSKPTYK-a13-b3 | MIF2 | MIF2 | 90 | 142 | 596,304 | 5 | 1,1 | 5 | 3 | 28,3 |
| 561 | LSKPTYK-KYSTR-a3-b1 | MIF2 | MIF2 | 142 | 148 | 543,307 | 3 | 0,3 | 7 | 5 | 35,81 |
| 562 | LSKPTYKR-KYSTR-a7-b1 | MIF2 | MIF2 | 146 | 148 | 446,757 | 4 | -1,8 | 7 | 9 | 35,37 |
| 563 | LSKPTYKR-KYSTR-a7-b1 | MIF2 | MIF2 | 146 | 148 | 446,757 | 4 | -0,8 | 7 | 5 | 33,4 |
| 564 | VKVAPLQYWR-NEKIVYK-a2-b3 | MIF2 | MIF2 | 291 | 302 | 573,33 | 4 | -0,3 | 9 | 4 | 38,43 |
| 565 | VKVAPLQYWR-KTGIDVK-a2-b1 | MIF2 | MIF2 | 291 | 12 | 540,066 | 4 | -1,3 | 6 | 3 | 32,95 |
| 566 | VKVAPLQYWR-KDPNAK-a2-b1 | MIF2 | MIF2 | 291 | 364 | 690,389 | 3 | -1,3 | 5 | 1 | 28,49 |
| 567 | KSNKPVLDIDK-IVYKR-a1-b4 | MIF2 | MIF2 | 308 | 306 | 415,248 | 5 | -0,9 | 7 | 10 | 42,11 |
| 568 | RKSNKPVLDIDK-NEKIVYK-a2-b3 | MIF2 | MIF2 | 308 | 302 | 611,605 | 4 | 1,6 | 10 | 4 | 32,47 |
| 569 | KSNKPVLDIDK-VKVAPLQYWR-a1-b2 | MIF2 | MIF2 | 308 | 291 | 885,174 | 3 | -0,6 | 12 | 1 | 31,78 |
| 570 | KSNKPVLDIDK-NEKIVYK-a1-b3 | MIF2 | MIF2 | 308 | 302 | 763,102 | 3 | -0,5 | 6 | 1 | 25,11 |
| 571 | RKSNKPVLDIDK-NEKIVYK-a5-b3 | MIF2 | MIF2 | 311 | 302 | 611,604 | 4 | 0,7 | 9 | 3 | 36,25 |
| 572 | SNKPVLDIDK-NEKIVYK-a3-b3 | MIF2 | MIF2 | 311 | 302 | 720,403 | 3 | -1,7 | 8 | 3 | 36,13 |
| 573 | KSNKPVLDIDK-NEKIVYK-a4-b3 | MIF2 | MIF2 | 311 | 302 | 572,578 | 4 | -1,3 | 11 | 2 | 35,54 |
| 574 | SNKPVLDIDK-VKVAPLQYWR-a3-b2 | MIF2 | MIF2 | 311 | 291 | 632,109 | 4 | 0,1 | 11 | 4 | 35,03 |
| 575 | SNKPVLDIDK-IVYKR-a3-b4 | MIF2 | MIF2 | 311 | 306 | 486,785 | 4 | -0,7 | 5 | 5 | 33,71 |
| 576 | SNKPVLDIDK-IVYKR-a3-b4 | MIF2 | MIF2 | 311 | 306 | 486,784 | 4 | -1,7 | 4 | 11 | 33,18 |
| 577 | KSNKPVLDIDK-VKVAPLQYWR-a4-b2 | MIF2 | MIF2 | 311 | 291 | 531,507 | 5 | -1,1 | 5 | 4 | 31,83 |
| 578 | KSNKPVLDIDK-NEKIVYK-a4-b3 | MIF2 | MIF2 | 311 | 302 | 572,579 | 4 | 0,6 | 9 | 2 | 30,83 |
| 579 | RKSNKPVLDIDK-NEKIVYK-a5-b3 | MIF2 | MIF2 | 311 | 302 | 489,485 | 5 | 0,2 | 8 | 1 | 29,74 |
| 580 | KKPTPTRPYNYVPTGRPR-KDPNAK-a2-b1 | MIF2 | MIF2 | 343 | 364 | 490,44 | 6 | -1,5 | 8 | 7 | 31,38 |
| 581 | KKPTPTRPYNYVPTGRPR-GRPKKDPNAK-a2-b4 | MIF2 | MIF2 | 343 | 363 | 483,131 | 7 | -2 | 10 | 5 | 27,08 |
| 582 | KKPTPTRPYNYVPTGRPR-GRPKKDPNAK-a2-b5 | MIF2 | MIF2 | 343 | 364 | 483,132 | 7 | -0,6 | 9 | 8 | 26,9 |
| 583 | KKPTPTRPYNYVPTGRPR-GRPKKDPNAK-a2-b5 | MIF2 | MIF2 | 343 | 364 | 483,133 | 7 | 1,4 | 9 | 1 | 23,79 |
| 584 | KKPTPTRPYNYVPTGRPR-KDPNAK-a2-b1 | MIF2 | MIF2 | 343 | 364 | 588,328 | 5 | 1,1 | 5 | 2 | 22,87 |
| 585 | KKPTPTRPYNYVPTGRPR-VKVAPLQYWR-a2-b2 | MIF2 | MIF2 | 343 | 291 | 705,798 | 5 | -1,7 | 6 | 2 | 22,36 |
| 586 | GRPKKDPNAK-VKVAPLQYWR-a4-b2 | MIF2 | MIF2 | 363 | 291 | 627,612 | 4 | 0,5 | 5 | 1 | 24,65 |
| 587 | GRPKKDPNAK-IVYKR-a5-b4 | MIF2 | MIF2 | 364 | 306 | 482,288 | 4 | -1 | 7 | 3 | 31,61 |
| 588 | IESGGIENGEWLKHGILEANVK-VKVAPLQYWR-a13-b2 | MIF2 | MIF2 | 397 | 291 | 948,265 | 4 | 0,6 | 12 | 3 | 25,94 |
| 589 | DTKDENFALEIMFDK-KLSNSFR-a3-b1 | MIF2 | MIF2 | 435 | 467 | 935,464 | 3 | -1,4 | 7 | 4 | 33,05 |
| 590 | HKEYFASGILK-LPAISGQKK-a2-b8 | MIF2 | MIF2 | 449 | 466 | 593,59 | 4 | -1,5 | 11 | 15 | 40,76 |
| 591 | HKEYFASGILK-LPAISGQKK-a2-b8 | MIF2 | MIF2 | 449 | 466 | 791,118 | 3 | 0 | 13 | 9 | 35,26 |
| 592 | HKEYFASGILK-KLSNSFR-a2-b1 | MIF2 | MIF2 | 449 | 467 | 571,065 | 4 | 0,3 | 8 | 4 | 30,58 |
| 593 | KLSNSFR-NKFLSVK-a1-b2 | MIF2 | MIF2 | 467 | 493 | 456,765 | 4 | -0,2 | 6 | 4 | 30,03 |
| 594 | KLSNSFR-NKFLSVK-a1-b2 | MIF2 | MIF2 | 467 | 493 | 608,684 | 3 | -1,1 | 7 | 4 | 27,7 |
| 595 | VNDVELAFKK-NEMLLKR-a9-b6 | MTW1 | MTW1 | 139 | 146 | 551,56 | 4 | -0,2 | 9 | 6 | 37,19 |
| 596 | LNELLKCK-GFKQK-a6-b3 | MTW1 | MTW1 | 170 | 162 | 441,254 | 4 | -1,4 | 5 | 2 | 33,4 |
| 597 | ILESLKPIDDTMTLLTDSLRK-CKDDVQLQK-a6-b2 | MTW1 | MTW1 | 185 | 172 | 918,99 | 4 | -1,7 | 7 | 7 | 27,26 |
| 598 | KLGLLGDKEDEK-QSAKPDAR-a1-b4 | MTW1 | MTW1 | 247 | 262 | 471,657 | 5 | -0,7 | 8 | 2 | 24,76 |
| 599 | EFKEIMEER-LKQVFNR-a3-b2 | NNF1 | NNF1 | 70 | 13 | 563,799 | 4 | -0,9 | 12 | 9 | 38,25 |
| 600 | SSSESDSSILMNVNDIKTLR-LDVAPEAKSTQSK-a17-b8 | OKP1 | OKP1 | 46 | 57 | 927,472 | 4 | -1,4 | 12 | 5 | 32,23 |
| 601 | SSSESDSSILMNVNDIKTLR-LDVAPEAKSTQSKK-a17-b8 | OKP1 | OKP1 | 46 | 57 | 959,495 | 4 | -1,6 | 9 | 2 | 28,53 |
| 602 | LDVAPEAKSTQSKK-TNKEEGQYHHKGSK-a8-b11 | OKP1 | OKP1 | 57 | 92 | 821,175 | 4 | 0,3 | 14 | 5 | 36,79 |
| 603 | TLRLDVAPEAKSTQSK-TNKEEGQYHHKGSK-a11-b11 | OKP1 | OKP1 | 57 | 92 | 705,569 | 5 | 0,1 | 16 | 6 | 36,79 |
| 604 | LDVAPEAKSTQSK-TNKEEGQYHHK-a8-b3 | OKP1 | OKP1 | 57 | 84 | 961,15 | 3 | 0,2 | 12 | 10 | 35,71 |
| 605 | LDVAPEAKSTQSK-KVIQAEYR-a8-b1 | OKP1 | OKP1 | 57 | 132 | 630,094 | 4 | -0,8 | 11 | 4 | 35,41 |
| 606 | LDVAPEAKSTQSKK-FEVGKESTGK-a8-b5 | OKP1 | OKP1 | 57 | 103 | 680,866 | 4 | 1,8 | 12 | 1 | 33,88 |
| 607 | TLRLDVAPEAKSTQSK-TNKEEGQYHHK-a11-b3 | OKP1 | OKP1 | 57 | 84 | 813,671 | 4 | -1,3 | 13 | 4 | 33,83 |
| 608 | LDVAPEAKSTQSK-NYELKHWK-a8-b5 | OKP1 | OKP1 | 57 | 149 | 876,793 | 3 | -1,5 | 9 | 3 | 33,7 |
| 609 | LDVAPEAKSTQSK-KVIQAEYR-a8-b1 | OKP1 | OKP1 | 57 | 132 | 630,094 | 4 | -1,4 | 7 | 4 | 33,58 |
| 610 | LDVAPEAKSTQSKK-GSKQLR-a8-b3 | OKP1 | OKP1 | 57 | 95 | 582,578 | 4 | -1,9 | 8 | 2 | 32,59 |
| 611 | LDVAPEAKSTQSK-TNKEEGQYHHK-a8-b3 | OKP1 | OKP1 | 57 | 84 | 961,152 | 3 | 2,3 | 10 | 5 | 32,56 |
| 612 | LDVAPEAKSTQSKK-TNKEEGQYHHK-a8-b3 | OKP1 | OKP1 | 57 | 84 | 602,712 | 5 | -0,6 | 10 | 1 | 32,32 |
| 613 | TLRLDVAPEAKSTQSK-TNKEEGQYHHK-a11-b3 | OKP1 | OKP1 | 57 | 84 | 651,14 | 5 | 0,3 | 9 | 2 | 31,67 |
| 614 | LDVAPEAKSTQSKK-TNKEEGQYHHK-a8-b3 | OKP1 | OKP1 | 57 | 84 | 1003,846 | 3 | -1,7 | 9 | 7 | 29,81 |
| 615 | LDVAPEAKSTQSK-FEVGKESTGK-a8-b5 | OKP1 | OKP1 | 57 | 103 | 648,84 | 4 | -1,6 | 5 | 4 | 27,56 |
| 616 | TLRLDVAPEAKSTQSK-KVIQAEYR-a11-b1 | OKP1 | OKP1 | 57 | 132 | 722,654 | 4 | 0,6 | 9 | 2 | 27,52 |
| 617 | LDVAPEAKSTQSKK-EEGQYHHKGSK-a8-b8 | OKP1 | OKP1 | 57 | 92 | 735,379 | 4 | 0 | 8 | 1 | 23,77 |
| 618 | KSLFYENSDDAEEGEIEER-LDVAPEAKSTQSK-a1-b8 | OKP1 | OKP1 | 63 | 57 | 1257,598 | 3 | 0 | 10 | 3 | 32,11 |
| 619 | KSLFYENSDDAEEGEIEER-LDVAPEAKSTQSK-a1-b8 | OKP1 | OKP1 | 63 | 57 | 943,452 | 4 | 1,5 | 9 | 4 | 31,17 |
| 620 | KSLFYENSDDAEEGEIEER-LDVAPEAKSTQSK-a1-b8 | OKP1 | OKP1 | 63 | 57 | 943,449 | 4 | -1 | 6 | 3 | 28,55 |
| 621 | KSLFYENSDDAEEGEIEER-TLRLDVAPEAKSTQSK-a1-b11 | OKP1 | OKP1 | 63 | 57 | 1036,01 | 4 | 1,1 | 7 | 3 | 28,48 |
| 622 | KSLFYENSDDAEEGEIEER-TNKEEGQYHHK-a1-b3 | OKP1 | OKP1 | 63 | 84 | 1256,57 | 3 | -0,4 | 5 | 1 | 22,25 |
| 623 | TNKEEGQYHHK-GSKQLR-a3-b3 | OKP1 | OKP1 | 84 | 95 | 549,785 | 4 | 0,1 | 8 | 73 | 35,64 |
| 624 | TNKEEGQYHHK-IYSKK-a3-b4 | OKP1 | OKP1 | 84 | 202 | 537,279 | 4 | 0,1 | 7 | 15 | 34,79 |
| 625 | TNKEEGQYHHK-GSKQLR-a3-b3 | OKP1 | OKP1 | 84 | 95 | 549,785 | 4 | -0,2 | 8 | 19 | 33,9 |
| 626 | TNKEEGQYHHK-KVIQAEYR-a3-b1 | OKP1 | OKP1 | 84 | 132 | 838,765 | 3 | 2,1 | 11 | 12 | 32,57 |
| 627 | TNKEEGQYHHK-IYSKK-a3-b4 | OKP1 | OKP1 | 84 | 202 | 537,279 | 4 | 0,2 | 6 | 2 | 31,72 |
| 628 | TNKEEGQYHHK-NYELKHWK-a3-b5 | OKP1 | OKP1 | 84 | 149 | 657,076 | 4 | -1,6 | 11 | 9 | 31,55 |
| 629 | TNKEEGQYHHK-QAKFPSR-a3-b3 | OKP1 | OKP1 | 84 | 222 | 586,048 | 4 | -0,4 | 9 | 17 | 31,11 |
| 630 | TNKEEGQYHHK-NYELKHWK-a3-b5 | OKP1 | OKP1 | 84 | 149 | 657,078 | 4 | 0,9 | 10 | 2 | 30,42 |
| 631 | TNKEEGQYHHK-HWKKPSK-a3-b3 | OKP1 | OKP1 | 84 | 152 | 605,314 | 4 | 0,2 | 7 | 9 | 29,92 |
| 632 | TNKEEGQYHHKGSK-FEVGKESTGK-a3-b5 | OKP1 | OKP1 | 84 | 103 | 477,742 | 6 | 1,2 | 8 | 3 | 29,4 |
| 633 | TNKEEGQYHHKGSK-FEVGKESTGK-a3-b5 | OKP1 | OKP1 | 84 | 103 | 477,741 | 6 | -0,7 | 8 | 1 | 28,75 |
| 634 | TNKEEGQYHHK-QAKFPSR-a3-b3 | OKP1 | OKP1 | 84 | 222 | 469,04 | 5 | -0,8 | 8 | 6 | 28,35 |
| 635 | TNKEEGQYHHK-STQSKK-a3-b5 | OKP1 | OKP1 | 84 | 62 | 729,367 | 3 | 1 | 5 | 3 | 26,34 |
| 636 | TNKEEGQYHHKGSK-SKELK-a3-b2 | OKP1 | OKP1 | 84 | 368 | 477,65 | 5 | -0,4 | 7 | 1 | 25,82 |
| 637 | TNKEEGQYHHK-IYSKK-a3-b4 | OKP1 | OKP1 | 84 | 202 | 430,024 | 5 | -2,2 | 4 | 1 | 24,24 |
| 638 | TNKEEGQYHHKGSK-NYELKHWK-a3-b5 | OKP1 | OKP1 | 84 | 149 | 580,294 | 5 | 1,7 | 7 | 1 | 23,47 |
| 639 | TNKEEGQYHHKGSK-GSKQLR-a3-b3 | OKP1 | OKP1 | 84 | 95 | 494,459 | 5 | 0,6 | 6 | 2 | 23,16 |
| 640 | TNKEEGQYHHKGSK-FEVGKESTGK-a11-b5 | OKP1 | OKP1 | 92 | 103 | 716,108 | 4 | 0 | 16 | 16 | 38,91 |
| 641 | TNKEEGQYHHKGSK-IYSKK-a11-b4 | OKP1 | OKP1 | 92 | 202 | 605,316 | 4 | -0,2 | 7 | 12 | 38,6 |
| 642 | TNKEEGQYHHKGSK-NYELKHWK-a11-b5 | OKP1 | OKP1 | 92 | 149 | 725,115 | 4 | 0,7 | 13 | 8 | 37,41 |
| 643 | TNKEEGQYHHKGSK-FEVGKESTGK-a11-b5 | OKP1 | OKP1 | 92 | 103 | 716,108 | 4 | 0,8 | 13 | 9 | 36,79 |
| 644 | TNKEEGQYHHKGSK-IYSKK-a11-b4 | OKP1 | OKP1 | 92 | 202 | 484,454 | 5 | -1,2 | 6 | 2 | 34,97 |
| 645 | TNKEEGQYHHKGSK-KVIQAEYR-a11-b1 | OKP1 | OKP1 | 92 | 132 | 697,362 | 4 | 0,4 | 11 | 11 | 34,58 |
| 646 | TNKEEGQYHHKGSK-IYSKK-a11-b4 | OKP1 | OKP1 | 92 | 202 | 806,753 | 3 | 0,3 | 8 | 4 | 33,89 |
| 647 | TNKEEGQYHHKGSK-GSKQLR-a11-b3 | OKP1 | OKP1 | 92 | 95 | 617,822 | 4 | 0,6 | 5 | 3 | 31,52 |
| 648 | EEGQYHHKGSK-FEVGKESTGK-a8-b5 | OKP1 | OKP1 | 92 | 103 | 840,079 | 3 | -0,4 | 10 | 4 | 30,97 |
| 649 | EEGQYHHKGSK-FEVGKESTGK-a8-b5 | OKP1 | OKP1 | 92 | 103 | 840,08 | 3 | 1,1 | 10 | 3 | 30,82 |
| 650 | TNKEEGQYHHKGSK-RIYSKK-a11-b5 | OKP1 | OKP1 | 92 | 202 | 858,784 | 3 | -2 | 4 | 18 | 30,24 |
| 651 | TNKEEGQYHHKGSK-TNKEEGQYHHK-a11-b3 | OKP1 | OKP1 | 92 | 84 | 630,906 | 5 | 0,4 | 8 | 5 | 29,91 |
| 652 | TNKEEGQYHHKGSK-SKELK-a11-b2 | OKP1 | OKP1 | 92 | 368 | 477,65 | 5 | -1,6 | 7 | 1 | 29,26 |
| 653 | TNKEEGQYHHKGSK-FEVGKESTGK-a11-b5 | OKP1 | OKP1 | 92 | 103 | 716,108 | 4 | 0,3 | 10 | 2 | 29,11 |
| 654 | TNKEEGQYHHKGSK-KVIQAEYR-a11-b1 | OKP1 | OKP1 | 92 | 132 | 697,363 | 4 | 1,8 | 8 | 1 | 28,47 |
| 655 | EEGQYHHKGSK-KVIQAEYR-a8-b1 | OKP1 | OKP1 | 92 | 132 | 815,086 | 3 | 2,1 | 8 | 5 | 28,34 |
| 656 | TNKEEGQYHHKGSK-RLTEKLIQK-a11-b5 | OKP1 | OKP1 | 92 | 290 | 582,519 | 5 | -0,4 | 10 | 2 | 27,92 |
| 657 | TNKEEGQYHHKGSK-QAKFPSR-a11-b3 | OKP1 | OKP1 | 92 | 222 | 523,47 | 5 | 0 | 7 | 2 | 27,82 |
| 658 | TNKEEGQYHHKGSK-KVIQAEYR-a11-b1 | OKP1 | OKP1 | 92 | 132 | 558,09 | 5 | -2,1 | 6 | 2 | 25,3 |
| 659 | EEGQYHHKGSK-IYSKK-a8-b4 | OKP1 | OKP1 | 92 | 202 | 519,521 | 4 | 2,3 | 5 | 2 | 22,02 |
| 660 | GSKQLR-RIYSKK-a3-b5 | OKP1 | OKP1 | 95 | 202 | 540,658 | 3 | -0,5 | 9 | 4 | 35,86 |
| 661 | GSKQLR-RIYSKK-a3-b5 | OKP1 | OKP1 | 95 | 202 | 540,657 | 3 | -2,6 | 8 | 3 | 31,84 |
| 662 | GSKQLR-IYSKK-a3-b4 | OKP1 | OKP1 | 95 | 202 | 488,624 | 3 | -1,3 | 7 | 4 | 31,74 |
| 663 | GSKQLR-STQSKK-a3-b5 | OKP1 | OKP1 | 95 | 62 | 376,718 | 4 | -1,6 | 5 | 2 | 27,35 |
| 664 | GSKQLR-STQSKK-a3-b5 | OKP1 | OKP1 | 95 | 62 | 376,718 | 4 | 0 | 5 | 2 | 24,07 |
| 665 | FEVGKESTGK-IYSKK-a5-b4 | OKP1 | OKP1 | 103 | 202 | 619,672 | 3 | -0,8 | 8 | 3 | 41,98 |
| 666 | FEVGKESTGK-NYELKHWK-a5-b5 | OKP1 | OKP1 | 103 | 149 | 584,803 | 4 | -1,2 | 12 | 8 | 41,85 |
| 667 | FEVGKESTGK-NYELKHWK-a5-b5 | OKP1 | OKP1 | 103 | 149 | 584,805 | 4 | 2,2 | 12 | 8 | 40,93 |
| 668 | FEVGKESTGK-LLEIILTKIK-a5-b8 | OKP1 | OKP1 | 103 | 213 | 601,36 | 4 | 0,3 | 11 | 3 | 39,75 |
| 669 | FEVGKESTGK-KVIQAEYR-a5-b1 | OKP1 | OKP1 | 103 | 132 | 742,399 | 3 | -0,6 | 12 | 6 | 39,62 |
| 670 | FEVGKESTGK-GSKQLR-a5-b3 | OKP1 | OKP1 | 103 | 95 | 477,511 | 4 | -1,7 | 8 | 3 | 37,1 |
| 671 | FEVGKESTGK-IYSKK-a5-b4 | OKP1 | OKP1 | 103 | 202 | 465,006 | 4 | -0,7 | 6 | 1 | 36,62 |
| 672 | FEVGKESTGK-QAKFPSR-a5-b3 | OKP1 | OKP1 | 103 | 222 | 513,775 | 4 | 0,9 | 9 | 14 | 36,12 |
| 673 | FEVGKESTGK-GSKQLR-a5-b3 | OKP1 | OKP1 | 103 | 95 | 477,512 | 4 | -0,2 | 7 | 5 | 35,83 |
| 674 | FEVGKESTGK-RIYSKK-a5-b5 | OKP1 | OKP1 | 103 | 202 | 504,031 | 4 | -1,4 | 4 | 15 | 35,83 |
| 675 | FEVGKESTGK-KVIQAEYR-a5-b1 | OKP1 | OKP1 | 103 | 132 | 557,051 | 4 | 0 | 9 | 8 | 35,78 |
| 676 | FEVGKESTGK-STQSKK-a5-b5 | OKP1 | OKP1 | 103 | 62 | 633,003 | 3 | 0,5 | 5 | 2 | 32 |
| 677 | FEVGKESTGK-KVIQAEYR-a5-b1 | OKP1 | OKP1 | 103 | 132 | 557,051 | 4 | -0,3 | 7 | 2 | 27,77 |
| 678 | FEVGKESTGK-QAKFPSR-a5-b3 | OKP1 | OKP1 | 103 | 222 | 513,774 | 4 | -1,8 | 6 | 4 | 23,86 |
| 679 | ESTGKLQSHLSDGSATSGEGNVRPWEFR-KVIQAEYR-a5-b1 | OKP1 | OKP1 | 108 | 132 | 1044,777 | 4 | 2,5 | 9 | 2 | 25,59 |
| 680 | ESTGKLQSHLSDGSATSGEGNVRPWEFR-KVIQAEYR-a5-b1 | OKP1 | OKP1 | 108 | 132 | 1044,775 | 4 | 0,1 | 7 | 3 | 23,31 |
| 681 | ESTGKLQSHLSDGSATSGEGNVRPWEFR-LLEIILTKIK-a5-b8 | OKP1 | OKP1 | 108 | 213 | 871,468 | 5 | 0,3 | 8 | 1 | 23,14 |
| 682 | ESTGKLQSHLSDGSATSGEGNVRPWEFR-GSKQLR-a5-b3 | OKP1 | OKP1 | 108 | 95 | 965,235 | 4 | -0,2 | 6 | 3 | 22,91 |
| 683 | KVIQAEYR-KVIQAEYR-a1-b1 | OKP1 | OKP1 | 132 | 132 | 538,305 | 4 | -0,7 | 13 | 4 | 37,53 |
| 684 | KVIQAEYR-GSKQLR-a1-b3 | OKP1 | OKP1 | 132 | 95 | 458,766 | 4 | 0,1 | 5 | 6 | 36,94 |
| 685 | KVIQAEYR-NYELKHWK-a1-b5 | OKP1 | OKP1 | 132 | 149 | 754,407 | 3 | -1 | 11 | 5 | 35,42 |
| 686 | KVIQAEYR-QAKFPSR-a1-b3 | OKP1 | OKP1 | 132 | 222 | 495,028 | 4 | -1,4 | 6 | 3 | 33,95 |
| 687 | KVIQAEYR-QAKFPSR-a1-b3 | OKP1 | OKP1 | 132 | 222 | 659,702 | 3 | -0,7 | 8 | 20 | 32,6 |
| 688 | KVIQAEYR-QAKFPSR-a1-b3 | OKP1 | OKP1 | 132 | 222 | 495,03 | 4 | 1,6 | 6 | 13 | 32,51 |
| 689 | KVIQAEYR-IYSKK-a1-b4 | OKP1 | OKP1 | 132 | 202 | 594,676 | 3 | -1,7 | 6 | 2 | 32,38 |
| 690 | KVIQAEYR-NYELKHWK-a1-b5 | OKP1 | OKP1 | 132 | 149 | 754,406 | 3 | -2,5 | 10 | 4 | 30,47 |
| 691 | KVIQAEYR-SKELK-a1-b2 | OKP1 | OKP1 | 132 | 368 | 583,337 | 3 | -0,5 | 4 | 3 | 24,89 |
| 692 | NYELKHWK-IYSKK-a5-b4 | OKP1 | OKP1 | 149 | 202 | 631,681 | 3 | -0,5 | 7 | 4 | 37,01 |
| 693 | NYELKHWK-QAKFPSR-a5-b3 | OKP1 | OKP1 | 149 | 222 | 418,427 | 5 | -0,1 | 10 | 7 | 34,58 |
| 694 | NYELKHWK-GSKQLR-a5-b3 | OKP1 | OKP1 | 149 | 95 | 486,519 | 4 | 1,3 | 7 | 6 | 29,21 |
| 695 | NYELKHWK-QAKFPSR-a5-b3 | OKP1 | OKP1 | 149 | 222 | 696,706 | 3 | 0,2 | 6 | 6 | 27,87 |
| 696 | NYELKHWK-STQSKK-a5-b5 | OKP1 | OKP1 | 149 | 62 | 484,01 | 4 | -0,2 | 6 | 2 | 27,63 |
| 697 | HWKKPSK-QAKFPSR-a3-b3 | OKP1 | OKP1 | 152 | 222 | 471,018 | 4 | 0,3 | 5 | 3 | 29,34 |
| 698 | KPSKIMIGSILR-GSKQLR-a4-b3 | OKP1 | OKP1 | 156 | 95 | 542,829 | 4 | -2,1 | 6 | 2 | 32,23 |
| 699 | KPSKIMIGSILR-NYELKHWK-a4-b5 | OKP1 | OKP1 | 156 | 149 | 650,121 | 4 | -0,7 | 10 | 2 | 31,83 |
| 700 | YEKEMNQMTHGDNNEVK-TNKEEGQYHHK-a3-b3 | OKP1 | OKP1 | 183 | 84 | 894,406 | 4 | 0,5 | 11 | 1 | 24,76 |
| 701 | EMNQMTHGDNNEVKR-KVIQAEYR-a14-b1 | OKP1 | OKP1 | 197 | 132 | 737,36 | 4 | -1,6 | 12 | 5 | 31,48 |
| 702 | EMNQMTHGDNNEVKR-IYSKK-a14-b4 | OKP1 | OKP1 | 197 | 202 | 860,086 | 3 | 0,9 | 7 | 9 | 30,74 |
| 703 | EMNQMTHGDNNEVKR-NYELKHWK-a14-b5 | OKP1 | OKP1 | 197 | 149 | 765,112 | 4 | -1,8 | 11 | 3 | 30 |
| 704 | EMNQMTHGDNNEVKR-TNKEEGQYHHK-a14-b3 | OKP1 | OKP1 | 197 | 84 | 828,38 | 4 | -0,3 | 11 | 9 | 28,81 |
| 705 | EMNQMTHGDNNEVKR-FEVGKESTGK-a14-b5 | OKP1 | OKP1 | 197 | 103 | 756,107 | 4 | -0,1 | 10 | 2 | 28,34 |
| 706 | EMNQMTHGDNNEVKR-IYSKK-a14-b4 | OKP1 | OKP1 | 197 | 202 | 860,085 | 3 | 0,2 | 6 | 19 | 27,16 |
| 707 | EMNQMTHGDNNEVKR-KVIQAEYR-a14-b1 | OKP1 | OKP1 | 197 | 132 | 737,362 | 4 | 1,4 | 6 | 2 | 24,41 |
| 708 | EMNQMTHGDNNEVKR-IYSKK-a14-b4 | OKP1 | OKP1 | 197 | 202 | 860,085 | 3 | 0,2 | 6 | 2 | 24,01 |
| 709 | EMNQMTHGDNNEVKR-QAKFPSR-a14-b3 | OKP1 | OKP1 | 197 | 222 | 555,469 | 5 | -0,2 | 6 | 9 | 23,71 |
| 710 | RIYSKK-SKELK-a5-b2 | OKP1 | OKP1 | 202 | 368 | 512,644 | 3 | 0,2 | 6 | 4 | 27,96 |
| 711 | RIYSKK-STQSKK-a5-b5 | OKP1 | OKP1 | 202 | 62 | 537,314 | 3 | -0,4 | 5 | 1 | 27,31 |
| 712 | ERLLEIILTKIK-FEVGKESTGK-a10-b5 | OKP1 | OKP1 | 213 | 103 | 672,647 | 4 | 1,4 | 14 | 2 | 40,58 |
| 713 | ERLLEIILTKIK-IYSKK-a10-b4 | OKP1 | OKP1 | 213 | 202 | 561,855 | 4 | 0,6 | 5 | 2 | 37,44 |
| 714 | LLEIILTKIK-IYSKK-a8-b4 | OKP1 | OKP1 | 213 | 202 | 653,756 | 3 | 0,1 | 6 | 3 | 35,67 |
| 715 | LLEIILTKIK-GSKQLR-a8-b3 | OKP1 | OKP1 | 213 | 95 | 670,43 | 3 | 0,1 | 5 | 2 | 34,68 |
| 716 | ERLLEIILTKIK-GSKQLR-a10-b3 | OKP1 | OKP1 | 213 | 95 | 574,361 | 4 | 0,7 | 6 | 4 | 33,81 |
| 717 | LLEIILTKIK-NYELKHWK-a8-b5 | OKP1 | OKP1 | 213 | 149 | 610,366 | 4 | -0,9 | 7 | 2 | 33,07 |
| 718 | QAKFPSR-GSKQLR-a3-b3 | OKP1 | OKP1 | 222 | 95 | 415,49 | 4 | 0,3 | 5 | 3 | 26,63 |
| 719 | DLDIEYIYSKR-TNKEEGQYHHK-a10-b3 | OKP1 | OKP1 | 240 | 84 | 731,362 | 4 | -1 | 7 | 2 | 28,03 |
| 720 | DLDIEYIYSKR-QAKFPSR-a10-b3 | OKP1 | OKP1 | 240 | 222 | 795,753 | 3 | -0,3 | 6 | 1 | 25,05 |
| 721 | LCMNLKTNNK-LTEKLIQK-a6-b4 | OKP1 | OKP1 | 280 | 290 | 587,079 | 4 | -0,2 | 12 | 8 | 39,61 |
| 722 | LCMNLKTNNK-RLTEKLIQK-a6-b5 | OKP1 | OKP1 | 280 | 290 | 626,104 | 4 | -0,4 | 8 | 3 | 34,39 |
| 723 | LCMNLKTNNK-RLTEKLIQK-a6-b5 | OKP1 | OKP1 | 280 | 290 | 626,105 | 4 | 0,7 | 6 | 1 | 28,4 |
| 724 | RLTEKLIQK-TNNKK-a5-b4 | OKP1 | OKP1 | 290 | 284 | 624,043 | 3 | 1,2 | 9 | 15 | 38,64 |
| 725 | RLTEKLIQK-SKELK-a5-b2 | OKP1 | OKP1 | 290 | 368 | 468,291 | 4 | 0,4 | 9 | 4 | 37,99 |
| 726 | RLTEKLIQK-KLCMNLK-a5-b1 | OKP1 | OKP1 | 290 | 274 | 724,759 | 3 | -0,5 | 9 | 4 | 33,45 |
| 727 | LTEKLIQK-KLCMNLK-a4-b1 | OKP1 | OKP1 | 290 | 274 | 504,796 | 4 | 0,6 | 9 | 4 | 32,34 |
| 728 | RLTEKLIQK-SKELK-a5-b2 | OKP1 | OKP1 | 290 | 368 | 468,29 | 4 | -1,6 | 6 | 1 | 31,01 |
| 729 | LTEKLIQK-TNNKKR-a4-b5 | OKP1 | OKP1 | 290 | 285 | 468,284 | 4 | 0,2 | 5 | 1 | 30,8 |
| 730 | RLTEKLIQK-KLCMNLK-a5-b1 | OKP1 | OKP1 | 290 | 274 | 724,757 | 3 | -2,3 | 8 | 3 | 30,77 |
| 731 | LTEKLIQK-TNNKKR-a4-b4 | OKP1 | OKP1 | 290 | 284 | 624,043 | 3 | 0,8 | 6 | 3 | 29,82 |
| 732 | LIQKDLHPVLNK-LCMNLKTNNK-a4-b6 | OKP1 | OKP1 | 294 | 280 | 558,914 | 5 | -0,4 | 14 | 6 | 39,73 |
| 733 | LIQKDLHPVLNK-SKELK-a4-b2 | OKP1 | OKP1 | 294 | 368 | 540,575 | 4 | -1,6 | 6 | 1 | 35,96 |
| 734 | LIQKDLHPVLNK-KLCMNLK-a4-b1 | OKP1 | OKP1 | 294 | 274 | 616,106 | 4 | -0,9 | 10 | 4 | 35 |
| 735 | NDSHELNLMLNDPIKSTADVR-LDKEEVLSLLPSLK-a15-b3 | OKP1 | OKP1 | 341 | 350 | 1026,545 | 4 | -0,9 | 17 | 4 | 36,15 |
| 736 | NDSHELNLMLNDPIKSTADVR-LDKEEVLSLLPSLK-a15-b3 | OKP1 | OKP1 | 341 | 350 | 821,438 | 5 | -0,4 | 13 | 21 | 35,17 |
| 737 | NDSHELNLMLNDPIKSTADVR-LIQKDLHPVLNK-a15-b4 | OKP1 | OKP1 | 341 | 294 | 788,224 | 5 | 0,6 | 5 | 2 | 25,47 |
| 738 | LDKEEVLSLLPSLK-LTEKLIQK-a3-b4 | OKP1 | OKP1 | 350 | 290 | 674,156 | 4 | 1,6 | 5 | 1 | 27,43 |
| 739 | EYTKK-SKELK-a4-b2 | OKP1 | OKP1 | 365 | 368 | 470,602 | 3 | 0,2 | 6 | 4 | 35,3 |
| 740 | ELKETMGQMISDSHEEEIK-LDKEEVLSLLPSLK-a3-b3 | OKP1 | OKP1 | 371 | 350 | 989,511 | 4 | 0,7 | 12 | 2 | 27,64 |
| 741 | ELKETMGQMISDSHEEEIK-LDKEEVLSLLPSLKEYTK-a3-b14 | OKP1 | OKP1 | 371 | 361 | 1119,823 | 4 | 0,6 | 13 | 4 | 25,52 |
| 742 | ELKETMGQMISDSHEEEIK-LDKEEVLSLLPSLK-a3-b3 | OKP1 | OKP1 | 371 | 350 | 989,508 | 4 | -2,1 | 9 | 4 | 24,36 |
| 743 | EVFVPHHESHQDKTEEDIH-KVIQAEYR-a13-b1 | OKP1 | OKP1 | 400 | 132 | 864,928 | 4 | 2 | 10 | 8 | 27,44 |
| 744 | EVFVPHHESHQDKTEEDIH-KSKELK-a13-b3 | OKP1 | OKP1 | 400 | 368 | 796,4 | 4 | 0,6 | 6 | 7 | 25,75 |
