# Supplementary material for: The COMA complex interacts with Cse4 and positions Sli15/Ipl1 at the budding yeast inner kinetochore
Source: eLife. 2019 May 21;8:e42879. doi: 10.7554/eLife.42879 (PMC6546395; doi:10.7554/eLife.42879)
Supplement: Supplementary file 2. [file elife-42879-supp2.docx]

**Supplementary file 2. Inter- and intra-protein cross-links detected on *in vitro* reconstituted Sli15/Ipl1 interacting with the inner kinetochore proteins Ctf19, Okp1, Ame1 and Mcm21 (COMA).**

COMA and Sli15/Ipl1 complexes were recombinantly expressed in insect cells and affinity-purified via Ame1-6xFLAG-6xHis, Mcm21-6xFLAG-6xHis, or Sli15-2xStrep, respectively. A nearly stoichiometric supramolecular complex of COMA and Sli15/Ipl1 was generated *in vitro* and chemical cross-links introduced by BS3 were identified as described (see materials and methods). In total, 98 inter-protein (marked in dark blue) and 69 intra-protein (marked in light blue) cross-links were detected on the recombinant complex (for a graphical representation of the cross-link network see Figure 4A).

| **No** | **Topology** | **Protein1** | **Protein2** | **AbsPos1** | **AbsPos2** | **Mz** | **z** | **Error** | **mions** | **Nseen** | **ld.Score** |
| --- | --- | --- | --- | --- | --- | --- | --- | --- | --- | --- | --- |
| 1 | DFNKSDDDQFRK-AKQLLATR-a4-b2 | Ame1 | CTF19 | 160 | 229 | 638.832 | 4 | -1.9 | 5 | 1 | 25.64 |
| 2 | KEDLNQQIISVR-AKQLLATR-a1-b2 | Ame1 | CTF19 | 213 | 229 | 620.86 | 4 | -2.1 | 8 | 2 | 32.39 |
| 3 | KEDLNQQIISVR-ISIALWKGGER-a1-b7 | Ame1 | CTF19 | 213 | 327 | 703.146 | 4 | 0.5 | 7 | 2 | 30.98 |
| 4 | KEDLNQQIISVR-AKQLLATR-a1-b2 | Ame1 | CTF19 | 213 | 229 | 827.477 | 3 | -1.5 | 7 | 2 | 30.53 |
| 5 | AGKDWHDLQNEQAK-AKQLLATR-a3-b2 | Ame1 | CTF19 | 234 | 229 | 670.107 | 4 | -0.2 | 8 | 1 | 27.79 |
| 6 | LNDLTSTLLGKYEGDRK-ISIALWKGGER-a11-b7 | Ame1 | CTF19 | 266 | 327 | 658.762 | 5 | 0.1 | 12 | 1 | 32.65 |
| 7 | LNDLTSTLLGKYEGDRK-ISIALWKGGER-a11-b7 | Ame1 | CTF19 | 266 | 327 | 658.761 | 5 | -2.2 | 11 | 2 | 31.72 |
| 8 | DFNKSDDDQFR-IYSKK-a4-b4 | Ame1 | OKP1 | 160 | 202 | 721.355 | 3 | 3.9 | 6 | 4 | 34.61 |
| 9 | DFNKSDDDQFR-RIYSKK-a4-b5 | Ame1 | OKP1 | 160 | 202 | 773.386 | 3 | 0.9 | 4 | 2 | 29.45 |
| 10 | DFNKSDDDQFRK-STQSKK-a4-b5 | Ame1 | OKP1 | 160 | 62 | 583.288 | 4 | 1.6 | 6 | 1 | 26.86 |
| 11 | DFNKSDDDQFRK-GSKQLR-a4-b3 | Ame1 | OKP1 | 160 | 95 | 585.795 | 4 | -0.2 | 5 | 1 | 24.51 |
| 12 | DFNKSDDDQFRK-IYSKK-a4-b4 | Ame1 | OKP1 | 160 | 202 | 573.29 | 4 | 0.8 | 4 | 2 | 24.42 |
| 13 | LLYKLDLR-STQSKK-a4-b5 | Ame1 | OKP1 | 172 | 62 | 463.026 | 4 | -0.1 | 6 | 3 | 32.66 |
| 14 | NEIQELKAGK-LTEKLIQK-a7-b4 | Ame1 | OKP1 | 231 | 290 | 560.579 | 4 | 1 | 12 | 4 | 39.25 |
| 15 | NEIQELKAGK-RLTEKLIQK-a7-b5 | Ame1 | OKP1 | 231 | 290 | 799.136 | 3 | -0.5 | 8 | 4 | 28.57 |
| 16 | AGKDWHDLQNEQAK-LCMNLKTNNK-a3-b6 | Ame1 | OKP1 | 234 | 280 | 753.874 | 4 | 2.2 | 10 | 2 | 30.57 |
| 17 | AGKDWHDLQNEQAK-LTEKLIQK-a3-b4 | Ame1 | OKP1 | 234 | 290 | 688.119 | 4 | -0.3 | 8 | 2 | 27.55 |
| 18 | LNDKVK-TNNKK-a4-b4 | Ame1 | OKP1 | 249 | 284 | 365.214 | 4 | 0.6 | 6 | 2 | 35.76 |
| 19 | LQEEKDALLTR-NEIQELKAGK-a5-b7 | CTF19 | Ame1 | 61 | 231 | 646.358 | 4 | 1.9 | 14 | 3 | 40.12 |
| 20 | AKQLLATR-LLYKLDLR-a2-b4 | CTF19 | Ame1 | 229 | 172 | 518.573 | 4 | 1.4 | 9 | 4 | 39.25 |
| 21 | KTGIFQNLINLLK-NEIQELKAGK-a1-b7 | CTF19 | Ame1 | 254 | 231 | 923.539 | 3 | 3.1 | 12 | 1 | 34.11 |
| 22 | TGIFQNLINLLKR-KEDLNQQIISVR-a12-b1 | CTF19 | Ame1 | 266 | 213 | 778.199 | 4 | -0.7 | 13 | 2 | 34.9 |
| 23 | TGIFQNLINLLKR-NEIQELKAGK-a12-b7 | CTF19 | Ame1 | 266 | 231 | 699.907 | 4 | 1.4 | 10 | 2 | 33.67 |
| 24 | TGIFQNLINLLKR-DFNKSDDDQFR-a12-b4 | CTF19 | Ame1 | 266 | 160 | 764.148 | 4 | -0.8 | 12 | 1 | 32.17 |
| 25 | EYGVKTGLK-LLYKLDLR-a5-b4 | CTF19 | Ame1 | 351 | 172 | 542.071 | 4 | 1.2 | 7 | 4 | 31.24 |
| 26 | LQEEKDALLTR-IKSNSWFLFK-a5-b2 | CTF19 | MCM21 | 61 | 229 | 681.375 | 4 | -1.7 | 7 | 1 | 26.33 |
| 27 | INGLTNLQKELVTK-IKDASGEIFVDR-a9-b2 | CTF19 | MCM21 | 136 | 189 | 765.174 | 4 | -3.2 | 11 | 1 | 28.9 |
| 28 | NFQKCLLSLYEFDK-RVFLQLVEVQKR-a4-b11 | CTF19 | MCM21 | 239 | 280 | 692.18 | 5 | -0.8 | 15 | 5 | 37.79 |
| 29 | NFQKCLLSLYEFDK-VFLQLVEVQKR-a4-b10 | CTF19 | MCM21 | 239 | 280 | 825.946 | 4 | -3.7 | 14 | 31 | 36.99 |
| 30 | NFQKCLLSLYEFDK-VFLQLVEVQKRR-a4-b10 | CTF19 | MCM21 | 239 | 280 | 692.18 | 5 | -1.4 | 13 | 3 | 32.9 |
| 31 | NFQKCLLSLYEFDK-VFLQLVEVQKR-a4-b10 | CTF19 | MCM21 | 239 | 280 | 1.100.928 | 3 | -0.5 | 13 | 3 | 30.97 |
| 32 | TGIFQNLINLLKR-QIFKDLEAK-a12-b4 | CTF19 | MCM21 | 266 | 286 | 690.404 | 4 | 2 | 7 | 1 | 22.27 |
| 33 | TGLKEICNVCLFPDMYAR-AQADIPATPIPYEPKK-a4-b15 | CTF19 | MCM21 | 355 | 95 | 1.016.512 | 4 | -3.2 | 10 | 2 | 24.71 |
| 34 | AKQLLATR-EYTKK-a2-b4 | CTF19 | OKP1 | 229 | 365 | 569.333 | 3 | -0.3 | 4 | 2 | 25.78 |
| 35 | TGIFQNLINLLKR-LTEKLIQK-a12-b4 | CTF19 | OKP1 | 266 | 290 | 660.653 | 4 | 0.6 | 8 | 2 | 31.47 |
| 36 | TGIFQNLINLLKR-KVIQAEYRER-a12-b1 | CTF19 | OKP1 | 266 | 132 | 740.427 | 4 | -2.1 | 6 | 2 | 23.9 |
| 37 | AKQLLATR-SFENKISR-a2-b5 | CTF19 | SLI15 | 229 | 315 | 505.289 | 4 | -4 | 9 | 2 | 36.99 |
| 38 | AKQLLATR-DIFSVKNK-a2-b6 | CTF19 | SLI15 | 229 | 365 | 663.39 | 3 | -0.4 | 7 | 1 | 24.7 |
| 39 | TGIFQNLINLLKR-DIFSVKNK-a12-b6 | CTF19 | SLI15 | 266 | 365 | 655.132 | 4 | -1.6 | 9 | 2 | 36.76 |
| 40 | TGIFQNLINLLKR-DIFSVKNK-a12-b6 | CTF19 | SLI15 | 266 | 365 | 655.133 | 4 | -0.5 | 6 | 2 | 32.84 |
| 41 | TGIFQNLINLLKR-FDKTSLK-a12-b3 | CTF19 | SLI15 | 266 | 389 | 627.116 | 4 | -1.8 | 4 | 1 | 31.67 |
| 42 | EYGVKTGLK-LKIKEK-a5-b2 | CTF19 | SLI15 | 351 | 479 | 473.29 | 4 | 1.8 | 4 | 1 | 24.48 |
| 43 | KNIIHR-LNDKVK-a1-b4 | IPL1 | Ame1 | 221 | 249 | 409.25 | 4 | -0.2 | 7 | 1 | 31.59 |
| 44 | TSQFEKPHYVLLK-LLYKLDLR-a6-b4 | MCM21 | Ame1 | 218 | 172 | 552.92 | 5 | -1.1 | 11 | 4 | 37.37 |
| 45 | VELFVKQNEIVSCSILDDIHDFSQNNK-NEIQELKAGK-a6-b7 | MCM21 | Ame1 | 320 | 231 | 1.115.318 | 4 | -0.2 | 9 | 2 | 22.85 |
| 46 | AQADIPATPIPYEPKK-LQEEKDALLTR-a15-b5 | MCM21 | CTF19 | 95 | 61 | 798.683 | 4 | -3.5 | 8 | 1 | 28.24 |
| 47 | AQADIPATPIPYEPKK-TGIFQNLINLLKR-a15-b12 | MCM21 | CTF19 | 95 | 266 | 852.236 | 4 | 1.9 | 10 | 4 | 27.42 |
| 48 | AQADIPATPIPYEPKK-TGIFQNLINLLKR-a15-b12 | MCM21 | CTF19 | 95 | 266 | 852.234 | 4 | -0.2 | 7 | 1 | 26.45 |
| 49 | AQADIPATPIPYEPKKR-TGIFQNLINLLKR-a15-b12 | MCM21 | CTF19 | 95 | 266 | 891.259 | 4 | -0.8 | 9 | 3 | 24.52 |
| 50 | AQADIPATPIPYEPKK-TGIFQNLINLLKR-a15-b12 | MCM21 | CTF19 | 95 | 266 | 852.234 | 4 | -1 | 7 | 1 | 23.37 |
| 51 | AQADIPATPIPYEPKKR-TGIFQNLINLLKR-a15-b12 | MCM21 | CTF19 | 95 | 266 | 713.209 | 5 | -0.7 | 7 | 1 | 23.14 |
| 52 | AQADIPATPIPYEPKKR-TGIFQNLINLLKR-a16-b12 | MCM21 | CTF19 | 96 | 266 | 713.208 | 5 | -2.4 | 10 | 1 | 33.1 |
| 53 | AKLENEEILPEQEWVLK-AKQLLATR-a2-b2 | MCM21 | CTF19 | 99 | 229 | 777.185 | 4 | -1.1 | 5 | 2 | 27.45 |
| 54 | LKIDDISTSDRSELEDYIVLENVYR-AKQLLATR-a2-b2 | MCM21 | CTF19 | 148 | 229 | 1.006.537 | 4 | -1.2 | 9 | 1 | 32.91 |
| 55 | IKSNSWFLFK-AKQLLATR-a2-b2 | MCM21 | CTF19 | 229 | 229 | 577.587 | 4 | 0 | 8 | 3 | 32.56 |
| 56 | IKSNSWFLFK-AKQLLATR-a2-b2 | MCM21 | CTF19 | 229 | 229 | 577.585 | 4 | -3.5 | 5 | 1 | 25.16 |
| 57 | QIFKDLEAK-ENNSKTK-a4-b5 | MCM21 | CTF19 | 286 | 89 | 683.702 | 3 | 1.9 | 8 | 2 | 29.27 |
| 58 | IKSNSWFLFK-TVKESPIAFK-a2-b3 | MCM21 | SLI15 | 229 | 266 | 632.354 | 4 | -2.9 | 10 | 2 | 29.77 |
| 59 | IKSNSWFLFK-KSSIPR-a2-b1 | MCM21 | SLI15 | 229 | 381 | 524.299 | 4 | -0.7 | 4 | 1 | 29.25 |
| 60 | RIKSNSWFLFK-KSSIPR-a3-b1 | MCM21 | SLI15 | 229 | 381 | 563.327 | 4 | 2.8 | 4 | 1 | 24.14 |
| 61 | VELFVKQNEIVSCSILDDIHDFSQNNK-KSSIPR-a6-b1 | MCM21 | SLI15 | 320 | 381 | 804.013 | 5 | -3.1 | 4 | 1 | 27.78 |
| 62 | LDVAPEAKSTQSK-LLYKLDLR-a8-b4 | OKP1 | Ame1 | 57 | 172 | 636.863 | 4 | 0 | 8 | 1 | 31.7 |
| 63 | TNKEEGQYHHK-LLYKLDLR-a3-b4 | OKP1 | Ame1 | 84 | 172 | 636.095 | 4 | 3.9 | 6 | 2 | 26.3 |
| 64 | FEVGKESTGK-LLYKLDLR-a5-b4 | OKP1 | Ame1 | 103 | 172 | 563.82 | 4 | 0.9 | 10 | 5 | 36.76 |
| 65 | ESTGKLQSHLSDGSATSGEGNVRPWEFR-LLYKLDLR-a5-b4 | OKP1 | Ame1 | 108 | 172 | 841.437 | 5 | 1.4 | 10 | 1 | 28.74 |
| 66 | KVIQAEYR-LLYKLDLR-a1-b4 | OKP1 | Ame1 | 132 | 172 | 545.073 | 4 | 0 | 11 | 1 | 35.7 |
| 67 | LLETNTVSALDSVFEKYEK-LLYKLDLR-a16-b4 | OKP1 | Ame1 | 180 | 172 | 839.962 | 4 | -0.2 | 7 | 6 | 32.1 |
| 68 | LLETNTVSALDSVFEKYEK-LLYKLDLR-a16-b4 | OKP1 | Ame1 | 180 | 172 | 839.959 | 4 | -3.3 | 8 | 11 | 31.36 |
| 69 | LLETNTVSALDSVFEKYEK-LLYKLDLR-a16-b4 | OKP1 | Ame1 | 180 | 172 | 839.962 | 4 | 0.6 | 8 | 5 | 30.47 |
| 70 | YEKEMNQMTHGDNNEVK-LLYKLDLR-a3-b4 | OKP1 | Ame1 | 183 | 172 | 810.156 | 4 | 3 | 11 | 9 | 31.49 |
| 71 | YEKEMNQMTHGDNNEVK-LLYKLDLR-a3-b4 | OKP1 | Ame1 | 183 | 172 | 648.325 | 5 | 1.1 | 9 | 1 | 25.87 |
| 72 | LTEKLIQK-LNDKVK-a4-b4 | OKP1 | Ame1 | 290 | 249 | 609.371 | 3 | -2.2 | 9 | 2 | 37.84 |
| 73 | LTEKLIQK-VKLNK-a4-b2 | OKP1 | Ame1 | 290 | 251 | 428.524 | 4 | 0.4 | 6 | 4 | 37.3 |
| 74 | RLTEKLIQK-VKLNK-a5-b2 | OKP1 | Ame1 | 290 | 251 | 467.55 | 4 | 1.2 | 7 | 2 | 36.85 |
| 75 | RLTEKLIQK-LNDKVK-a5-b4 | OKP1 | Ame1 | 290 | 249 | 496.307 | 4 | 1.7 | 8 | 2 | 34.66 |
| 76 | LTEKLIQK-VKLNKR-a4-b2 | OKP1 | Ame1 | 290 | 251 | 467.548 | 4 | -2.8 | 4 | 2 | 30.13 |
| 77 | LIQKDLHPVLNK-VKLNK-a4-b2 | OKP1 | Ame1 | 294 | 251 | 539.835 | 4 | -0.3 | 6 | 4 | 35.99 |
| 78 | LIQKDLHPVLNK-LNDKVK-a4-b4 | OKP1 | Ame1 | 294 | 249 | 568.593 | 4 | 1.2 | 9 | 4 | 35.92 |
| 79 | LLETNTVSALDSVFEKYEK-AKQLLATR-a16-b2 | OKP1 | CTF19 | 180 | 229 | 806.691 | 4 | -1.5 | 6 | 1 | 22.78 |
| 80 | LDKEEVLSLLPSLKEYTK-AKQLLATR-a14-b2 | OKP1 | CTF19 | 361 | 229 | 786.456 | 4 | 1.3 | 6 | 1 | 23.28 |
| 81 | AVENSDTAGSTKASSVFDR-TGIFQNLINLLKR-a12-b12 | SLI15 | CTF19 | 296 | 266 | 902.977 | 4 | -1.8 | 9 | 2 | 25.48 |
| 82 | LSPNIADISKPESR-TGIFQNLINLLKR-a10-b12 | SLI15 | CTF19 | 497 | 266 | 639.564 | 5 | -2.3 | 7 | 1 | 28.08 |
| 83 | LKESLAPFDNHVR-TGIFQNLINLLKR-a2-b12 | SLI15 | CTF19 | 576 | 266 | 798.952 | 4 | -1.4 | 10 | 2 | 27.55 |
| 84 | DTINKNTAFSTDNILATINTVDHR-TGIFQNLINLLKR-a5-b12 | SLI15 | CTF19 | 592 | 266 | 1.086.085 | 4 | -1.2 | 12 | 3 | 23.18 |
| 85 | SSGSIPKVR-YNLQKQFR-a7-b5 | SLI15 | IPL1 | 261 | 147 | 541.802 | 4 | -1.2 | 7 | 1 | 25.31 |
| 86 | GKNSRK-KATSSK-a2-b1 | SLI15 | IPL1 | 377 | 80 | 362.712 | 4 | 0.6 | 5 | 1 | 24.67 |
| 87 | SKHSSDVHK-KNIIHR-a2-b1 | SLI15 | IPL1 | 411 | 221 | 389.218 | 5 | -1.3 | 8 | 2 | 32.73 |
| 88 | NKNLTTSQTPHR-KNIIHR-a2-b1 | SLI15 | IPL1 | 467 | 221 | 463.661 | 5 | 0.7 | 11 | 1 | 32.4 |
| 89 | KLSPNIADISKPESR-YNLQKQFR-a1-b5 | SLI15 | IPL1 | 487 | 147 | 578.52 | 5 | 2.6 | 8 | 2 | 28.31 |
| 90 | MSHLEQDLKK-YNLQKQFR-a9-b5 | SLI15 | IPL1 | 561 | 147 | 616.328 | 4 | 0.2 | 7 | 2 | 25.19 |
| 91 | KQTSFSNDYK-YNLQKQFR-a1-b5 | SLI15 | IPL1 | 562 | 147 | 613.563 | 4 | -0.5 | 8 | 2 | 24.9 |
| 92 | LKESLAPFDNHVR-YNLQKQFR-a2-b5 | SLI15 | IPL1 | 576 | 147 | 552.699 | 5 | 0.3 | 12 | 2 | 37.22 |
| 93 | LKESLAPFDNHVR-KNIIHR-a2-b1 | SLI15 | IPL1 | 576 | 221 | 489.478 | 5 | -0.2 | 8 | 4 | 31.31 |
| 94 | LKESLAPFDNHVR-KATSSK-a2-b1 | SLI15 | IPL1 | 576 | 80 | 457.652 | 5 | 0.3 | 5 | 4 | 29.72 |
| 95 | LKESLAPFDNHVR-FGKVYCVR-a2-b3 | SLI15 | IPL1 | 576 | 117 | 539.089 | 5 | 2.2 | 9 | 1 | 24.46 |
| 96 | DTINKNTAFSTDNILATINTVDHR-YNLQKQFR-a5-b5 | SLI15 | IPL1 | 592 | 147 | 782.405 | 5 | 0.2 | 5 | 4 | 26.11 |
| 97 | ITKNNSPK-KSKELK-a3-b1 | SLI15 | OKP1 | 370 | 366 | 443.514 | 4 | -0.1 | 6 | 2 | 26.69 |
| 98 | KQTSFSNDYKDIR-GSKQLR-a10-b3 | SLI15 | OKP1 | 571 | 95 | 607.571 | 4 | -1.3 | 6 | 3 | 27.1 |
| 99 | DFNKSDDDQFR-LLYKLDLR-a4-b4 | Ame1 | Ame1 | 160 | 172 | 640.077 | 4 | -3 | 11 | 2 | 39.29 |
| 100 | DFNKSDDDQFR-KLLYK-a4-b1 | Ame1 | Ame1 | 160 | 168 | 730.035 | 3 | -1.5 | 5 | 4 | 32.65 |
| 101 | DFNKSDDDQFRK-LLYKLDLR-a4-b4 | Ame1 | Ame1 | 160 | 172 | 537.884 | 5 | -0.5 | 8 | 2 | 27.59 |
| 102 | LLYKLDLR-KLLYK-a4-b1 | Ame1 | Ame1 | 172 | 168 | 459.54 | 4 | -1.7 | 5 | 2 | 37.57 |
| 103 | LLYKLDLR-LNDKVK-a4-b4 | Ame1 | Ame1 | 172 | 249 | 472.538 | 4 | -1.1 | 6 | 1 | 34.24 |
| 104 | LLYKLDLR-VKLNK-a4-b2 | Ame1 | Ame1 | 172 | 251 | 591.372 | 3 | -2.2 | 5 | 2 | 29.46 |
| 105 | KEDLNQQIISVR-KEDLNQQIISVR-a1-b1 | Ame1 | Ame1 | 213 | 213 | 756.419 | 4 | 0.2 | 17 | 4 | 37.59 |
| 106 | KEDLNQQIISVR-LLYKLDLR-a1-b4 | Ame1 | Ame1 | 213 | 172 | 654.13 | 4 | -0.8 | 7 | 4 | 34.17 |
| 107 | KEDLNQQIISVR-LLYKLDLR-a1-b4 | Ame1 | Ame1 | 213 | 172 | 654.129 | 4 | -2.3 | 6 | 2 | 32.28 |
| 108 | KEDLNQQIISVR-LNDKVK-a1-b4 | Ame1 | Ame1 | 213 | 249 | 766.1 | 3 | -1.5 | 5 | 1 | 25.91 |
| 109 | NEIQELKAGK-LLYKLDLR-a7-b4 | Ame1 | Ame1 | 231 | 172 | 575.835 | 4 | -2.9 | 12 | 5 | 41.15 |
| 110 | NEIQELKAGK-LLYKLDLR-a7-b4 | Ame1 | Ame1 | 231 | 172 | 575.836 | 4 | -0.3 | 11 | 3 | 39.01 |
| 111 | NEIQELKAGK-VKLNK-a7-b2 | Ame1 | Ame1 | 231 | 251 | 467.776 | 4 | -3 | 6 | 4 | 37.5 |
| 112 | NEIQELKAGK-LNDKVK-a7-b4 | Ame1 | Ame1 | 231 | 249 | 496.533 | 4 | -3 | 5 | 7 | 33.06 |
| 113 | AGKDWHDLQNEQAK-LNDKVK-a3-b4 | Ame1 | Ame1 | 234 | 249 | 624.073 | 4 | -2.5 | 7 | 5 | 31.45 |
| 114 | AGKDWHDLQNEQAK-LNDKVK-a3-b4 | Ame1 | Ame1 | 234 | 249 | 624.073 | 4 | -2.6 | 6 | 2 | 27.44 |
| 115 | DWHDLQNEQAKLNDK-NEIQELKAGK-a11-b7 | Ame1 | Ame1 | 245 | 231 | 1.040.856 | 3 | -2.7 | 11 | 4 | 31.08 |
| 116 | DWHDLQNEQAKLNDK-LLYKLDLR-a11-b4 | Ame1 | Ame1 | 245 | 172 | 756.899 | 4 | -1.9 | 9 | 1 | 29.91 |
| 117 | FEDSTLLKWEILR-AKQLLATR-a8-b2 | CTF19 | CTF19 | 214 | 229 | 672.634 | 4 | -0.2 | 12 | 2 | 39.67 |
| 118 | NFQKCLLSLYEFDK-AKQLLATR-a4-b2 | CTF19 | CTF19 | 239 | 229 | 711.385 | 4 | -1.1 | 9 | 3 | 34.35 |
| 119 | NFQKCLLSLYEFDK-AKQLLATR-a4-b2 | CTF19 | CTF19 | 239 | 229 | 711.386 | 4 | 0.5 | 10 | 2 | 34.07 |
| 120 | TGIFQNLINLLKR-LQEEKDALLTR-a12-b5 | CTF19 | CTF19 | 266 | 61 | 746.43 | 4 | -0.9 | 16 | 2 | 39.18 |
| 121 | ISIALWKGGER-AKQLLATR-a7-b2 | CTF19 | CTF19 | 327 | 229 | 567.586 | 4 | -2.2 | 14 | 4 | 40.04 |
| 122 | NSLVNIKLNANSPSK-KTTTRPNTSR-a7-b1 | IPL1 | IPL1 | 10 | 19 | 580.322 | 5 | -0.6 | 5 | 2 | 26.34 |
| 123 | KTTTRPNTSR-INKPWR-a1-b3 | IPL1 | IPL1 | 19 | 31 | 528.797 | 4 | -1.9 | 6 | 2 | 28.16 |
| 124 | LNRLPVNNKK-KATSSK-a9-b1 | IPL1 | IPL1 | 64 | 80 | 391.635 | 5 | -0.6 | 8 | 5 | 32.96 |
| 125 | LPVNNKK-EKLNR-a6-b2 | IPL1 | IPL1 | 64 | 55 | 536.986 | 3 | -1.5 | 6 | 2 | 30.59 |
| 126 | LPVNNKK-KATSSK-a6-b1 | IPL1 | IPL1 | 64 | 80 | 393.484 | 4 | -1.7 | 5 | 2 | 27.39 |
| 127 | KATSSK-KNIIHR-a1-b1 | IPL1 | IPL1 | 80 | 221 | 385.481 | 4 | -1.8 | 9 | 11 | 33.06 |
| 128 | KATSSK-EKLNR-a1-b2 | IPL1 | IPL1 | 80 | 55 | 473.271 | 3 | -1.9 | 7 | 3 | 31.9 |
| 129 | KATSSK-LPKFK-a1-b3 | IPL1 | IPL1 | 80 | 95 | 464.282 | 3 | -1 | 7 | 2 | 30.8 |
| 130 | KATSSK-KNIIHR-a1-b1 | IPL1 | IPL1 | 80 | 221 | 385.481 | 4 | -1.5 | 8 | 1 | 30.73 |
| 131 | ATSSKMIHENK-KLPKFK-a5-b4 | IPL1 | IPL1 | 85 | 95 | 536.554 | 4 | -1.2 | 5 | 7 | 32.53 |
| 132 | MIHENKK-LPKFK-a6-b3 | IPL1 | IPL1 | 91 | 95 | 417.993 | 4 | -1.7 | 8 | 8 | 39.14 |
| 133 | MIHENKK-KNIIHR-a6-b1 | IPL1 | IPL1 | 91 | 221 | 364.21 | 5 | -1.2 | 9 | 4 | 34.82 |
| 134 | MIHENKK-KATSSK-a6-b1 | IPL1 | IPL1 | 91 | 80 | 415.229 | 4 | -2.1 | 9 | 6 | 34.43 |
| 135 | MIHENKK-KATSSK-a6-b1 | IPL1 | IPL1 | 91 | 80 | 553.303 | 3 | -1.5 | 7 | 9 | 34.26 |
| 136 | MIHENKK-LPKFK-a6-b3 | IPL1 | IPL1 | 91 | 95 | 417.993 | 4 | -2.5 | 6 | 4 | 32.26 |
| 137 | FKSLSLDDFELGK-MIHENKK-a2-b6 | IPL1 | IPL1 | 97 | 91 | 634.586 | 4 | 0.8 | 5 | 2 | 30.81 |
| 138 | FGKVYCVR-LGKGK-a3-b3 | IPL1 | IPL1 | 117 | 112 | 417.738 | 4 | -2.3 | 5 | 2 | 34.84 |
| 139 | FGKVYCVR-KLGKGK-a3-b4 | IPL1 | IPL1 | 117 | 112 | 449.761 | 4 | -3 | 7 | 1 | 34.42 |
| 140 | FGKVYCVR-KLGKGK-a3-b4 | IPL1 | IPL1 | 117 | 112 | 449.761 | 4 | -2.4 | 7 | 4 | 33.39 |
| 141 | STGYICALKVMEK-YNLQKQFR-a9-b5 | IPL1 | IPL1 | 133 | 147 | 684.108 | 4 | -1.2 | 9 | 4 | 29.42 |
| 142 | EEIIKYNLQK-FGKVYCVR-a5-b3 | IPL1 | IPL1 | 142 | 117 | 611.581 | 4 | -1.9 | 5 | 1 | 25.96 |
| 143 | YNLQKQFR-GKFGK-a5-b2 | IPL1 | IPL1 | 147 | 114 | 443.247 | 4 | -2.8 | 6 | 2 | 35.94 |
| 144 | YNLQKQFR-KNIIHR-a5-b1 | IPL1 | IPL1 | 147 | 221 | 403.632 | 5 | -2.1 | 9 | 3 | 34.01 |
| 145 | YNLQKQFR-KNIIHR-a5-b1 | IPL1 | IPL1 | 147 | 221 | 504.288 | 4 | -3 | 7 | 1 | 32.95 |
| 146 | YNLQKQFR-KATSSK-a5-b1 | IPL1 | IPL1 | 147 | 80 | 464.507 | 4 | -1.9 | 5 | 2 | 30.65 |
| 147 | SYGYFHDEKR-FGKVYCVR-a9-b3 | IPL1 | IPL1 | 175 | 117 | 617.551 | 4 | -2 | 11 | 5 | 36.47 |
| 148 | SYGYFHDEKR-FGKVYCVR-a9-b3 | IPL1 | IPL1 | 175 | 117 | 617.551 | 4 | -2.5 | 10 | 3 | 33.84 |
| 149 | LLKYDPK-DTTYKR-a3-b5 | IPL1 | IPL1 | 337 | 311 | 450 | 4 | -2.7 | 6 | 2 | 29.54 |
| 150 | LLKYDPK-DTTYKR-a3-b5 | IPL1 | IPL1 | 337 | 311 | 450 | 4 | -1.6 | 5 | 3 | 25.38 |
| 151 | LGDVKMHPWILR-NKPFWENK-a5-b2 | IPL1 | IPL1 | 350 | 359 | 533.688 | 5 | -1.8 | 11 | 12 | 39.64 |
| 152 | LGDVKMHPWILR-KNIIHR-a5-b1 | IPL1 | IPL1 | 350 | 221 | 477.278 | 5 | -1.2 | 8 | 8 | 37.73 |
| 153 | AKLENEEILPEQEWVLK-AQADIPATPIPYEPKK-a2-b15 | MCM21 | MCM21 | 99 | 95 | 1.315.372 | 3 | 1.5 | 11 | 3 | 29.48 |
| 154 | IKDASGEIFVDR-IKSNSWFLFK-a2-b2 | MCM21 | MCM21 | 189 | 229 | 689.874 | 4 | 2.1 | 12 | 4 | 37.83 |
| 155 | IKDASGEIFVDR-IKSNSWFLFK-a2-b2 | MCM21 | MCM21 | 189 | 229 | 689.871 | 4 | -1.5 | 10 | 5 | 36.57 |
| 156 | IKDASGEIFVDR-RIKSNSWFLFK-a2-b3 | MCM21 | MCM21 | 189 | 229 | 728.896 | 4 | -2.9 | 8 | 2 | 30.22 |
| 157 | TSQFEKPHYVLLK-QIFKDLEAK-a6-b4 | MCM21 | MCM21 | 218 | 286 | 564.513 | 5 | -2.5 | 11 | 13 | 33.64 |
| 158 | TSQFEKPHYVLLK-QIFKDLEAK-a6-b4 | MCM21 | MCM21 | 218 | 286 | 705.39 | 4 | -1.6 | 9 | 8 | 31.55 |
| 159 | TNKEEGQYHHK-GSKQLR-a3-b3 | OKP1 | OKP1 | 84 | 95 | 549.785 | 4 | -0.7 | 6 | 3 | 28.39 |
| 160 | TNKEEGQYHHK-STQSKK-a3-b5 | OKP1 | OKP1 | 84 | 62 | 547.276 | 4 | -2 | 5 | 1 | 27.07 |
| 161 | TNKEEGQYHHKGSK-IYSKK-a11-b4 | OKP1 | OKP1 | 92 | 202 | 484.453 | 5 | -2.9 | 6 | 2 | 29.65 |
| 162 | FEVGKESTGK-KVIQAEYR-a5-b1 | OKP1 | OKP1 | 103 | 132 | 557.05 | 4 | -1.7 | 7 | 3 | 27.84 |
| 163 | KVIQAEYR-QAKFPSR-a1-b3 | OKP1 | OKP1 | 132 | 222 | 659.701 | 3 | -1.9 | 9 | 4 | 31.38 |
| 164 | EMNQMTHGDNNEVKR-IYSKK-a14-b4 | OKP1 | OKP1 | 197 | 202 | 860.083 | 3 | -2 | 6 | 4 | 29.1 |
| 165 | LLEIILTKIK-IYSKK-a8-b4 | OKP1 | OKP1 | 213 | 202 | 490.567 | 4 | -2.5 | 5 | 3 | 33.86 |
| 166 | LTEKLIQK-TNNKKR-a4-b4 | OKP1 | OKP1 | 290 | 284 | 468.284 | 4 | 0 | 6 | 2 | 32.69 |
| 167 | LTEKLIQK-TNNKK-a4-b4 | OKP1 | OKP1 | 290 | 284 | 572.008 | 3 | -1.2 | 5 | 2 | 30.44 |
