# Supplementary material for: The COMA complex interacts with Cse4 and positions Sli15/Ipl1 at the budding yeast inner kinetochore
Source: eLife. 2019 May 21;8:e42879. doi: 10.7554/eLife.42879 (PMC6546395; doi:10.7554/eLife.42879)
Supplement: Supplementary file 3. [file elife-42879-supp3.docx]

**Supplementary file 3. Predicted and experimentally annotated protein domains and motifs depicted in protein cross-link networks.**

Native or *in vitro* reconstituted recombinant protein complexes were prepared and cross-linked as described in materials and methods. The identified inter- and intra-protein cross-links are visualized as protein network representations in Figure 1C and Figure 4A and are listed in Tables S3 and S4.

| Protein | Domain/Motif | Start | End | Reference |
| --- | --- | --- | --- | --- |
| AME1 | MIND binding | 1 | 15 | (Hornung et al., 2014) |
| AME1 | coiled coil | 177 | 272 | MARCOIL prediction  (Zimmermann et al., 2018) |
| AME1 | Okp1 binding | 129 | 247 | (Schmitzberger et al., 2017) |
| AME1 | Nkp1-Nkp2 binding | 268 | 292 | (Schmitzberger et al., 2017) |
| CENPA/  CSE4 | histone core | 113 | 227 | (Zhou et al., 2011) |
| CENPA/  CSE4 | CATD | 166 | 201 | (Zhou et al., 2011) |
| CHL4 | IML3 binding | 361 | 458 | (Hinshaw and Harrison, 2013) |
| CTF19 | RWD | 134 | 361 | Sequence alignment model / secondary structure prediction |
| H2A | histone core | 14 | 90 | (Wang et al., 2013) |
| H2B | histone core | 34 | 105 | (Wang et al., 2013) |
| H3 | histone core | 63 | 132 | (Wang et al., 2013) |
| H4 | histone core | 24 | 96 | (Wang et al., 2013) |
| IML3 | dimerization | 169 | 198 | (Hinshaw and Harrison, 2013) |
| IPL1 | kinase domain | 104 | 355 | Sequence alignment model |
| MCM21 | RWD | 156 | 368 | Sequence alignment model / secondary structure prediction |
| MIF2 | MTW1C binding | 1 | 35 | (Hornung et al., 2014) |
| MIF2 | signature motif | 238 | 312 | (Hornung et al., 2014) |
| MIF2 | IML3/CHL4 binding | 256 | 549 | (Hinshaw and Harrison, 2013) |
| MIF2 | cupin fold | 439 | 526 | (Hornung et al., 2014) |
| OKP1 | core domain | 166 | 211 | (Schmitzberger et al., 2017) |
| OKP1 | coiled coil | 183 | 290 | MARCOIL prediction |
| OKP1 | Ame1 binding | 234 | 264 | (Schmitzberger et al., 2017) |
| OKP1 | Ctf19-Mcm21 binding „segment1“ | 321 | 329 | (Schmitzberger et al., 2017) |
| OKP1 | coiled coil | 346 | 381 | MARCOIL prediction |
| OKP1 | Nkp1-Nkp2 | 357 | 375 | (Schmitzberger et al., 2017) |
| SLI15 | CEN targeting | 1 | 227 | (Campbell and Desai, 2013) |
| SLI15 | MT binding | 229 | 565 | (Fink et al., 2017) |
| SLI15 | SAH | 517 | 565 | (Fink et al., 2017) |
| SLI15 | IPL1 binding IN-box | 630 | 681 | (Adams et al., 2000, Kang et al., 2001) |

**References**

ADAMS, R. R., WHEATLEY, S. P., GOULDSWORTHY, A. M., KANDELS-LEWIS, S. E., CARMENA, M., SMYTHE, C., GERLOFF, D. L. & EARNSHAW, W. C. 2000. INCENP binds the Aurora-related kinase AIRK2 and is required to target it to chromosomes, the central spindle and cleavage furrow. *Curr Biol,* 10**,** 1075-8.

CAMPBELL, C. S. & DESAI, A. 2013. Tension sensing by Aurora B kinase is independent of survivin-based centromere localization. *Nature,* 497**,** 118-21.

FINK, S., TURNBULL, K., DESAI, A. & CAMPBELL, C. S. 2017. An engineered minimal chromosomal passenger complex reveals a role for INCENP/Sli15 spindle association in chromosome biorientation. *J Cell Biol,* 216**,** 911-923.

HINSHAW, S. M. & HARRISON, S. C. 2013. An Iml3-Chl4 heterodimer links the core centromere to factors required for accurate chromosome segregation. *Cell Rep,* 5**,** 29-36.

HORNUNG, P., TROC, P., MALVEZZI, F., MAIER, M., DEMIANOVA, Z., ZIMNIAK, T., LITOS, G., LAMPERT, F., SCHLEIFFER, A., BRUNNER, M., MECHTLER, K., HERZOG, F., MARLOVITS, T. C. & WESTERMANN, S. 2014. A cooperative mechanism drives budding yeast kinetochore assembly downstream of CENP-A. *J Cell Biol,* 206**,** 509-24.

KANG, J., CHEESEMAN, I. M., KALLSTROM, G., VELMURUGAN, S., BARNES, G. & CHAN, C. S. 2001. Functional cooperation of Dam1, Ipl1, and the inner centromere protein (INCENP)-related protein Sli15 during chromosome segregation. *J Cell Biol,* 155**,** 763-74.

SCHMITZBERGER, F., RICHTER, M. M., GORDIYENKO, Y., ROBINSON, C. V., DADLEZ, M. & WESTERMANN, S. 2017. Molecular basis for inner kinetochore configuration through RWD domain-peptide interactions. *EMBO J*.

WANG, F., LI, G., ALTAF, M., LU, C., CURRIE, M. A., JOHNSON, A. & MOAZED, D. 2013. Heterochromatin protein Sir3 induces contacts between the amino terminus of histone H4 and nucleosomal DNA. *Proc Natl Acad Sci U S A,* 110**,** 8495-500.

ZHOU, Z., FENG, H., ZHOU, B. R., GHIRLANDO, R., HU, K., ZWOLAK, A., MILLER JENKINS, L. M., XIAO, H., TJANDRA, N., WU, C. & BAI, Y. 2011. Structural basis for recognition of centromere histone variant CenH3 by the chaperone Scm3. *Nature,* 472**,** 234-7.

ZIMMERMANN, L., STEPHENS, A., NAM, S. Z., RAU, D., KUBLER, J., LOZAJIC, M., GABLER, F., SODING, J., LUPAS, A. N. & ALVA, V. 2018. A Completely Reimplemented MPI Bioinformatics Toolkit with a New HHpred Server at its Core. *J Mol Biol,* 430**,** 2237-2243.
