# Supplementary material for: The COMA complex interacts with Cse4 and positions Sli15/Ipl1 at the budding yeast inner kinetochore
Source: eLife. 2019 May 21;8:e42879. doi: 10.7554/eLife.42879 (PMC6546395; doi:10.7554/eLife.42879)
Supplement: Supplementary file 4. [file elife-42879-supp4.docx]

**Supplementary file 4. Plasmids used in this study**

| **Plasmid** | **description** | **source** |
| --- | --- | --- |
| pST44-Cse4 | pST44-6xHis-TEV-Flag-H2B-CSE4-H2A-H4 | Alwin Köhler  (Tan et al., 2005) |
| BSW1 | pST44-6xHis-TEV-Flag-H2B-CSE4∆2-30-H2A-H4 | this study |
| BSW2 | pST44-6xHis-TEV-Flag-H2B-CSE4∆31-60-H2A-H4 | this study |
| BSW4 | pST44-6xHis-TEV-Flag-H2B-CSE4∆34-46-H2A-H4 | this study |
| BSW5 | pST44-6xHis-TEV-Flag-H2B-CSE4∆48-61-H2A-H4 | this study |
| pST44-H3 | pST44-6xHis-TEV-Flag-H2B-H3-H2A-H4 | Alwin Köhler |
| BJF6 | pLIB-MIF2-6xHis-6xFlag | this study |
| BMP37 | pETDuet-6xHis-CHL4/IML3 | this study |
| pPH74 | pST39-OKP1-AME1-6xHis | Stefan Westermann |
| BJF26 | pST39-OKP1∆123-147-AME1-6xHis | this study |
| BJF27 | pST39-OKP1∆140-170-AME1-6xHis | this study |
| BJF28 | pST39-OKP1∆163-187-AME1-6xHis | this study |
| pSW661 | pST39-CTF19-MCM21-6xHis | Stefan Westermann |
| BJF25 | pST39-CTF19∆C270-369 -MCM21-6xHis | this study |
| BJF7 | pBIG1-MCM21-6xHis-6xFlag/CTF19 | this study |
| BJF50 | pBIG1-AME1-6xHis-6xFlag /OKP1 | this study |
| BMP75 | pBIG1-MTW1-NNF1-NSL1-DSN1-2xStrep | this study |
| BJF10 | pBIG-CTF3-MCM16-MCM22 | this study |
| BMS52 | pETDuet-6xHis-CNN1-WIP1-1xFlag | this study |
| BMS64 | pETDuet-6xHis-NKP1-NKP2 | this study |
| BMS65 | pETDuet-MHF2-MHF1-1xStrep | this study |
| BJF1 | pBIG1-SLI15-2xStrep-HA-6xHis/IPL1 | this study |
| BSS93 | pRS313-pCSE4-3xFlag-CSE4 | this study |
| BSS94 | pRS313-pCSE4-3xFlag-CSE4∆31-60 | this study |
| BSS95 | pRS313-pCSE4-3xFlag-CSE4∆62-94 | this study |
| BSS96 | pRS313-pCSE4-3xFlag-CSE4∆34-46 | this study |
| BSS97 | pRS313-pCSE4-3xFlag-CSE4∆48-61 | this study |
| BSS134 | pRS313-pCTF19-CTF19WT-SLI15∆2-228-6xHis-7xFlag | this study |
| BSS146 | pRS313-pAME1-AME1-SLI15∆2-228-6xHis-7xFlag | this study |
| BSS142 | pRS313-pOKP1-OKP1-SLI15∆2-228-6xHis-7xFlag | this study |
| BSS145 | pRS313-pMIF2-MIF2-SLI15∆2-228-6xHis-7xFlag | this study |
| BSS143 | pRS313-pCTF3-CTF3-SLI15∆2-228-6xHis-7xFlag | this study |
| BSS144 | pRS313-pMTW1-MTW1-SLI15∆2-228-6xHis-7xFlag | this study |
| BSS141 | pRS313-pDSN1-DSN1-SLI15∆2-228-6xHis-7xFlag | this study |
| BSS147 | pRS313-pCNN1-CNN1-SLI15∆2-228-6xHis-7xFlag | this study |
| BSS1 | pRS313-pSLI15-SLI15-6xHis-6xFlag | this study |
| BSS15 | pRS313-pSLI15-SLI15∆SAH-6xHis-6xFlag | this study |
| BSS2 | pRS313-pSLI15-SLI15∆2-228-6xHis-6xFlag | this study |
| BSS16 | pRS313-pSLI15-SLI15∆2-228∆SAH-6xHis-6xFlag | this study |
| BSS129 | pRS313-pCTF19-CTF19-6xHis-7xFlag | this study |
| BSS159 | pRS313-pCTF19-3xMyc-CTF19∆2-30-6xHis-7xFlag | this study |
| BSS76 | pRS313-pAME1-AME1-6xHis-7xFlag | this study |
| BSS169 | pRS313-pOKP1-OKP1-6xHis-6xFlag | this study |
| BSS172 | pRS313-pOKP1-OKP1∆123-147-6xHis-6xFlag | this study |
| BSS174 | pRS313-pOKP1-OKP1∆163-187-6xHis-6xFlag | this study |
| BSS165 | pRS313-pAME1-AME1-SLI15∆2-228-∆INbox(626-698)-6xHis-7xFlag | this study |
| BSS167 | pRS313-pAME1-AME1-SLI15∆2-228-∆SAH(516-575)-6xHis-7xFlag | this study |
| BSS164 | pRS313-pOKP1-OKP1-SLI15∆2-228-∆INbox(626-698)-6xHis-7xFlag | this study |
| BSS166 | pRS313-pOKP1-OKP1-SLI15∆2-228-∆SAH(516-575)-6xHis-7xFlag | this study |
| BSS175 | pRS313-pAME1-AME1-CTF19-6xHis-7xFlag | this study |
| BSS176 | pRS313-pAME1-AME1-CTF19∆C270-369 -6xHis-7xFlag | this study |
| BSS177 | pRS313-pOKP1-OKP1-CTF19-6xHis-7xFlag | this study |
| BSS178 | pRS313-pOKP1-OKP1-CTF19∆C270-369 -6xHis-7xFlag | this study |
| BSS212 | pRS313-pCTF19-CTF19-OKP1-6xHis-7xFlag | this study |
| BSS213 | pRS313-pCTF19-CTF19∆C270-369-OKP1-6-xHis-7xFlag | this study |
| BSS214 | pRS313-pCTF19-CTF19-OKP1-GFP | this study |
| BSS215 | pRS313-pCTF19-CTF19∆C270-369-OKP1-GFP | this study |
| pYCF1/CEN3.L | YRp14/TEL cassette (pYCF1) with a CEN3 insert | (Spencer et al., 1990) |

**References**

SPENCER, F., GERRING, S. L., CONNELLY, C. & HIETER, P. 1990. Mitotic chromosome transmission fidelity mutants in Saccharomyces cerevisiae. *Genetics,* 124**,** 237-49.

TAN, S., KERN, R. C. & SELLECK, W. 2005. The pST44 polycistronic expression system for producing protein complexes in Escherichia coli. *Protein Expr Purif,* 40**,** 385-95.
