# Supplementary material for: The COMA complex interacts with Cse4 and positions Sli15/Ipl1 at the budding yeast inner kinetochore
Source: eLife. 2019 May 21;8:e42879. doi: 10.7554/eLife.42879 (PMC6546395; doi:10.7554/eLife.42879)
Supplement: Supplementary file 5. [file elife-42879-supp5.docx]

**Supplementary file 5. Yeast strains used in this study.**

All strains are isogenic with the S288c background

| **strain** | **genotype** |
| --- | --- |
| YSS225 | MAT a, tor1-1, fpr1::loxP-Leu2-loxP, RPL13A-2xFKBP12::lox-TRP1-loxP, CSE4-FRB::KanMX |
| YSS226 | MAT a, tor1-1, fpr1::loxP-Leu2-loxP, RPL13A-2xFKBP12::lox-TRP1-loxP, CSE4-FRB::KanMX, pRS313-pCSE4-3xFlag-CSE4 |
| YSS227 | MAT a, tor1-1, fpr1::loxP-Leu2-loxP, RPL13A-2xFKBP12::lox-TRP1-loxP, CSE4-FRB::KanMX, pRS313-pCSE4-3xFlag-CSE4∆62-94 |
| YSS228 | MAT a, tor1-1, fpr1::loxP-Leu2-loxP, RPL13A-2xFKBP12::lox-TRP1-loxP, CSE4-FRB::KanMX, pRS313-pCSE4-3xFlag-CSE4∆31-60 |
| YSS229 | MAT a, tor1-1, fpr1::loxP-Leu2-loxP, RPL13A-2xFKBP12::lox-TRP1-loxP, CSE4-FRB::KanMX, pRS313-pCSE4-3xFlag-CSE4∆34-46 |
| YSS230 | MAT a, tor1-1, fpr1::loxP-Leu2-loxP, RPL13A-2xFKBP12::lox-TRP1-loxP, CSE4-FRB::KanMX, pRS313-pCSE4-3xFlag-CSE4∆48-61 |
| YSS1 | MAT a, tor1-1, fpr1::loxP-Leu2-loxP, RPL13A-2xFKBP12::lox-TRP1-loxP, Sli15-FRB::KanMX |
| YSS69 | MAT a, tor1-1, fpr1::loxP-Leu2-loxP, RPL13A-2xFKBP12::lox-TRP1-loxP, Sli15-FRB::KanMX, pRS313-pSLI15-SLI15-6xHis-6xFlag |
| YSS70 | MAT a, tor1-1, fpr1::loxP-Leu2-loxP, RPL13A-2xFKBP12::lox-TRP1-loxP, Sli15-FRB::KanMX, pRS313-pSLI15-SLI15∆2-228-6xHis-6xFlag |
| YSS81 | MAT a, tor1-1, fpr1::loxP-Leu2-loxP, RPL13A-2xFKBP12::lox-TRP1-loxP, Sli15-FRB::KanMX, pRS313-pSLI15-SLI15∆SAH-6xHis-6xFlag |
| YSS87 | MAT a, tor1-1, fpr1::loxP-Leu2-loxP, RPL13A-2xFKBP12::lox-TRP1-loxP, Sli15-FRB::KanMX, pRS313-pSLI15-SLI15∆2-228∆SAH-6xHis-6xFlag |
| YSS213 | MAT a, tor1-1, fpr1::loxP-Leu2-loxP, RPL13A-2xFKBP12::lox-TRP1-loxP, CTF19-FRB::KanMX |
| YSS216 | MAT a, tor1-1, fpr1::loxP-Leu2-loxP, RPL13A-2xFKBP12::lox-TRP1-loxP, CTF19-FRB::KanMX, sli15Δ2-228::hphNT1 |
| YSS321 | MAT a, tor1-1, fpr1::loxP-Leu2-loxP, RPL13A-2xFKBP12::lox-TRP1-loxP, CTF19-FRB::KanMX, pRS313-pCTF19-CTF19-6xHis-7xFlag |
| YSS325 | MAT a, tor1-1, fpr1::loxP-Leu2-loxP, RPL13A-2xFKBP12::lox-TRP1-loxP, CTF19-FRB::KanMX, sli15Δ2-228::hphNT1, pRS313-pCTF19-CTF19-6xHis-7xFlag |
| YSS301 | MAT a, tor1-1, fpr1::loxP-Leu2-loxP, RPL13A-2xFKBP12::lox-TRP1-loxP, CTF19-FRB::KanMX, sli15Δ2-228::hphNT1, pRS313-pCTF19-CTF19WT-SLI15∆2-228-6xHis-7xFlag |
| YSS348 | MAT a, tor1-1, fpr1::loxP-Leu2-loxP, RPL13A-2xFKBP12::lox-TRP1-loxP, CTF19-FRB::KanMX, sli15Δ2-228::hphNT1, pRS313-pCTF19-3xMyc-CTF19∆2-30-6xHis-7xFlag |
| YSS334 | MAT a, tor1-1, fpr1::loxP-Leu2-loxP, RPL13A-2xFKBP12::lox-TRP1-loxP, CTF19-FRB::KanMX, sli15Δ2-228::hphNT1, pRS313-pAME1-AME1-6xHis-7xFlag |
| YSS335 | MAT a, tor1-1, fpr1::loxP-Leu2-loxP, RPL13A-2xFKBP12::lox-TRP1-loxP, CTF19-FRB::KanMX, sli15Δ2-228::hphNT1, pRS313-pAME1-AME1-SLI15∆2-228-6xHis-7xFlag |
| YSS336 | MAT a, tor1-1, fpr1::loxP-Leu2-loxP, RPL13A-2xFKBP12::lox-TRP1-loxP, CTF19-FRB::KanMX, sli15Δ2-228::hphNT1, pRS313-pAME1-AME1-SLI15∆2-228-∆INbox(626-698)-6xHis-7xFlag |
| YSS337 | MAT a, tor1-1, fpr1::loxP-Leu2-loxP, RPL13A-2xFKBP12::lox-TRP1-loxP, CTF19-FRB::KanMX, sli15Δ2-228::hphNT1, pRS313-pAME1-AME1-SLI15∆2-228-∆SAH(516-575)-6xHis-7xFlag |
| YSS92 | MAT a, tor1-1, fpr1::loxP-Leu2-loxP, RPL13A-2xFKBP12::lox-TRP1-loxP, OKP1-FRB::KanMX |
| YSS351 | MAT a, tor1-1, fpr1::loxP-Leu2-loxP, RPL13A-2xFKBP12::lox-TRP1-loxP, OKP1-FRB::KanMX, pRS313-pOKP1-OKP1-6xHis-6xFlag |
| YSS394 | MAT a, tor1-1, fpr1::loxP-Leu2-loxP, RPL13A-2xFKBP12::lox-TRP1-loxP, OKP1-FRB::KanMX, pRS313-pOKP1-OKP1∆122-147-6xHis-6xFlag |
| YSS395 | MAT a, tor1-1, fpr1::loxP-Leu2-loxP, RPL13A-2xFKBP12::lox-TRP1-loxP, OKP1-FRB::KanMX, pRS313-pOKP1-OKP1∆163-187-6xHis-6xFlag |
| YSS342 | MAT a, tor1-1, fpr1::loxP-Leu2-loxP, RPL13A-2xFKBP12::lox-TRP1-loxP, CTF19-FRB::KanMX, sli15Δ2-228::hphNT1, pRS313-pOKP1-OKP1-6xHis-6xFlag |
| YSS343 | MAT a, tor1-1, fpr1::loxP-Leu2-loxP, RPL13A-2xFKBP12::lox-TRP1-loxP, CTF19-FRB::KanMX, sli15Δ2-228::hphNT1, pRS313-pOKP1-OKP1-SLI15∆2-228-6xHis-7xFlag |
| YSS344 | MAT a, tor1-1, fpr1::loxP-Leu2-loxP, RPL13A-2xFKBP12::lox-TRP1-loxP, CTF19-FRB::KanMX, sli15Δ2-228::hphNT1, pRS313-pOKP1-OKP1-SLI15∆2-228-∆INbox(626-698)-6xHis-7xFlag |
| YSS345 | MAT a, tor1-1, fpr1::loxP-Leu2-loxP, RPL13A-2xFKBP12::lox-TRP1-loxP, CTF19-FRB::KanMX, sli15Δ2-228::hphNT1, pRS313-pOKP1-OKP1-SLI15∆2-228-∆SAH(516-575)-6xHis-7xFlag |
| YSS315 | MAT a, tor1-1, fpr1::loxP-Leu2-loxP, RPL13A-2xFKBP12::lox-TRP1-loxP, CTF19-FRB::KanMX, sli15Δ2-228::hphNT1, pRS313-pMIF2-MIF2-SLI15∆2-228-6xHis-7xFlag |
| YSS313 | MAT a, tor1-1, fpr1::loxP-Leu2-loxP, RPL13A-2xFKBP12::lox-TRP1-loxP, CTF19-FRB::KanMX, sli15Δ2-228::hphNT1, pRS313-pCTF3-CTF3-SLI15∆2-228-6xHis-7xFlag |
| YSS314 | MAT a, tor1-1, fpr1::loxP-Leu2-loxP, RPL13A-2xFKBP12::lox-TRP1-loxP, CTF19-FRB::KanMX, sli15Δ2-228::hphNT1, pRS313-pMTW1-MTW1-SLI15∆2-228-6xHis-7xFlag |
| YSS311 | MAT a, tor1-1, fpr1::loxP-Leu2-loxP, RPL13A-2xFKBP12::lox-TRP1-loxP, CTF19-FRB::KanMX, sli15Δ2-228::hphNT1, pRS313-pDSN1-DSN1-SLI15∆2-228-6xHis-7xFlag |
| YSS317 | MAT a, tor1-1, fpr1::loxP-Leu2-loxP, RPL13A-2xFKBP12::lox-TRP1-loxP, CTF19-FRB::KanMX, sli15Δ2-228::hphNT1, pRS313-pCNN1-CNN1-SLI15∆2-228-6xHis-7xFlag |
| YSS366 | MAT a, tor1-1, fpr1::loxP-Leu2-loxP, RPL13A-2xFKBP12::lox-TRP1-loxP, CTF19-FRB::KanMX, sli15Δ2-228::hphNT1, pRS313-pSLI15-SLI15∆2-228-6xHis-6xFlag |
| YSS399 | MAT a, tor1-1, fpr1::loxP-Leu2-loxP, RPL13A-2xFKBP12::lox-TRP1-loxP, CTF19-FRB::KanMX, sli15Δ2-228::hphNT1, pRS313-pAME1-AME1-CTF19-6xHis-7xFlag |
| YSS400 | MAT a, tor1-1, fpr1::loxP-Leu2-loxP, RPL13A-2xFKBP12::lox-TRP1-loxP, CTF19-FRB::KanMX, sli15Δ2-228::hphNT1, pRS313-pAME1-AME1-CTF19∆C270-369-6xHis-7xFlag |
| YSS405 | MAT a, tor1-1, fpr1::loxP-Leu2-loxP, RPL13A-2xFKBP12::lox-TRP1-loxP, CTF19-FRB::KanMX, pRS313-pOKP1-OKP1-CTF19-6xHis-7xFlag |
| YSS406 | MAT a, tor1-1, fpr1::loxP-Leu2-loxP, RPL13A-2xFKBP12::lox-TRP1-loxP, CTF19-FRB::KanMX, pRS313-pOKP1-OKP1-CTF19∆C270-369-6xHis-7xFlag |
| YSS401 | MAT a, tor1-1, fpr1::loxP-Leu2-loxP, RPL13A-2xFKBP12::lox-TRP1-loxP, CTF19-FRB::KanMX, sli15Δ2-228::hphNT1, pRS313-pOKP1-OKP1-CTF19-6xHis-7xFlag |
| YSS402 | MAT a, tor1-1, fpr1::loxP-Leu2-loxP, RPL13A-2xFKBP12::lox-TRP1-loxP, CTF19-FRB::KanMX, sli15Δ2-228::hphNT1, pRS313-pOKP1-OKP1-CTF19∆C270-369-6xHis-7xFlag |
| YSS511 | MAT a, tor1-1, fpr1::loxP-Leu2-loxP, RPL13A-2xFKBP12::lox-TRP1-loxP, CTF19-FRB::KanMX, pRS313-pCTF19-CTF19-Okp1-6xHis-7xFlag |
| YSS512 | MAT a, tor1-1, fpr1::loxP-Leu2-loxP, RPL13A-2xFKBP12::lox-TRP1-loxP, CTF19-FRB::KanMX, pRS313-pCTF19-CTF19∆C270-369-Okp1-6xHis-7xFlag |
| YSS513 | MAT a, tor1-1, fpr1::loxP-Leu2-loxP, RPL13A-2xFKBP12::lox-TRP1-loxP, CTF19-FRB::KanMX, sli15Δ2-228::hphNT1, pRS313-pCTF19-CTF19-Okp1-6xHis-7xFlag |
| YSS514 | MAT a, tor1-1, fpr1::loxP-Leu2-loxP, RPL13A-2xFKBP12::lox-TRP1-loxP, CTF19-FRB::KanMX, sli15Δ2-228::hphNT1, pRS313-pCTF19-CTF19∆C270-369-Okp1-6xHis-7xFlag |
| YSS515 | MAT a, tor1-1, fpr1::loxP-Leu2-loxP, RPL13A-2xFKBP12::lox-TRP1-loxP, CTF19-FRB::KanMX, NDC80-mCherry::natNT2, pRS313-pCTF19-CTF19-Okp1-GFP |
| YSS516 | MAT a, tor1-1, fpr1::loxP-Leu2-loxP, RPL13A-2xFKBP12::lox-TRP1-loxP, CTF19-FRB::KanMX, NDC80-mCherry::natNT2, pRS313-pCTF19-CTF19∆C270-369-OKP1-GFP |
| YSS255 | MAT a, tor1-1, fpr1::loxP-Leu2-loxP, RPL13A-2xFKBP12::lox-TRP1-loxP, NDC80-mCherry::natNT2, CTF19-GFP::HIS |
| YSS256 | MAT a, tor1-1, fpr1::loxP-Leu2-loxP, RPL13A-2xFKBP12::lox-TRP1-loxP, NDC80-mCherry::natNT2, CTF19∆C270-369-GFP::HIS |
